# Problem-Solving Skills Training for Parents of Children With Chronic Health Conditions: A Systematic Review and Meta-Analysis

**DOI:** 10.1001/jamapediatrics.2023.5753

**Published:** 2024-01-02

**Authors:** Tianji Zhou, Yuanhui Luo, Wenjin Xiong, Zhenyu Meng, Hanyi Zhang, Jingping Zhang

**Affiliations:** 1 Xiangya School of Nursing, Central South University, Changsha, Hunan, China; 2 Xiangya Hospital, Central South University, Changsha, Hunan, China

## Abstract

**Importance:**

Problem-solving skills training (PSST) has a demonstrated potential to improve psychosocial well-being for parents of children with chronic health conditions (CHCs), but such evidence has not been fully systematically synthesized.

**Objective:**

To evaluate the associations of PSST with parental, pediatric, and family psychosocial outcomes.

**Data Sources:**

Six English-language databases (PubMed, Embase, CINAHL, PsycINFO, Web of Science, and Cochrane Library), 3 Chinese-language databases (China National Knowledge Infrastructure, China Science and Technology Journal Database, and Wanfang), gray literature, and references were searched from inception to April 30, 2023.

**Study Selection:**

Randomized clinical trials (RCTs) that performed PSST for parents of children with CHCs and reported at least 1 parental, pediatric, or family psychosocial outcome were included.

**Data Extraction and Synthesis:**

Study selection, data extraction, and quality assessment were conducted independently by 2 reviewers. Data were pooled for meta-analysis using the standardized mean difference (SMD) by the inverse variance method or a random-effects model. Subgroup analyses of children- and intervention-level characteristics were conducted.

**Main Outcomes and Measures:**

The psychosocial outcomes of the parents, their children, and their families, such as problem-solving skills, negative affectivity, quality of life (QOL), and family adaptation.

**Results:**

The systematic review included 23 RCTs involving 3141 parents, and 21 of these trials were eligible for meta-analysis. There was a significant association between PSST and improvements in parental outcomes, including problem-solving skills (SMD, 0.43; 95% CI, 0.27-0.58), depression (SMD, −0.45; 95% CI, −0.66 to −0.23), distress (SMD, −0.61; 95% CI, −0.81 to −0.40), posttraumatic stress (SMD −0.39; 95% CI, −0.48 to −0.31), parenting stress (SMD, −0.62; 95% CI, −1.05 to −0.19), and QOL (SMD, 0.45; 95% CI, 0.15-0.74). For children, PSST was associated with better QOL (SMD, 0.76; 95% CI, 0.04-1.47) and fewer mental problems (SMD, −0.51; 95% CI, −0.68 to −0.34), as well as with less parent-child conflict (SMD, −0.38; 95% CI, −0.60 to −0.16). Subgroup analysis showed that PSST was more efficient for parents of children aged 10 years or younger or who were newly diagnosed with a CHC. Significant improvements in most outcomes were associated with PSST delivered online.

**Conclusions and Relevance:**

These findings suggest that PSST for parents of children with CHCs may improve the psychosocial well-being of the parents, their children, and their families. Further high-quality RCTs with longer follow-up times and that explore physical and clinical outcomes are encouraged to generate adequate evidence.

## Key Points

**Question:**

What is the association between problem-solving skills training (PSST) for parents of children with chronic health conditions and psychosocial outcomes of the parents, their children, and their families?

**Findings:**

In this systematic review and meta-analysis of 23 randomized clinical trials including 3141 parents, PSST was associated with improvements in parental problem-solving skills; decreased parental depression, distress, posttraumatic stress, and parenting stress; better quality of life for both parents and children; fewer pediatric mental problems; and less parent-child conflict.

**Meaning:**

These findings suggest that PSST should be an active component of and serve as an emerging perspective for psychosocial interventions for parents of children with chronic health conditions.

## Teaser

This systematic review and meta-analysis evaluates the association of problem-solving skills training with psychosocial outcomes for parents of children with chronic health conditions.

## Introduction

Childhood chronic health conditions (CHCs) include physical, developmental, behavioral, or emotional conditions with an expected duration of more than 3 months or the impossibility of cure.^1^ Approximately 37% of children have at least 1 current or lifelong health condition.^2^ The diagnosis of a childhood CHC and its prolonged treatments are profoundly unsettling experiences for children and their families, especially their parents.^3,4,5^ Compared with parents of healthy children, parents of children with CHCs have reported worse mental health (more depression, anxiety, and posttraumatic stress),^6,7,8^ significant stress and burden,^9,10^ and a poorer quality of life (QOL).^6,11^ Considering that parental psychosocial outcomes are strongly associated with children’s health and family adaptation,^12,13,14^ interventions to improve parents’ well-being may have synergistic benefits for the whole family. Parental problem-solving skills, which are associated with parents’ well-being, are general coping skills applicable to a variety of difficult situations commonly encountered during the treatment of childhood CHCs.^15^ With better problem-solving skills, parents could become more self-assured to address children’s health concerns, fully use resources to cope with stress, and collaborate to address challenges presented by daily care, thereby improving family adaptation and children’s health outcomes.^16^ However, nearly one-half of parents lack problem-solving skills, especially the ability to solve daily problems related to their children’s complex treatment processes,^3^ which may eventually perpetuate negative outcomes for parental and child well-being.^17,18^

Problem-solving skills training (PSST) is an effective intervention to improve problem-solving skills and decrease negative affectivity.^19,20^ Based on the problem-solving therapy approaches of D’Zurilla and colleagues,^20,21^ PSST includes 2 essential components: establishing a positive problem orientation and mastering the systematic steps to solve problems. The training has long been established as being effective in adults with chronic illness and their caregivers,^22,23^ which theoretically could have broad outcomes for parents of children with CHCs due to the long-term nature and equally multiple, intensive, and ongoing stressors across childhood CHCs. Problem-solving skills training is a cognitive-behavioral process by which parents can identify and create problem-focused strategies to buffer the outcomes of stressful events and improve coping, thus preventing episodes of negative affectivity by effectively solving various children’s disease-related problems.^15,21^ These problem-solving strategies, while possibly differing in specifics, are beneficial in helping parents to cope with significant stressors inherent to each CHC. Preliminary studies have shown the efficacy of PSST in enhancing problem-solving skills and alleviating depression symptoms for parents, although the majority of such studies have had small sample sizes. Moreover, these studies only considered improved parental well-being, and most did not show significant changes in pediatric or family adaptation outcomes.^15,24^ In addition, although previous reviews of PSST have explored the effectiveness of psychosocial interventions for parents of children with CHCs, they had limited specificity.^19,25,26,27,28^ To address these gaps, we evaluated the associations between PSST for parents of children with CHCs and parental, pediatric, and family psychosocial outcomes.

## Methods

The study protocol for this systematic review and meta-analysis has been registered with PROSPERO (CRD42023424077). The revised Preferred Reporting Items for Systematic Reviews and Meta-Analyses (PRISMA) guideline^29^ was followed to report the findings.

### Data Sources and Search Strategies

A systematic search was performed across 6 English-language databases (PubMed, Embase, CINAHL, PsycINFO, Web of Science, and Cochrane Library) and 3 Chinese-language databases (China National Knowledge Infrastructure, China Science and Technology Journal Database, and Wanfang) from inception to April 30, 2023. The search strategies applied a combination of Medical Subject Heading terms and keywords, and the following constructs were used: child AND chronic health conditions AND parents AND PSST. The full search string for each database is provided in eTable 1 in Supplement 1. Gray literature was searched using OpenGrey, Mednar, and the World Health Organization’s search portal. We also screened reference lists of included studies to identify potentially eligible articles.

### Eligibility Criteria

The population, intervention, comparator, outcomes, and study design framework was used to define the inclusion and exclusion criteria (Table 1). Eligible studies were RCTs that performed PSST for parents of children with CHCs and reported at least 1 psychosocial outcome of parents, children, or their families.

**Table 1.** Inclusion and Exclusion Criteria

	Inclusion criteria	Exclusion criteria
Population	Parents (mothers and/or fathers) of children aged <18 y Children with any childhood-onset CHC operationally defined as a physical, developmental, behavioral, or emotional condition that had an expected duration of at least 3 mo or the impossibility of cure.^1^	Children with CHCs who died Parents with cognitive impairments or psychological disorders Grandparents, siblings, teachers, or other professionals as the main participants
Intervention	An intervention was considered problem-solving skills training when problem-solving was the sole intervention or core element, including the following steps: problem definition and formulation, generation of alternative solutions, decision making, solution implementation, and evaluation.^21^ Other techniques and devices were acceptable when they were designed to support or enhance the problem-solving component.	Studies with no problem-solving components. Interventions that targeted children with CHCs without directly being implemented with parents
Comparator	Control conditions including wait-list control, usual care, psychoeducation control, psychotherapy modalities, etc	No restrictions
Outcomes	Studies reporting on at least 1 psychosocial outcome verified by the parents, their children with CHCs, or their families, including depression, distress, anxiety, burden, self-efficacy, problem-solving skills, quality of life, family adaptation, family conflict, family cohesion, and family functioning	Studies focusing on the outcomes of the feasibility of intervention delivery, eg, experiences, attitudes, completion rates, and cost-benefit analyses
Study design	Randomized clinical trials written in English or Chinese	Protocols, reviews, conference abstracts, quasi-experimental studies, case studies, or exclusively qualitative studies

Abbreviation: CHC, chronic health condition.

### Study Selection and Data Extraction

All identified articles were imported into EndNote, version 20.0 (Clarivate Analytics) to eliminate duplications. Title and abstract screening and full-text review were performed independently using the web-based software Rayyan^30^ by 2 reviewers (T.Z. and W.X.). Data extraction was conducted in duplicate by the 2 reviewers and checked by another reviewer (Y.L.). Information was extracted using a predesigned worksheet, including publication details, population demographics (pediatric [age, medical condition, and illness duration] and parental [age, sex, race and ethnicity]), intervention and control group details (approach, mode, number of sessions, and duration), and psychosocial outcomes and measures.

We included only the postintervention data in the meta-analysis, as follow-up data were not reported consistently enough to achieve proper homogeneity. When both parents and children reported a psychosocial outcome of children, we prioritized extracting the parent-reported data, as they were more reliable. If multiple records were available for the same trial, we collected all relevant data and analyzed them as a single study. Corresponding authors were contacted via email to retrieve missing data.

### Quality Assessment

The risk of bias for the included studies was assessed independently by 2 reviewers (T.Z. and W.X.) according to the revised Cochrane risk-of-bias tool, version 2.0,^31^ which includes 5 domains: randomization process, deviations from intended interventions, missing outcome data, measurement of the outcome, and selection of the reported result. We judged the studies to be low risk, of some concern, or high risk. Additionally, the Grading of Recommendations, Assessment, Development, and Evaluations (GRADE)^32^ framework was applied to assess the certainty of the evidence for all outcomes. The certainty was categorized as high, moderate, low, or very low based on the risk of bias, inconsistency, imprecision, indirectness, and publication bias.^33,34^ Any disagreements in the study selection, data extraction, and quality assessment processes were resolved through discussion to reach a consensus, and if conflicts persisted, they were arbitrated by a third reviewer (Y.L.).

### Statistical Analysis

Statistical analyses were performed using Stata, version 16 software (StataCorp LLC). We conducted a meta-analysis only when 2 or more intervention studies were available with similar participants and outcomes. The psychosocial outcomes included in this review were measured by different scales; therefore, the effect size is presented as the standardized mean difference (SMD) with 95% CI.^35^ Statistical heterogeneity was assessed using both the χ^2^ test and *I*^2^ statistic.^36^ The inverse variance method (*P* ≥ .10 and *I^2^*<50%) or a random-effects model (*P* < .10 or *I^2^*≥50%) was applied based on the *P* and *I*^2^ values. Subgroup analyses were performed for children’s and intervention characteristics. In addition, we conducted leave-one-out sensitivity analyses to examine the consistent associations between PSST and all identified outcomes. We also used funnel plots and Egger test to evaluate the publication bias for analyses with at least 10 studies.^37^ The threshold for statistical significance was set at a 2-sided *P* < .05. The most recent analysis update was performed between October 10 and 20, 2023.

## Results

### Study Selection

**Figure 1.**
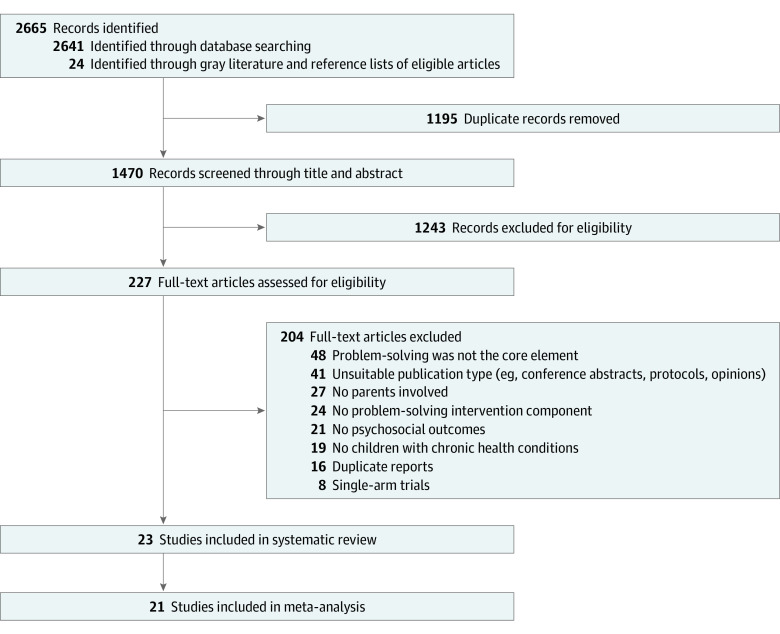
Flowchart for Study Selection

The initial comprehensive search yielded 2665 publications: 2641 from 9 databases and an additional 24 from gray literature and reference list review. After removing 1195 duplicates and screening 1470 titles and abstracts, 227 full-text articles were assessed for eligibility. Ultimately, 23 eligible RCTs^38,39,40,41,42,43,44,45,46,47,48,49,50,51,52,53,54,55,56,57,58,59,60^ were included in the review, and 21 studies^38,39,40,41,42,44,45,46,47,48,50,51,52,53,54,55,56,57,58,59,60^ were included in the meta-analysis (Figure 1). Almost perfect agreement on the study selection was achieved (97%; κ = 0.89).^61^

### Study Characteristics

Table 2 summarizes the characteristics of the included 23 RCTs published between 2002 and 2021. Most were conducted in the US (21 studies^38,40,41,42,43,44,46,47,48,49,50,51,52,53,54,55,56,57,58,59,60^), with 1 study each in Australia^45^ and Jamaica.^39^ Twenty-one studies^38,39,40,41,42,43,44,45,46,47,48,50,51,52,53,54,55,56,57,58,59^ used a 2-arm RCT design. In addition, most studies (12 [52%])^39,42,45,46,47,48,50,53,54,55,56,57^ reported that a control group received usual care.

**Table 2.** Characteristics of Included Studies in the Systematic Review

Source	Country	Child		Parent				IG approach, mode of delivery, and duration of delivery	CG	Psychosocial outcomes	Time points
		CHC (duration)	Age, mean (SD), y	Sample size (IG/CG), No.	Female sex, No. (%)	Male sex, No. (%)	Age, mean (SD), y				
Askins et al,^38^ 2009	US	Cancer (6 wk)	8.10 (NA)	197 (93/104)	197 (100)	0	36.3 (NA)	Online, individual, 8 wk	F2F PSST	Parents: depression, distress, PSS, and PTS	Pre/post; 3-mo follow up
Asnani et al,^39^ 2021	Jamaica	SCD (6-12 mo)	Range, 0.5-1	64 (32/32)	64 (100)	0	28.8 (5.9)	F2F, multifamily group, 6 mo	UC	Parents: depression, PSS, and parenting stress	Pre/post
Daniel et al,^40^ 2015	US	SCD (lifetime)	8.47 (2.11)	83 (42/41)	78 (94)	5 (6)	37.8 (NA)	F2F + online, multifamily group, 6 mo	WC	Parents: PSS; Children: QOL	Pre/post
DaWalt et al,^41^ 2018	US	ASD (8 y)	15.44 (1.03)	41 (16/25)	36 (88)	5 (12)	NA	F2F, multifamily group, 8 wk	WC	Parents: depression, PSS, and parenting stress; Children: social functioning; Family: parent-child conflict	Pre/post
Feinberg et al,^42^ 2014	US	ASD (5 mo)	2.83 (0.92)	120 (61/59)	120 (100)	0	33.5 (7.2)	F2F, individual, 8 wk	UC	Parents: depression, PSS, and parenting stress	Pre/post
Gerkensmeyer et al,^43^ 2013	US	Mental problem (1 y)	Range, 11-16	61 (30/31)	59 (97)	2 (3)	42.7 (9.2)	F2F + online, individual, 8 wk	WC	Parents: depression, PSS, and parenting stress	Pre/post; 3, 6-mo follow-up
Greenley et al,^44^ 2015	US	IBD (3.52 y)	14.5 (1.8)	76 (50/26)	71 (93)	5 (7)	NA	Online, parent-child,8 wk	WC	Children: QOL	Pre/Post
McCann et al,^45^ 2013	Australia	First-episode psychosis (2-3 y)	Range, 15-18	124 (61/63)	102 (82)	22 (18)	47.2 (8.3)	F2F + online, individual, 5 wk	UC	Parents: distress, parenting stress, and QOL	Pre/post; 4-mo follow-up
Modi et al,^46^ 2016	US	Epilepsy (<7 mo)	7.4 (3.4)	23 (11/12)	19 (83)	4 (17)	NA	F2F + online, parent-child, 8 wk	UC	Parents: PSS	Pre/post
Modi et al,^47^ 2021	US	Epilepsy (<8 mo)	7.7 (3.1)	56 (27/29)	55 (98)	1 (2)	NA	F2F + online, parent-child, 4 mo	UC	Children: QOL	Pre/post; 3, 6, 12-mo follow-up
Nansel et al,^48^ 2009	US	Type 1 diabetes (5.8 y)	11.5 (NA)	122 (60/62)	NA	NA	NA	F2F + online, parent-child, maximum of 12 mo	UC	Children: QOL; Family: parent-child conflict	Pre/post
Narad et al,^49^ 2019	US	TBI (5.8 mo)	14.9 (2.1)	101 (49/52)	87 (86)	14 (14)	NA	Online, parent-child, 6 mo	IRC	Parents: depression and distress; Family: family functioning and parent-child conflict	Pre/post
Palermo et al,^50^ 2016	US	Chronic pain (2 y)	14.3 (1.9)	61 (31/30)	60 (98)	1 (2)	45.7 (6.8)	F2F, individual, 6-8 wk	UC	Parents: depression, distress, PSS, PTS, parenting stress, QOL, and anxiety; Children: mental problems and social functioning	Pre/post; 3-mo follow-up
Petranovich et al,^51^ 2015	US	TBI (<6 mo)	14.9 (1.7)	132 (65/67)	119 (90)	13 (10)	42.8 (6.5)	Online, parent-child, 6 mo	IRC	Parents: depression and distress; Children: mental problems; Family: family functioning and parent-child conflict	Pre/post; 6, 12-mo follow-up
Phipps et al,^52^ 2020	US	Cancer (4-16 wk)	8.3 (5.5)	621 (310/311)	549 (88)	72 (12)	36.9 (8.7)	Online, individual, 8 wk	F2F PSST	Parents: depression, distress, PSS, and PTS	Pre/post; 3-mo follow-up
Sahler et al,^53^ 2002	US	Cancer (2-16 wk)	8.3 (5.5)	92 (50/42)	92 (100)	0	36.0 (6.7)	F2F, individual, 8 wk	UC	Parents: distress and PSS	Pre/post; 3-mo follow-up
Sahler et al,^54^ 2005	US	Cancer (2-16 wk)	7.6 (NA)	430 (217/213)	430 (100)	0	35.5 (NA)	F2F, individual, 8 wk	UC	Parents: depression, distress, PSS, and PTS	Pre/post; 6-mo follow-up
Sahler et al,^55^ 2013	US	Cancer (2-16 wk)	8.8 (5.9)	309 (157/152)	309 (100)	0	37.3 (8.3)	F2F, individual, 8 wk	UC	Parents: depression, distress, PSS, and PTS	Pre/post; 3-mo follow-up
Seid et al,^56^ 2010	US	Asthma (3.6 y)	7.3 (3.1)	171 (87/84)	165 (96)	6 (4)	NA	F2F, parent-child, 6 wk	UC	Children: QOL	Pre/post; 6-mo follow-up
Wade et al,^57^ 2006	US	TBI (8.8 mo)	10.8 (4.5)	32 (16/16)	24 (75)	8 (25)	NA	F2F, parent-child, 6 mo	UC	Parents: depression, distress, and anxiety; Children: mental problems; Family: parent-child conflict	Pre/post
Wade et al,^58^ 2006	US	TBI (13.7 mo)	10.8 (3.1)	40 (20/20)	36 (90)	4 (10)	NA	Online, parent-child, 6 mo	IRC	Parents: depression, distress, PSS, and anxiety; Children: mental problems and social functioning	Pre/post
Wade et al,^59^ 2012	US	TBI (9.6 mo)	14.3 (2.3)	35 (16/19)	NA	NA	41.2 (6.1)	Online, parent-child, 6 mo	IRC	Parents: depression, distress, and PSS; Children: mental problems	Pre/post
Wade et al,^60^ 2019	US	TBI (4.6 y)	16.5 (1.1)	150 (116/34)	127 (85)	23 (15)	NA	(1) Online, parent-child, 6 mo; (2) Online, parent-child, 6 mo	F2F PSST	Parents: depression and distress; Children: QOL and mental problems	Pre/post; 3-mo follow-up

Abbreviations: ASD, autism spectrum disorder; CHC, chronic health condition; CG, control group; F2F, face-to-face; IBD, inflammatory bowel disease; IG, intervention group; IRC, internet resource comparison (families were encouraged to spend 1 hour each week using the internet to access information); NA, not available; Pre/post, preintervention/postintervention; PSS, problem-solving skills; PSST, problem-solving skills training; PTS, posttraumatic stress; QOL, quality of life; SCD, sickle cell disease; TBI, traumatic brain injury; UC, usual care (including routine health maintenance for pediatric chronic health conditions [medication instruction and rehabilitation care], medical consultation [various presentations and complications], and supportive care [routine psychological care and health education]); WC, wait-list control.

A total of 3141 parents were included in this review. Twenty-one studies^38,39,40,41,42,43,44,45,46,47,49,50,51,52,53,54,55,56,57,58,60^ reported on parent sex, which totaled 2799 mothers (94%) and 185 fathers (6%), and 6 studies^38,39,42,53,54,55^ only recruited mothers. The age of the parents ranged from 20 to 67 years, with an estimated mean (SD) age of 38.3 (9.0) years. Of 2914 parents who reported race and ethnicity,^38,41,42,43,44,46,47,48,49,50,51,52,53,54,55,56,57,58,59,60^ 569 (19%) were Hispanic, 316 (11%) were non-Hispanic Black, 1708 (59%) were non-Hispanic White, and 321 (11%) were of other race or ethnicity. The CHC diagnoses were traumatic brain injury (6 studies),^49,51,57,58,59,60^ cancer (5 studies),^38,52,53,54,55^ sickle cell disease (2 studies),^39,40^ autism spectrum disorder (2 studies),^41,42^ epilepsy (2 studies),^46,47^ mental health problems (1 study),^43^ inflammatory bowel disease (1 study),^44^ first-episode psychosis (1 study),^45^ diabetes (1 study),^48^ chronic pain (1 study),^50^ and asthma (1 study).^56^ The mean (SD) age of the children was 10.0 (5.5) years, with the illness duration ranging from 2 weeks to 8 years.

Problem-solving skills training was confirmed as the primary focus of the intervention across the 23 RCTs, all of which were developed based on problem-solving therapy that emphasized positive problem orientation and covered the 5 core problem-solving steps (eTable 2 in Supplement 1). The number of PSST sessions included ranged from 2 to 21, with the duration of PSST varying from 5 weeks to 12 months. Most studies (18 [78%])^38,39,40,41,42,43,45,46,47,48,50,52,53,54,55,56,57,60^ involved interventions that required parents to attend face-to-face sessions, 6 of which integrated telephone-based online support.^40,43,45,46,47,48^ In the remaining studies,^38,44,49,51,52,58,59,60^ PSST was delivered entirely online, including via telephone sessions, web-based didactic modules, and videoconferences. Three interventions^39,40,41^ were group-based, 9 interventions^38,42,43,45,50,52,53,54,55^ were delivered to individuals 1 on 1, and 11 interventions^44,46,47,48,49,51,56,57,58,59,60^ included both parents and children.

### Risk of Bias

**Figure 2.**
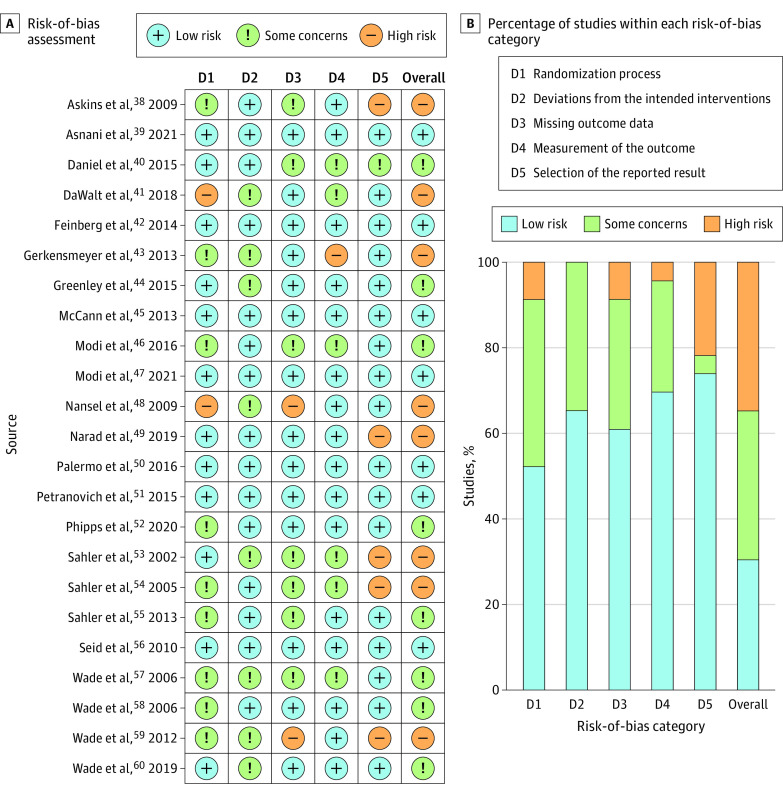
Risk-of-Bias Summary of the Included Studies

The methodological quality assessment resulted in 96% mutual agreement (κ = 0.93).^61^ Seven studies (30%)^39,42,45,47,50,51,56^ were classified as low risk, 8 studies (35%)^40,44,46,52,55,57,58,60^ raised some concerns, and 8 studies (35%)^38,41,43,48,49,53,54,59^ were identified as having a high risk (Figure 2). Two studies^41,48^ reported neither random sequence generation nor allocation concealment and hence were considered high risk for the randomization process. For 5 trials (22%),^38,49,53,54,59^ there was a high risk of reporting bias, as the prespecified outcomes were not fully reported (eTable 3 in Supplement 1).

### Meta-Analysis

**Figure 3.**
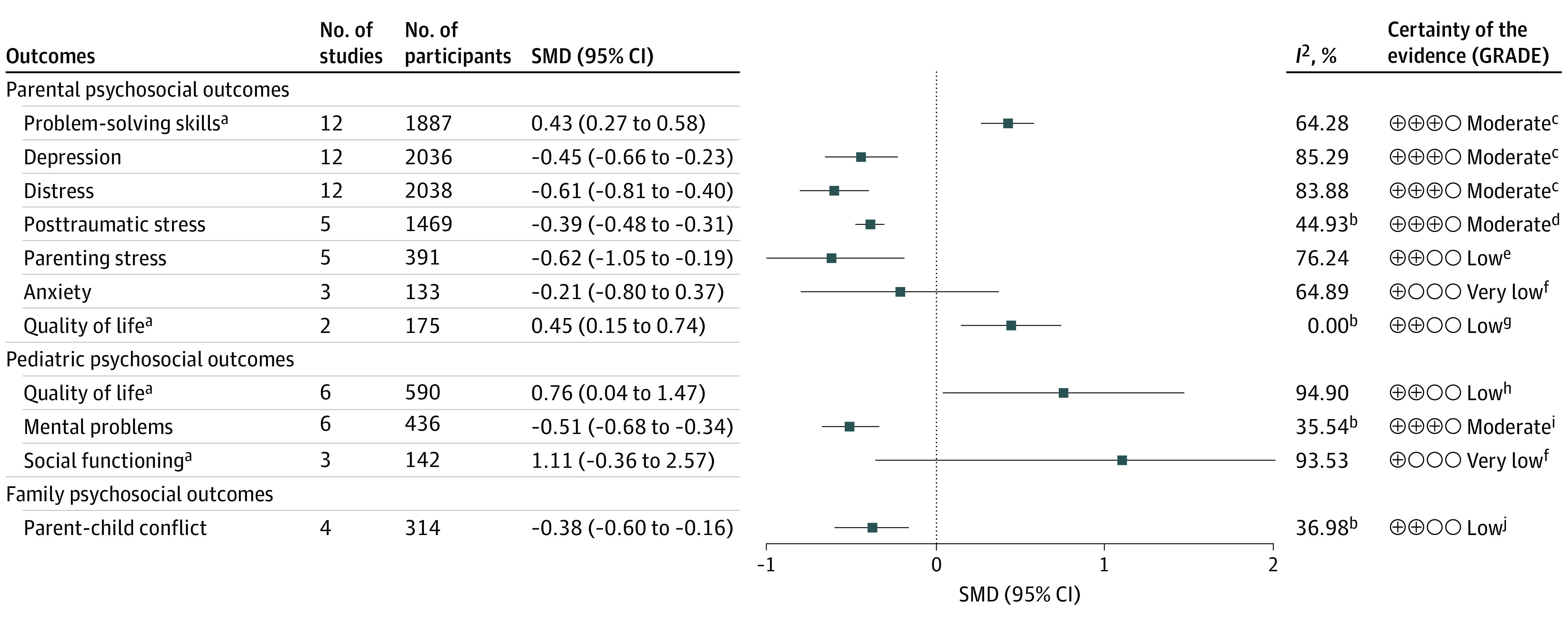
Meta-Analysis Summary of the Included Psychosocial Outcomes GRADE indicates Grading of Recommendations, Assessment, Development, and Evaluations; SMD, standardized mean difference. ^a^For meta-analysis of parental problem-solving skills, quality of life, pediatric quality of life, and social functioning, the problem-solving skills training (PSST) group was preferable when the effect size was greater than 0, while the value of effect size for other outcomes less than 0 indicated a favor of PSST. ^b^For meta-analysis of parental posttraumatic stress, quality of life, pediatric mental problems, and parent-child conflict, the values of *I*^2^ were less than 50%, and the inverse variance method was therefore used. ^c^Downgraded 1 level for serious inconsistency due to statistical heterogeneity. ^d^Downgraded 1 level for serious risk of bias of included studies. ^e^Downgraded 1 level for serious inconsistency due to statistical heterogeneity, and downgraded 1 level for serious imprecision due to limited sample size. ^f^Downgraded 1 level for serious inconsistency due to statistical heterogeneity, and downgraded 2 levels for very serious imprecision due to limited sample size and wide CIs. ^g^Downgraded 2 levels for very serious imprecision due to limited sample size and data from only 2 studies. ^h^Downgraded 1 level for serious inconsistency due to statistical heterogeneity and downgraded 1 level for serious imprecision due to wide CIs. ^i^Downgraded 1 level for serious imprecision due to limited sample size. ^j^Downgraded 1 level for serious risk of bias of included studies and downgraded 1 level for serious imprecision due to limited sample size.

Figure 3 illustrates the meta-analysis summary for all outcomes. Forest plots and GRADE ratings are presented in eFigure 1 and eTable 4 in Supplement 1, respectively.

#### Parental Outcomes

Overall, PSST had a significant positive effect on problem-solving skills (12 studies including 1887 parents^38,39,40,41,42,46,50,52,53,54,55,58^; SMD, 0.43; 95% CI, 0.27-0.58; *I*^2^ = 64.28%), depression (12 studies including 2036 parents^38,39,41,42,50,51,52,54,55,57,58,60^; SMD, −0.45; 95% CI, −0.66 to −0.23; *I*^2^ = 85.29%), and distress (12 studies including 2038 parents^38,45,50,51,52,53,54,55,57,58,59,60^; SMD, −0.61; 95% CI, −0.81 to −0.40; *I*^2^ = 83.88%), all of which indicated a medium effect size and moderate certainty evidence. The studies also showed that PSST significantly alleviated posttraumatic stress (5 studies including 1469 parents^38,50,52,54,55^; SMD, −0.39; 95% CI, −0.48 to −0.31; *I*^2^ = 44.93%) and parenting stress (5 studies including 391 parents^39,41,42,45,50^; SMD, −0.62; 95% CI, −1.05 to −0.19; *I*^2^ = 76.24%). The levels of evidence for the associations of PSST with lower posttraumatic stress and parenting stress were moderate and low, respectively. The meta-analysis of parental anxiety showed a positive but nonsignificant effect. In addition, 2 studies^45,50^ including 175 parents indicated a significant improvement in QOL among parents in the PSST group (SMD, 0.45; 95% CI, 0.15-0.74; *I*^2^ = 0.00%), with low-certainty evidence and no heterogeneity (Figure 3).

#### Pediatric and Family Outcomes

There was an association between PSST and better pediatric QOL compared with control groups (6 studies including 590 parents^40,44,47,48,56,60^; SMD, 0.76; 95% CI, 0.04-1.47; *I*^2^ = 94.90%). Data for 436 parents showed a significant association between PSST and fewer children’s mental problems (6 studies^50,51,57,58,59,60^; SMD −0.51; 95% CI, −0.68 to −0.34; *I*^2^ = 34.54%) (Figure 3). We found that PSST had both medium effect sizes for improving pediatric QOL and mental health, with low- and moderate-certainty evidence, respectively, whereas no association was found for social functioning. Four RCTs^41,48,51,57^ including 314 parents provided low-certainty evidence that PSST may reduce parent-child conflict (SMD, −0.38; 95% CI, −0.60 to −0.16), with moderate heterogeneity (*I^2^* = 36.98%).

### Subgroup Analysis

Subgroup analyses were conducted according to child- and intervention-level characteristics (eFigure 2 in Supplement 1). Subgroup analysis by child age indicated that PSST was associated with significant changes in parental depression (SMD, −0.39; 95% CI, −0.52 to −0.26), problem-solving skills (SMD, 0.36; 95% CI, 0.24-0.48), posttraumatic stress (SMD, −0.40; 95% CI, −0.49 to −0.31), and parenting stress (SMD, −0.43; 95% CI, −0.72 to −0.13) for the parents of children who were 10 years or younger compared with the parents of older children (>10 years). Regarding changes in parental depression and posttraumatic stress, PSST had no association for parents of children who had not been newly diagnosed with a CHC but was associated with significant changes (reductions) for parents of children with newly diagnosed CHCs (depression: SMD, −0.40 [95% CI, −0.52 to −0.28]; posttraumatic stress: SMD, −0.40 [95% CI, −0.49 to −0.31]). Furthermore, compared with other medical conditions, PSST was associated with significant improvement in all psychosocial outcomes in parents of children diagnosed with cancer.

Overall, PSST delivered online yielded larger effects on all outcomes except for parent-child conflict than only face-to-face PSST. There was a significant improvement in depression (SMD, −0.39; 95% CI, −0.52 to −0.27) and problem-solving skills (SMD, 0.37; 95% CI, 0.24-0.50) among parents who received individual-based PSST. However, the parent-child interventions showed significant changes in pediatric and family psychosocial outcomes. As for intervention duration, PSST for 5 to 8 weeks had stronger effects on reducing parental depression and parenting stress and improving problem-solving skills than PSST with durations exceeding 8 weeks. The number of sessions followed a similar pattern, with significant improvements in depression (SMD, −0.48; 95% CI, −0.67 to −0.28) and problem-solving skills (SMD, 0.50; 95% CI, 0.29-0.70) among parents who underwent 8 to 12 sessions.

### Publication Bias and Sensitivity Analyses

We assessed the publication bias for outcomes that included more than 10 trials (problem-solving skills, parental depression, and distress). Overall, the funnel plots were mostly symmetrical (eFigure 3 in Supplement 1); Egger tests were not significant for problem-solving skills (*z* = 1.64, *P* = .10), depression (*z* = −1.21, *P* = .23), and distress (*z* = −0.46, *P* = .65), thus indicating no publication bias. The leave-one-out sensitivity analyses yielded similar results to those of the primary analyses, indicating the robustness of key outcomes (eFigure 4 in Supplement 1).

## Discussion

This systematic review and meta-analysis of 23 RCTs is the first to our knowledge to adequately examine the positive association of PSST with improved parental, pediatric, and family psychosocial outcomes. The findings show that PSST was associated with improved problem-solving skills, less negative affectivity, and better QOL for parents. Positivity and problem-solving throughout PSST is achieved by refining problems and effectively troubleshooting obstacles commonly encountered during the treatment of childhood CHCs, thus contributing to parental well-being.^15,41^ Additionally, PSST was associated with improvements in pediatric QOL, mental health, and parent-child conflict, in accordance with previous review results that psychological interventions for parents may facilitate their ability to scaffold behavioral and emotional changes in their children and thus reduce conflicts between parents and children.^19,27,62^ Our findings extend this evidence by suggesting that PSST is also associated with better psychosocial outcomes for children and families, showing promise for the use of PSST to increase the well-being of all family members and promote family adaptation.

Problem-solving skills training is an emerging and promising area of research, with 17 (74%) included studies published in 2010 or later.^39,40,41,42,43,44,45,46,47,49,50,51,52,55,56,59,60^ A total of 3141 patients were included in this review, and there were sufficient sample sizes for most outcomes. Although the included RCTs were conducted in only 3 countries, which may decrease the representativeness of the results in terms of dissemination capability, the ethnic and linguistic diversity of parents across included studies showed equally positive responses to PSST when presented in various contexts. Across all psychosocial outcomes, the certainty of the evidence varied from moderate to very low. Despite the suggested effectiveness of PSST in this review, some heterogeneity remains. On one hand, the included studies used diverse definitions and instruments to measure psychosocial outcomes; on the other hand, the studies included parents of children with 11 different CHCs, all of which may have introduced clinical heterogeneity. However, the diversity may also suggest a better clinical fitness of the evidence in this review. Additionally, the current evidence on the long-term effects of PSST is limited by the small number of follow-up studies. Overall, although our review provides relatively high certainty of evidence, further research on higher-level evidence with sustained follow-up is warranted. Furthermore, it is necessary to expand the range of outcomes (eg, physical and clinical) to fully reflect the effectiveness of PSST, as most relevant studies have only reported psychosocial outcomes.

Our subgroup analysis revealed a significant decrease in negative affectivity among the parents of children aged 10 years or younger and who had been newly diagnosed with CHCs, as younger children are more reliant on their parents for daily life and disease management.^63^ These findings are compatible with broader evidence supporting early PSST’s improvement of parental well-being when children are newly diagnosed.^64^ A significant decrease in negative emotions was also found among parents of children with cancer compared with the parents of children with other medical conditions, possibly because cancer is a leading cause of death in children^65^ and their parents may experience a substantial care burden.^8,66^ The subgroup analysis according to intervention characteristics indicated that online intervention yielded larger effects on most outcomes than the in-person approach, which may be due to the flexibility and wider dissemination of an online approach.^52,67^ With the rapid development of internet and mobile technologies in pediatric nursing,^68^ future research could combine in-person PSST with enhanced online materials. Additionally, individual-based PSST was preferable for parental well-being, whereas the parent-child intervention favored pediatric and family psychosocial outcomes. The participants had more opportunities to receive personalized feedback in the individual-based interventions that included 1-on-1 activities^69^ and to enhance family communication and cohesion in the parent-child intervention.^26^ Hence, it may be worthwhile to integrate parent-child interaction when tailoring PSST according to families’ needs. Finally, PSST delivered for 5 to 8 weeks and consisting of 8 to 12 sessions had stronger associations in terms of parental psychosocial outcomes. This finding highlights the importance of shorter periods and less complexity to higher engagement in PSST, as parents’ busy schedules may interfere with long-term interventions.^70^

### Limitations

This review had several limitations. First, we limited our search to articles in English and Chinese, which might have led to selection bias and affected the reliability of the results. Second, some of the findings must be interpreted with caution, as they were based on only 2 or 3 studies. Third, the assessment could differ across people due to the methodological subjectivity of the risk-of-bias tool and GRADE. Fourth, the psychosocial outcomes identified in this review were measured using multiple scales, and despite using SMD as recommended, the heterogeneity of most outcomes was high. Hence, the interpretability and application of the results were diminished. Finally, only the postintervention data were analyzed, as follow-up data were not reported consistently and sufficiently, and the long-term outcomes remain unclear.

## Conclusions

The findings of this systematic review and meta-analysis suggest that PSST is associated with improvements in parental psychosocial outcomes (problem-solving skills, depression, distress, posttraumatic stress, parenting stress, and QOL) as well as pediatric (QOL and mental problems) and family psychosocial outcomes (parent-child conflict). Moreover, our findings on children- and intervention-level characteristics may guide the design and delivery of future PSST by presenting information on factors associated with effectiveness. Further high-quality RCTs with longer follow-up times and that explore physical and clinical outcomes are encouraged to generate adequate evidence for PSST. In conclusion, PSST should be an active component of psychosocial interventions for parents of children with CHCs.

## Notes

Supplement 1. **eTable 1.** Study Search Strategies **eTable 2.** Intervention Characteristics of Included Studies **eTable 3.** Author Judgments of Risk of Bias Across All Included Studies **eTable 4.** Grading of Recommendations Assessment, Development and Evaluation (GRADE) Evidence Profile **eFigure 1.** Forest Plot Meta-Analyses for Different Psychosocial Outcomes **eFigure 2.** Subgroup Analyses of Each Outcome According to Children- and Intervention-Level Factors **eFigure 3.** Funnel Plot Analyses **eFigure 4.** Leave-One-Out Sensitivity Analyses

Supplement 2.
Data Sharing Statement

## References

[1] van der Lee JH, Mokkink LB, Grootenhuis MA, Heymans HS, Offringa M. Definitions and measurement of chronic health conditions in childhood: a systematic review. JAMA. 2007;297(24):2741-2751. doi:10.1001/jama.297.24.2741 17595275

[2] 2020-2021 National Survey of Children’s Health (NSCH) data query. Data Resource Center for Child and Adolescent Health; Accessed July 2, 2023. Child and Adolescent Health Measurement Initiative. http://www.childhealthdata.org

[3] Luo Y, Li HCW, Xia W, Cheung AT, Ho LLK, Chung JOK. The lived experience of resilience in parents of children with cancer: a phenomenological study. Front Pediatr. 2022;10:871435. doi:10.3389/fped.2022.871435 35707743 PMC9189362

[4] Mu PF, Lee MY, Sheng CC, Tung PC, Huang LY, Chen YW. The experiences of family members in the year following the diagnosis of a child or adolescent with cancer: a qualitative systematic review. JBI Database System Rev Implement Rep. 2015;13(5):293-329. doi:10.11124/01938924-201513050-00015 26455612

[5] Smith S, Tallon M, Clark C, Jones L, Mörelius E. “You never exhale fully because you’re not sure what’s next”: parents’ experiences of stress caring for children with chronic conditions. Front Pediatr. 2022;10:902655. doi:10.3389/fped.2022.902655 35832577 PMC9271768

[6] Cohn LN, Pechlivanoglou P, Lee Y, . Health outcomes of parents of children with chronic illness: a systematic review and meta-analysis. J Pediatr. 2020;218:166-177.e2. doi:10.1016/j.jpeds.2019.10.068 31916997

[7] Landolt MA, Ystrom E, Sennhauser FH, Gnehm HE, Vollrath ME. The mutual prospective influence of child and parental post-traumatic stress symptoms in pediatric patients. J Child Psychol Psychiatry. 2012;53(7):767-774. doi:10.1111/j.1469-7610.2011.02520.x 22211718

[8] van Warmerdam J, Zabih V, Kurdyak P, Sutradhar R, Nathan PC, Gupta S. Prevalence of anxiety, depression, and posttraumatic stress disorder in parents of children with cancer: a meta-analysis. Pediatr Blood Cancer. 2019;66(6):e27677. doi:10.1002/pbc.27677 30816008

[9] Pinquart M. Parenting stress in caregivers of children with chronic physical condition—a meta-analysis. Stress Health. 2018;34(2):197-207. doi:10.1002/smi.2780 28834111

[10] Cousino MK, Hazen RA. Parenting stress among caregivers of children with chronic illness: a systematic review. J Pediatr Psychol. 2013;38(8):809-828. doi:10.1093/jpepsy/jst049 23843630

[11] Klassen AF, Klaassen R, Dix D, . Impact of caring for a child with cancer on parents’ health-related quality of life. J Clin Oncol. 2008;26(36):5884-5889. doi:10.1200/JCO.2007.15.2835 19029424

[12] Calatrava M, Martins MV, Schweer-Collins M, Duch-Ceballos C, Rodríguez-González M. Differentiation of self: a scoping review of Bowen Family Systems Theory’s core construct. Clin Psychol Rev. 2022;91:102101. doi:10.1016/j.cpr.2021.102101 34823190

[13] Van Schoors M, De Mol J, Morren H, Verhofstadt LL, Goubert L, Van Parys H. Parents’ perspectives of changes within the family functioning after a pediatric cancer diagnosis: a multi family member interview analysis. Qual Health Res. 2018;28(8):1229-1241. doi:10.1177/1049732317753587 29357749

[14] Bakula DM, Sharkey CM, Perez MN, . Featured article: the relationship between parent and child distress in pediatric cancer: a meta-analysis. J Pediatr Psychol. 2019;44(10):1121-1136. doi:10.1093/jpepsy/jsz051 31260071

[15] Dolgin MJ, Devine KA, Tzur-Bitan D, . Responsivity to problem-solving skills training in mothers of children with cancer. J Pediatr Psychol. 2021;46(4):413-421. doi:10.1093/jpepsy/jsaa117 33367833

[16] Mao S, Lu H, Zhang Y, . Evaluation of psychosocial pathways to family adaptation of Chinese patients with liver cancer using the McCubbin’s Family Resilience Model. Front Psychiatry. 2021;12:703137. doi:10.3389/fpsyt.2021.703137 34975555 PMC8717998

[17] Tan R, Koh S, Wong ME, Rui M, Shorey S. Caregiver stress, coping strategies, and support needs of mothers caring for their children who are undergoing active cancer treatments. Clin Nurs Res. 2020;29(7):460-468. doi:10.1177/1054773819888099 31709812

[18] Gurtovenko K, Fladeboe KM, Galtieri LR, . Stress and psychological adjustment in caregivers of children with cancer. Health Psychol. 2021;40(5):295-304. doi:10.1037/hea0001070 34152783 PMC9053835

[19] Law EF, Fisher E, Fales J, Noel M, Eccleston C. Systematic review and meta-analysis of parent and family-based interventions for children and adolescents with chronic medical conditions. J Pediatr Psychol. 2014;39(8):866-886. doi:10.1093/jpepsy/jsu032 24881048 PMC6296404

[20] D’Zurilla TJ, Goldfried MR. Problem solving and behavior modification. J Abnorm Psychol. 1971;78(1):107-126. doi:10.1037/h0031360 4938262

[21] D’Zurilla TJ, Nezu AM, Maydeu-Olivares A. Social problem solving: theory and assessment. In: Chang EC, D’Zurilla TJ, Sanna LJ, eds. Social Problem Solving: Theory, Research, and Training. American Psychological Association; 2004:11-27. doi:10.1037/10805-001

[22] Nezu AM, Nezu CM, Felgoise SH, McClure KS, Houts PS. Project Genesis: assessing the efficacy of problem-solving therapy for distressed adult cancer patients. J Consult Clin Psychol. 2003;71(6):1036-1048. doi:10.1037/0022-006X.71.6.1036 14622079

[23] Rivera PA, Elliott TR, Berry JW, Grant JS. Problem-solving training for family caregivers of persons with traumatic brain injuries: a randomized controlled trial. Arch Phys Med Rehabil. 2008;89(5):931-941. doi:10.1016/j.apmr.2007.12.032 18452743 PMC2518069

[24] Voll M, Fairclough DL, Morrato EH, . Dissemination of an evidence-based behavioral intervention to alleviate distress in caregivers of children recently diagnosed with cancer: Bright IDEAS. Pediatr Blood Cancer. 2022;69(10):e29904. doi:10.1002/pbc.29904 35929012 PMC9420785

[25] Eccleston C, Fisher E, Law E, Bartlett J, Palermo TM. Psychological interventions for parents of children and adolescents with chronic illness. Cochrane Database Syst Rev. 2015;4(4):CD009660. doi:10.1002/14651858.CD009660.pub3 25874881 PMC4838404

[26] Koumarianou A, Symeonidi AE, Kattamis A, Linardatou K, Chrousos GP, Darviri C. A review of psychosocial interventions targeting families of children with cancer. Palliat Support Care. 2021;19(1):103-118. doi:10.1017/S1478951520000449 32613930

[27] Law E, Fisher E, Eccleston C, Palermo TM. Psychological interventions for parents of children and adolescents with chronic illness. Cochrane Database Syst Rev. 2019;3(3):CD009660. 30883665 10.1002/14651858.CD009660.pub4 PMC6450193

[28] Masulani-Mwale C, Mathanga D, Kauye F, Gladstone M. Psychosocial interventions for parents of children with intellectual disabilities—a narrative review and implications for low income settings. Ment Health Prev. 2018;11:24-32. doi:10.1016/j.mhp.2018.05.003

[29] Page MJ, McKenzie JE, Bossuyt PM, . The PRISMA 2020 statement: an updated guideline for reporting systematic reviews. BMJ. 2021;372(71):n71. doi:10.1136/bmj.n71 33782057 PMC8005924

[30] Ouzzani M, Hammady H, Fedorowicz Z, Elmagarmid A. Rayyan—a web and mobile app for systematic reviews. Syst Rev. 2016;5(1):210. doi:10.1186/s13643-016-0384-4 27919275 PMC5139140

[31] Sterne JAC, Savović J, Page MJ, . RoB 2: a revised tool for assessing risk of bias in randomised trials. BMJ. 2019;366:l4898. doi:10.1136/bmj.l4898 31462531

[32] Guyatt GH, Oxman AD, Vist GE, ; GRADE Working Group. GRADE: an emerging consensus on rating quality of evidence and strength of recommendations. BMJ. 2008;336(7650):924-926. doi:10.1136/bmj.39489.470347.AD 18436948 PMC2335261

[33] Balshem H, Helfand M, Schünemann HJ, . GRADE guidelines: 3. Rating the quality of evidence. J Clin Epidemiol. 2011;64(4):401-406. doi:10.1016/j.jclinepi.2010.07.015 21208779

[34] Atkins D, Best D, Briss PA, ; GRADE Working Group. Grading quality of evidence and strength of recommendations. BMJ. 2004;328(7454):1490. doi:10.1136/bmj.328.7454.1490 15205295 PMC428525

[35] Cohen J. A power primer. Psychol Bull. 1992;112(1):155-159. doi:10.1037/0033-2909.112.1.155 19565683

[36] Higgins JPT, Thompson SG, Deeks JJ, Altman DG. Measuring inconsistency in meta-analyses. BMJ. 2003;327(7414):557-560. doi:10.1136/bmj.327.7414.557 12958120 PMC192859

[37] Egger M, Davey Smith G, Schneider M, Minder C. Bias in meta-analysis detected by a simple, graphical test. BMJ. 1997;315(7109):629-634. doi:10.1136/bmj.315.7109.629 9310563 PMC2127453

[38] Askins MA, Sahler OJ, Sherman SA, . Report from a multi-institutional randomized clinical trial examining computer-assisted problem-solving skills training for English- and Spanish-speaking mothers of children with newly diagnosed cancer. J Pediatr Psychol. 2009;34(5):551-563. doi:10.1093/jpepsy/jsn124 19091804 PMC2684487

[39] Asnani MR, Francis D, Knight-Madden J, Chang-Lopez S, King L, Walker S. Integrating a problem-solving intervention with routine care to improve psychosocial functioning among mothers of children with sickle cell disease: a randomized controlled trial. PLoS One. 2021;16(6):e0252513. doi:10.1371/journal.pone.0252513 34106974 PMC8189456

[40] Daniel LC, Li Y, Smith K, . Lessons learned from a randomized controlled trial of a family-based intervention to promote school functioning for school-age children with sickle cell disease. J Pediatr Psychol. 2015;40(10):1085-1094. doi:10.1093/jpepsy/jsv063 26136404 PMC4626743

[41] DaWalt LS, Greenberg JS, Mailick MR. Transitioning together: a multi-family group psychoeducation program for adolescents with ASD and their parents. J Autism Dev Disord. 2018;48(1):251-263. doi:10.1007/s10803-017-3307-x 29032481 PMC5762411

[42] Feinberg E, Augustyn M, Fitzgerald E, . Improving maternal mental health after a child’s diagnosis of autism spectrum disorder: results from a randomized clinical trial. JAMA Pediatr. 2014;168(1):40-46. doi:10.1001/jamapediatrics.2013.3445 24217336

[43] Gerkensmeyer JE, Johnson CS, Scott EL, . Problem-solving intervention for caregivers of children with mental health problems. Arch Psychiatr Nurs. 2013;27(3):112-120. doi:10.1016/j.apnu.2013.01.004 23706887 PMC3697759

[44] Greenley RN, Gumidyala AP, Nguyen E, . Can you teach a teen new tricks? problem solving skills training improves oral medication adherence in pediatric patients with inflammatory bowel disease participating in a randomized trial. Inflamm Bowel Dis. 2015;21(11):2649-2657. doi:10.1097/MIB.0000000000000530 26218142

[45] McCann TV, Lubman DI, Cotton SM, . A randomized controlled trial of bibliotherapy for carers of young people with first-episode psychosis. Schizophr Bull. 2013;39(6):1307-1317. doi:10.1093/schbul/sbs121 23172001 PMC3796072

[46] Modi AC, Guilfoyle SM, Mann KA, Rausch JR. A pilot randomized controlled clinical trial to improve antiepileptic drug adherence in young children with epilepsy. Epilepsia. 2016;57(3):e69-e75. doi:10.1111/epi.13289 26693964 PMC4783218

[47] Modi AC, Guilfoyle SM, Glauser TA, Mara CA. Supporting treatment adherence regimens in children with epilepsy: a randomized clinical trial. Epilepsia. 2021;62(7):1643-1655. doi:10.1111/epi.16921 33982280 PMC8647767

[48] Nansel TR, Anderson BJ, Laffel LM, . A multisite trial of a clinic-integrated intervention for promoting family management of pediatric type 1 diabetes: feasibility and design. Pediatr Diabetes. 2009;10(2):105-115. doi:10.1111/j.1399-5448.2008.00448.x 18721167 PMC2843426

[49] Narad ME, Raj S, Yeates KO, . Randomized controlled trial of an online problem-solving intervention following adolescent traumatic brain injury: family outcomes. Arch Phys Med Rehabil. 2019;100(5):811-820. doi:10.1016/j.apmr.2019.01.010 30738021 PMC11047263

[50] Palermo TM, Law EF, Bromberg M, Fales J, Eccleston C, Wilson AC. Problem-solving skills training for parents of children with chronic pain: a pilot randomized controlled trial. Pain. 2016;157(6):1213-1223. doi:10.1097/j.pain.0000000000000508 26845525 PMC4935529

[51] Petranovich CL, Wade SL, Taylor HG, . Long-term caregiver mental health outcomes following a predominately online intervention for adolescents with complicated mild to severe traumatic brain injury. J Pediatr Psychol. 2015;40(7):680-688. doi:10.1093/jpepsy/jsv001 25682211 PMC4505072

[52] Phipps S, Fairclough DL, Noll RB, . In-person vs. web-based administration of a problem-solving skills intervention for parents of children with cancer: report of a randomized noninferiority trial. EClinicalMedicine. 2020;24:100428. doi:10.1016/j.eclinm.2020.100428 32637901 PMC7327899

[53] Sahler OJ, Varni JW, Fairclough DL, . Problem-solving skills training for mothers of children with newly diagnosed cancer: a randomized trial. J Dev Behav Pediatr. 2002;23(2):77-86. doi:10.1097/00004703-200204000-00003 11943969

[54] Sahler OJ, Fairclough DL, Phipps S, . Using problem-solving skills training to reduce negative affectivity in mothers of children with newly diagnosed cancer: report of a multisite randomized trial. J Consult Clin Psychol. 2005;73(2):272-283. doi:10.1037/0022-006X.73.2.272 15796635

[55] Sahler OJ, Dolgin MJ, Phipps S, . Specificity of problem-solving skills training in mothers of children newly diagnosed with cancer: results of a multisite randomized clinical trial. J Clin Oncol. 2013;31(10):1329-1335. doi:10.1200/JCO.2011.39.1870 23358975 PMC3607672

[56] Seid M, Varni JW, Gidwani P, Gelhard LR, Slymen DJ. Problem-solving skills training for vulnerable families of children with persistent asthma: report of a randomized trial on health-related quality of life outcomes. J Pediatr Psychol. 2010;35(10):1133-1143. doi:10.1093/jpepsy/jsp133 20061311 PMC7714040

[57] Wade SL, Michaud L, Brown TM. Putting the pieces together: preliminary efficacy of a family problem-solving intervention for children with traumatic brain injury. J Head Trauma Rehabil. 2006;21(1):57-67. doi:10.1097/00001199-200601000-00006 16456392

[58] Wade SL, Carey J, Wolfe CR. An online family intervention to reduce parental distress following pediatric brain injury. J Consult Clin Psychol. 2006;74(3):445-454. doi:10.1037/0022-006X.74.3.445 16822102

[59] Wade SL, Walz NC, Carey J, . A randomized trial of teen online problem solving: efficacy in improving caregiver outcomes after brain injury. Health Psychol. 2012;31(6):767-776. doi:10.1037/a0028440 22746261

[60] Wade SL, Cassedy AE, McNally KA, . A randomized comparative effectiveness trial of family-problem-solving treatment for adolescent brain injury: parent outcomes from the Coping with Head Injury through Problem Solving (CHIPS) study. J Head Trauma Rehabil. 2019;34(6):E1-E9. doi:10.1097/HTR.0000000000000487 31033747

[61] Schober P, Mascha EJ, Vetter TR. Statistics from A (agreement) to Z (*z* score): a guide to interpreting common measures of association, agreement, diagnostic accuracy, effect size, heterogeneity, and reliability in medical research. Anesth Analg. 2021;133(6):1633-1641. doi:10.1213/ANE.0000000000005773 34633993

[62] Park M, Choi EK, Lee HJ, Park HE, Chinbayar A. Resilience-promoting programs in families of children with cancer: a systematic review. J Pediatr Hematol Oncol Nurs. 2022;39(3):185-201. doi:10.1177/27527530211055997 35467437

[63] Bradford NK, Bowers A, Chan RJ, . Documentation of symptoms in children newly diagnosed with cancer highlights the need for routine assessment using self-report. Cancer Nurs. 2021;44(6):443-452. doi:10.1097/NCC.0000000000000849 34694084

[64] Peek G, Melnyk BM. Coping interventions for parents of children newly diagnosed with cancer: an evidence review with implications for clinical practice and future research. Pediatr Nurs. 2010;36(6):306-313. 21291047

[65] Sung H, Ferlay J, Siegel RL, . Global cancer statistics 2020: GLOBOCAN estimates of incidence and mortality worldwide for 36 cancers in 185 countries. CA Cancer J Clin. 2021;71(3):209-249. doi:10.3322/caac.21660 33538338

[66] Shokri M, Tarjoman A, Borji M, Solaimanizadeh L. Investigating psychological problems in caregiver of pediatrics with cancer: a systematic review. J Child Adolesc Psychiatr Nurs. 2020;33(4):229-238. doi:10.1111/jcap.12269 32275101

[67] Chambers SK, Ritterband LM, Thorndike F, . Web-delivered cognitive behavioral therapy for distressed cancer patients: randomized controlled trial. J Med Internet Res. 2018;20(1):e42. doi:10.2196/jmir.8850 29386173 PMC5812983

[68] Wolfe J, Orellana L, Cook EF, . Improving the care of children with advanced cancer by using an electronic patient-reported feedback intervention: results from the PediQUEST randomized controlled trial. J Clin Oncol. 2014;32(11):1119-1126. doi:10.1200/JCO.2013.51.5981 24616307 PMC3970170

[69] Rosenberg AR, Bradford MC, Junkins CC, . Effect of the Promoting Resilience in Stress Management Intervention for Parents of Children With Cancer (PRISM-P): a randomized clinical trial. JAMA Netw Open. 2019;2(9):e1911578. doi:10.1001/jamanetworkopen.2019.11578 31532518 PMC6751761

[70] Kirkham JG, Seitz DP. More evidence for problem-solving therapy: improving access is still a problem in need of solving. Int Psychogeriatr. 2022;34(2):105-107. doi:10.1017/S1041610221000077 34044902

