# Supplementary material for: Problem-Solving Skills Training for Parents of Children With Chronic Health Conditions: A Systematic Review and Meta-Analysis
Source: JAMA Pediatr. 2024 Jan 2;178(3):226–36. doi: 10.1001/jamapediatrics.2023.5753 (PMC10762633; doi:10.1001/jamapediatrics.2023.5753)
Supplement: Supplement 1. — eTable 1. Study Search Strategies eTable 2. Intervention Characteristics of Included Studies eTable 3. Author Judgments of Risk of Bias Across All Included Studies eTable 4. Grading of Recommendations Assessment, Development and Evaluation (GRADE) Evidence Profile eFigure 1. Forest Plot Meta-Analyses for Different Psychosocial Outcomes eFigure 2. Subgroup Analyses of Each Outcome According to Children- and Intervention-Level Factors eFigure 3. Funnel Plot Analyses eFigure 4. Leave-One-Out Sensitivity Analyses [file jamapediatr-e235753-s001.pdf]

## Supplemental Online Content

Zhou T, Luo Y, Xiong W, Meng Z, Zhang H, Zhang J. Problem-solving skills training for parents of children with chronic health conditions. *JAMA Pediatr*. Published online January 2, 2024. doi:10.1001/jamapediatrics.2023.5753

**eTable 1.** Study Search Strategies

**eTable 2.** Intervention Characteristics of Included Studies

**eTable 3.** Author Judgments of Risk of Bias Across All Included Studies

**eTable 4.** Grading of Recommendations Assessment, Development, and Evaluation (GRADE) Evidence Profile

**eFigure 1.** Forest Plot Meta-Analyses for Different Psychosocial Outcomes

**eFigure 2.** Subgroup Analyses of Each Outcome According to Children- and Intervention-Level Factors

**eFigure 3.** Funnel Plot Analyses

**eFigure 4.** Leave-One-Out Sensitivity Analyses

This supplemental material has been provided by the authors to give readers additional information about their work.

© 2024 American Medical Association. All rights reserved.

**eTable 1. Study Search Strategies**

**PubMed**
Search date: 30/04/2023
Number of results: 523

| No. | Search strategies |
|---|---|
| #1 | "Child" [Mesh] OR "Pediatrics" [Mesh] OR "Adolescent" [Mesh] OR "Infant" [Mesh] OR "Minors" [Mesh] |
| #2 | "child*" OR "kid*" OR "boy*" OR "girl*" OR "preschool*" OR "schoolchild" OR "pediatric*" OR "paediatric*" OR "adolescen*" OR "teen*" OR "preteen*" OR "youth*" OR "juvenil*" OR "young" OR "puber" OR "pubescen*" OR "underage*" OR "under-age*" OR "infan*" OR "neonat*" OR "neo-nat*" OR "newborn*" OR "new-born*" OR "baby" OR "babies" OR "toddler*" OR "minor*" OR "offspring*" OR "young people" |
| #3 | #1 OR #2 |
| #4 | "Chronic Disease" [Mesh] OR "Anemia" [Mesh] OR "Arthritis" [Mesh] OR "Asthma" [Mesh] OR "Attention Deficit Disorder with Hyperactivity" [Mesh] OR "Autism Spectrum Disorder" [Mesh] OR "Asperger Syndrome" [Mesh] OR "Autistic Disorder" [Mesh] OR "Vision Disorders" [Mesh] OR "Brain Injuries" [Mesh] OR "Bronchitis" [Mesh] OR "Cerebral Palsy" [Mesh] OR "Congenital Abnormalities" [Mesh] OR "Cystic Fibrosis" [Mesh] OR "Developmental Disabilities" [Mesh] OR "Diabetes Mellitus" [Mesh] OR "Down Syndrome" [Mesh] OR "Epilepsy" [Mesh] OR "Genetic Diseases, Inborn" [Mesh] OR "Heart Defects, Congenital" [Mesh] OR "Heart Diseases" [Mesh] OR "Hematologic Diseases" [Mesh] OR "Anemia, Sickle Cell" [Mesh] OR "Hemophilia A" [Mesh] OR "HIV" [Mesh] OR "Intellectual Disability" [Mesh] OR "Kidney Diseases" [Mesh] OR "Mental Disorders" [Mesh] OR "Musculoskeletal Diseases" [Mesh] OR "Neoplasms" [Mesh] OR "Schizophrenia" [Mesh] OR "Spinal Dysraphism" [Mesh] OR "Thalassemia" [Mesh] OR "Trisomy" [Mesh] |
| #5 | "chronic disease*" OR "chronic ill*" OR "chronically ill*" OR "chronic disorder*" OR "chronic health" OR "chronic condition*" OR "anemia*" OR "arthriti*" OR "osteoarthriti*" OR "polyarthriti*" OR "asthma*" OR "ADHD" OR "attention deficit disorder*" OR "autism" OR "autistic" OR "asperger disease*" OR "asperger disorders" OR "asperger syndrome" OR "kanner* syndrome" OR "ASD" OR "blindness" OR "amaurosis" OR "vision loss*" OR "loss of vision" OR "vision disorder*" OR "visual* impair*" OR "brain injur*" OR "brain laceration*" OR "TBI" OR "TBIS" OR "bronchiti*" OR "cerebral pals*" OR "little* disease" OR "spastic diplegia*" OR "birth defect*" OR "congenital abnormalit*" OR "congenital anomalies" OR "congenital defect*" OR "deformit*" OR "cystic fibrosis" OR "fibrocystic disease of pancreas" OR "mucoviscidosis" OR "pancreas fibrocystic disease*" OR "development* deviation*" OR "development* disab*" OR "development* disorder*" OR "developmental delay disorder*" OR "diabetes" OR "down* syndrome" OR "mongolism" OR "trisomy 21" OR "trisomy g" OR "epilep*" OR "seizure disorder*" OR "genetic disease*" OR "genetic disorder*" OR "hereditary disease*" OR "single gene defects" OR "cardiac disease*" OR "cardiac disorder*" OR "heart disease*" OR |

© 2024 American Medical Association. All rights reserved.

| | |
|---|---|
| | “heart disorder*” OR “heart defect*” OR “heart abnormalit*” OR “heart malform*” OR “blood disease*” OR “blood disorder*” OR “haematologic* disease*” OR “hematologic* disease*” OR “haematologic* disorder*” OR “hematologic* disorder*” OR “sickle cell anemia*” OR “sickle cell disease*” OR “sickle cell disorder*” OR “hbs disease” OR “hemoglobin s disease” OR “haemophilia*” OR “hemophilia*” OR “factor viii deficien*” OR “factor 8 deficien*” OR “aids virus*” OR “acquired immune deficiency syndrome virus*” OR “AIDS virus*” OR “hiv” OR “htlv-iii” OR “HIV*” OR “human t cell leukemia virus type iii” OR “human t cell lymphotropic virus type iii” OR “human t lymphotropic virus type iii” OR “lav-htlv-iii” OR “lymphadenopathy associated virus*” OR “intellectual development disorder*” OR “intellectual disabilit*” OR “mental deficienc*” OR “mental retardation” OR “kidney disease*” OR “kidney disorder*” OR “renal disease*” OR “renal disorder*” OR “mental disorder*” OR “mental* ill*” OR “psychiatric disease*” OR “psychiatric disorder*” OR “psychiatric illness∗” OR “musculoskeletal disease*” OR “musculoskeletal disorder*” OR “MSK*” OR “MSD*” OR “orthopedic disorder*” OR “orthopedic disease*” OR “neoplasm*” OR “cancer*” OR “tumor*” OR “tumour*” OR “oncolog*” OR “carcinoma*” OR “malignan*” OR “leukemia*” OR “leukaemia*” OR “lymphoma*” OR “dementia praecox” OR “schizophreni*” OR “spina bifida*” OR “cleft spine*” OR “open spine*” OR “rachischis*” OR “schistorrhach*” OR “spinal dysraphi*” OR “status dysraphicus” OR “thalassemia*” OR “chromosomal triplication*” OR “trisom*” |
| #6 | #4 OR #5 |
| #7 | “Parents” [Mesh] OR “Caregivers” [Mesh] OR “Mothers” [Mesh] OR “Fathers” [Mesh] OR “Family” [Mesh] |
| #8 | “parent*” OR “caregiver*” OR “carer*” OR “mother*” OR “father*” OR “famil*” OR “maternal*” OR “paternal*” |
| #9 | #7 OR #8 |
| #10 | “Problem Solving” [Mesh] |
| #11 | “problem-solving” OR “problem solving therap*” OR “problem-solving therap*” OR “problem solving intervention*” OR “problem-solving intervention*” OR “problem solving train*” OR “problem-solving train*” OR “problem solving program*” OR “problem-solving program*” OR “problem solving treatment” OR “problem-solving treatment” OR “problem solving workshop” OR “problem-solving workshop” OR “problem solving education” OR “problem-solving education” OR “problem solving approach” OR “problem-solving approach” OR “problem solving skills train*” OR “problem-solving skills train*” OR “PST” OR “PSST” |
| #12 | #10 OR #11 |
| #13 | “Clinical Trial” [Publication Type] OR “Controlled Clinical Trial” [Publication Type] OR “Randomized Controlled Trial” [Publication Type] OR “Controlled Clinical Trials as Topic” [Mesh] OR “Random Allocation” [Mesh] OR “Double-Blind Method” [Mesh] OR “Single-Blind Method” [Mesh] |
| #14 | “randomized controlled trial*” OR “RCT” OR “random*” OR “trial” OR “clinical trial*” OR “single blind*” OR “double blind*” OR “triple blind*” OR “waitlist*” OR “wait list*” OR “waiting list*” |
| #15 | #13 OR #14 |
| #16 | #3 AND #6 AND #9 AND #12 AND #15 |

© 2024 American Medical Association. All rights reserved.

**Embase**
Search date: 30/04/2023
Number of results: 720

| No. | Search strategies |
|---|---|
| #1 | “Child”/exp OR “Pediatrics”/exp OR “Adolescent”/exp OR “Infant”/exp OR “Minors”/exp |
| #2 | (“child*” OR “kid*” OR “boy*” OR “girl*” OR “preschool*” OR “schoolchild” OR “pediatric*” OR “paediatric*” OR “adolescen*” OR “teen*” OR “preteen*” OR “youth*” OR “juvenil*” OR “young” OR “puber” OR “pubescen*” OR “underage*” OR “under-age*” OR “infan*” OR “neonat*” OR “neo-nat*” OR “newborn*” OR “new-born*” OR “baby” OR “babies” OR “toddler*” OR “minor*” OR “offspring*” OR “young people”):ti,ab,kw |
| #3 | #1 OR #2 |
| #4 | “Chronic Disease”/exp OR “Anemia”/exp OR “Arthritis”/exp OR “Asthma”/exp OR “Attention Deficit Disorder with Hyperactivity”/exp OR “Autism Spectrum Disorder”/exp OR “Asperger Syndrome”/exp OR “Autistic Disorder”/exp OR “Vision Disorders”/exp OR “Brain Injuries”/exp OR “Bronchitis”/exp OR “Cerebral Palsy”/exp OR “Congenital Abnormalities”/exp OR “Cystic Fibrosis”/exp OR “Developmental Disabilities”/exp OR “Diabetes Mellitus”/exp OR “Down Syndrome”/exp OR “Epilepsy”/exp OR “Genetic Diseases, Inborn”/exp OR “Heart Defects, Congenital”/exp OR “Heart Diseases”/exp OR “Hematologic Diseases”/exp OR “Anemia, Sickle Cell”/exp OR “Hemophilia A”/exp OR “HIV”/exp OR “Intellectual Disability”/exp OR “Kidney Diseases”/exp OR “Mental Disorders”/exp OR “Musculoskeletal Diseases”/exp OR “Neoplasms”/exp OR “Schizophrenia”/exp OR “Spinal Dysraphism”/exp OR “Thalassemia”/exp OR “Trisomy”/exp |
| #5 | (“chronic disease*” OR “chronic ill*” OR “chronically ill*” OR “chronic disorder*” OR “chronic health” OR “chronic condition*” OR “anemia*” OR “arthriti*” OR “osteoarthriti*” OR “polyarthriti*” OR “asthma*” OR “ADHD” OR “attention deficit disorder*” OR “autism” OR “autistic” OR “asperger disease*” OR “asperger disorders” OR “asperger syndrome” OR “kanner* syndrome” OR “ASD” OR “blindness” OR “amaurosis” OR “vision loss*” OR “loss of vision” OR “vision disorder*” OR “visual* impair*” OR “brain injur*” OR “brain laceration*” OR “TBI” OR “TBIS” OR “bronchiti*” OR “cerebral pals*” OR “little* disease” OR “spastic diplegia*” OR “birth defect*” OR “congenital abnormalit*” OR “congenital anomalies” OR “congenital defect*” OR “deformit*” OR “cystic fibrosis” OR “fibrocystic disease of pancreas” OR “mucoviscidosis” OR “pancreas fibrocystic disease*” OR “development* deviation*” OR “development* disab*” OR “development* disorder*” OR “developmental delay disorder*” OR “diabetes” OR “down* syndrome” OR “mongolism” OR “trisomy 21” OR “trisomy g” OR “epilep*” OR “seizure disorder*” OR “genetic disease*” OR “genetic disorder*” OR “hereditary disease*” OR “single gene defects” OR “cardiac disease*” OR “cardiac disorder*” OR “heart disease*” OR “heart disorder*” OR “heart defect*” OR “heart abnormalit*” OR “heart malform*” OR “blood disease*” OR “blood disorder*” OR “haematologic* disease*” OR “hematologic* disease*” OR “haematologic* disorder*” OR “hematologic* disorder*” |

© 2024 American Medical Association. All rights reserved.

| | |
|---|---|
| | OR “sickle cell anemia*” OR “sickle cell disease*” OR “sickle cell disorder*” OR “hbs disease” OR “hemoglobin s disease” OR “haemophilia*” OR “hemophilia*” OR “factor viii deficien*” OR “factor 8 deficien*” OR “aids virus*” OR “acquired immune deficiency syndrome virus*” OR “AIDS virus*” OR “hiv” OR “htlv-iii” OR “HIV*” OR “human t cell leukemia virus type iii” OR “human t cell lymphotropic virus type iii” OR “human t lymphotropic virus type iii” OR “lav-htlv-iii” OR “lymphadenopathy associated virus*” OR “intellectual development disorder*” OR “intellectual disabilit*” OR “mental deficienc*” OR “mental retardation” OR “kidney disease*” OR “kidney disorder*” OR “renal disease*” OR “renal disorder*” OR “mental disorder*” OR “mental* ill*” OR “psychiatric disease*” OR “psychiatric disorder*” OR “psychiatric illness∗” OR “musculoskeletal disease*” OR “musculoskeletal disorder*” OR “MSK*” OR “MSD*” OR “orthopedic disorder*” OR “orthopedic disease*” OR “neoplasm*” OR “cancer*” OR “tumor*” OR “tumour*” OR “oncolog*” OR “carcinoma*” OR “malignan*” OR “leukemia*” OR “leukaemia*” OR “lymphoma*” OR “dementia praecox” OR “schizophreni*” OR “spina bifida*” OR “cleft spine*” OR “open spine*” OR “rachischis*” OR “schistorrhach*” OR “spinal dysraphi*” OR “status dysraphicus” OR “thalassemia*” OR “chromosomal triplication*” OR “trisom*”):ti,ab,kw |
| #6 | #4 OR #5 |
| #7 | “Parents”/exp OR “Caregivers”/exp OR “Mothers”/exp OR “Fathers”/exp OR “Family”/exp |
| #8 | (“parent*” OR “caregiver*” OR “carer*” OR “mother*” OR “father*” OR “famil*” OR “maternal*” OR “paternal*”):ti,ab,kw |
| #9 | #7 OR #8 |
| #10 | “Problem Solving”/exp |
| #11 | (“problem-solving” OR “problem solving therap*” OR “problem-solving therap*” OR “problem solving intervention*” OR “problem-solving intervention*” OR “problem solving train*” OR “problem-solving train*” OR “problem solving program*” OR “problem-solving program*” OR “problem solving treatment” OR “problem-solving treatment” OR “problem solving workshop” OR “problem-solving workshop” OR “problem solving education” OR “problem-solving education” OR “problem solving approach” OR “problem-solving approach” OR “problem solving skills train*” OR “problem-solving skills train*” OR “PST” OR “PSST”):ti,ab,kw |
| #12 | #10 OR #11 |
| #13 | “Clinical Trial”/de OR “Controlled Clinical Trial”/de OR “Randomized Controlled Trial”/de OR “Controlled Clinical Trial (topic)”/exp OR “Random Allocation”/exp OR “Double-Blind Method”/exp OR “Single-Blind Method”/exp |
| #14 | (“randomized controlled trial*” OR “RCT” OR “random*” OR “trial” OR “clinical trial*” OR “single blind*” OR “double blind*” OR “triple blind*” OR “waitlist*” OR “wait list*” OR “waiting list*”):ti,ab,kw |
| #15 | #13 OR #14 |
| #16 | #3 AND #6 AND #9 AND #12 AND #15 |

© 2024 American Medical Association. All rights reserved.

**CINAHL**
Search date: 30/04/2023
Number of results: 131

| No. | Search strategies |
|---|---|
| #1 | (MH "Child") OR (MH "Pediatrics") OR (MH "Adolescent") OR (MH "Infant") OR (MH "Minors") |
| #2 | TI ("child*" OR "kid*" OR "boy*" OR "girl*" OR "preschool*" OR "schoolchild" OR "pediatric*" OR "paediatric*" OR "adolescen*" OR "teen*" OR "preteen*" OR "youth*" OR "juvenil*" OR "young" OR "puber" OR "pubescen*" OR "underage*" OR "under-age*" OR "infan*" OR "neonat*" OR "neo-nat*" OR "newborn*" OR "new-born*" OR "baby" OR "babies" OR "toddler*" OR "minor*" OR "offspring*" OR "young people") OR "AB (child*" OR "kid*" OR "boy*" OR "girl*" OR "preschool*" OR "schoolchild" OR "pediatric*" OR "paediatric*" OR "adolescen*" OR "teen*" OR "preteen*" OR "youth*" OR "juvenil*" OR "young" OR "puber" OR "pubescen*" OR "underage*" OR "under-age*" OR "infan*" OR "neonat*" OR "neo-nat*" OR "newborn*" OR "new-born*" OR "baby" OR "babies" OR "toddler*" OR "minor*" OR "offspring*" OR "young people") |
| #3 | #1 OR #2 |
| #4 | (MH "Chronic Disease") OR (MH "Anemia") OR (MH "Arthritis") OR (MH "Asthma") OR (MH "Attention Deficit Disorder with Hyperactivity") OR (MH "Autism Spectrum Disorder") OR (MH "Asperger Syndrome") OR (MH "Autistic Disorder") OR (MH "Vision Disorders") OR (MH "Brain Injuries") OR (MH "Bronchitis") OR (MH "Cerebral Palsy") OR (MH "Congenital Abnormalities") OR (MH "Cystic Fibrosis") OR (MH "Developmental Disabilities") OR (MH "Diabetes Mellitus") OR (MH "Down Syndrome") OR (MH "Epilepsy") OR (MH "Genetic Diseases, Inborn") OR (MH "Heart Defects, Congenital") OR (MH "Heart Diseases") OR (MH "Hematologic Diseases") OR (MH "Anemia, Sickle Cell") OR (MH "Hemophilia A") OR (MH "HIV") OR (MH "Intellectual Disability") OR (MH "Kidney Diseases") OR (MH "Mental Disorders") OR (MH "Musculoskeletal Diseases") OR (MH "Neoplasms") OR (MH "Schizophrenia") OR (MH "Spinal Dysraphism") OR (MH "Thalassemia") OR (MH "Trisomy") |
| #5 | TI ("chronic disease*" OR "chronic ill*" OR "chronically ill*" OR "chronic disorder*" OR "chronic health" OR "chronic condition*" OR "anemia*" OR "arthriti*" OR "osteoarthriti*" OR "polyarthriti*" OR "asthma*" OR "ADHD" OR "attention deficit disorder*" OR "autism" OR "autistic" OR "asperger disease*" OR "asperger disorders" OR "asperger syndrome" OR "kanner* syndrome" OR "ASD" OR "blindness" OR "amaurosis" OR "vision loss*" OR "loss of vision" OR "vision disorder*" OR "visual* impair*" OR "brain injur*" OR "brain laceration*" OR "TBI" OR "TBIS" OR "bronchiti*" OR "cerebral pals*" OR "little* disease" OR "spastic diplegia*" OR "birth defect*" OR "congenital abnormalit*" OR "congenital anomalies" OR "congenital defect*" OR "deformit*" OR "cystic fibrosis" OR "fibrocystic disease of pancreas" OR "mucoviscidosis" OR "pancreas fibrocystic disease*" OR "development* deviation*" OR "development* disab*" OR "development* disorder*" OR "developmental delay disorder*" OR "diabetes" OR "down* syndrome" OR |

© 2024 American Medical Association. All rights reserved.

| | |
|---|---|
| | "mongolism" OR "trisomy 21" OR "trisomy g" OR "epilep*" OR "seizure disorder*" OR "genetic disease*" OR "genetic disorder*" OR "hereditary disease*" OR "single gene defects" OR "cardiac disease*" OR "cardiac disorder*" OR "heart disease*" OR "heart disorder*" OR "heart defect*" OR "heart abnormalit*" OR "heart malform*" OR "blood disease*" OR "blood disorder*" OR "haematologic* disease*" OR "hematologic* disease*" OR "haematologic* disorder*" OR "hematologic* disorder*" OR "sickle cell anemia*" OR "sickle cell disease*" OR "sickle cell disorder*" OR "hbs disease" OR "hemoglobin s disease" OR "haemophilia*" OR "hemophilia*" OR "factor viii deficien*" OR "factor 8 deficien*" OR "aids virus*" OR "acquired immune deficiency syndrome virus*" OR "AIDS virus*" OR "hiv" OR "htlv-iii" OR "HIV*" OR "human t cell leukemia virus type iii" OR "human t cell lymphotropic virus type iii" OR "human t lymphotropic virus type iii" OR "lav-htlv-iii" OR "lymphadenopathy associated virus*" OR "intellectual development disorder*" OR "intellectual disabilit*" OR "mental deficienc*" OR "mental retardation" OR "kidney disease*" OR "kidney disorder*" OR "renal disease*" OR "renal disorder*" OR "mental disorder*" OR "mental* ill*" OR "psychiatric disease*" OR "psychiatric disorder*" OR "psychiatric illness∗" OR "musculoskeletal disease*" OR "musculoskeletal disorder*" OR "MSK*" OR "MSD*" OR "orthopedic disorder*" OR "orthopedic disease*" OR "neoplasm*" OR "cancer*" OR "tumor*" OR "tumour*" OR "oncolog*" OR "carcinoma*" OR "malignan*" OR "leukemia*" OR "leukaemia*" OR "lymphoma*" OR "dementia praecox" OR "schizophreni*" OR "spina bifida*" OR "cleft spine*" OR "open spine*" OR "rachischis*" OR "schistorrhach*" OR "spinal dysraphi*" OR "status dysraphicus" OR "thalassemia*" OR "chromosomal triplication*" OR "trisom*") OR AB ("chronic disease*" OR "chronic ill*" OR "chronically ill*" OR "chronic disorder*" OR "chronic health" OR "chronic condition*" OR "anemia*" OR "arthriti*" OR "osteoarthriti*" OR "polyarthriti*" OR "asthma*" OR "ADHD" OR "attention deficit disorder*" OR "autism" OR "autistic" OR "asperger disease*" OR "asperger disorders" OR "asperger syndrome" OR "kanner* syndrome" OR "ASD" OR "blindness" OR "amaurosis" OR "vision loss*" OR "loss of vision" OR "vision disorder*" OR "visual* impair*" OR "brain injur*" OR "brain laceration*" OR "TBI" OR "TBIS" OR "bronchiti*" OR "cerebral pals*" OR "little* disease" OR "spastic diplegia*" OR "birth defect*" OR "congenital abnormalit*" OR "congenital anomalies" OR "congenital defect*" OR "deformit*" OR "cystic fibrosis" OR "fibrocystic disease of pancreas" OR "mucoviscidosis" OR "pancreas fibrocystic disease*" OR "development* deviation*" OR "development* disab*" OR "development* disorder*" OR "developmental delay disorder*" OR "diabetes" OR "down* syndrome" OR "mongolism" OR "trisomy 21" OR "trisomy g" OR "epilep*" OR "seizure disorder*" OR "genetic disease*" OR "genetic disorder*" OR "hereditary disease*" OR "single gene defects" OR "cardiac disease*" OR "cardiac disorder*" OR "heart disease*" OR "heart disorder*" OR "heart defect*" OR "heart abnormalit*" OR "heart malform*" OR "blood disease*" OR "blood disorder*" OR "haematologic* disease*" OR "hematologic* disease*" OR "haematologic* disorder*" OR "hematologic* disorder*" OR "sickle cell anemia*" OR "sickle cell disease*" OR "sickle cell disorder*" OR "hbs disease" OR "hemoglobin s disease" OR "haemophilia*" OR "hemophilia*" OR "factor viii deficien*" OR "factor 8 deficien*" OR "aids virus*" OR "acquired immune deficiency syndrome virus*" OR |

© 2024 American Medical Association. All rights reserved.

| | |
|---|---|
| | “AIDS virus*” OR “hiv” OR “htlv-iii” OR “HIV*” OR “human t cell leukemia virus type iii” OR “human t cell lymphotropic virus type iii” OR “human t lymphotropic virus type iii” OR “lav-htlv-iii” OR “lymphadenopathy associated virus*” OR “intellectual development disorder*” OR “intellectual disabilit*” OR “mental deficienc*” OR “mental retardation” OR “kidney disease*” OR “kidney disorder*” OR “renal disease*” OR “renal disorder*” OR “mental disorder*” OR “mental* ill*” OR “psychiatric disease*” OR “psychiatric disorder*” OR “psychiatric illness∗” OR “musculoskeletal disease*” OR “musculoskeletal disorder*” OR “MSK*” OR “MSD*” OR “orthopedic disorder*” OR “orthopedic disease*” OR “neoplasm*” OR “cancer*” OR “tumor*” OR “tumour*” OR “oncolog*” OR “carcinoma*” OR “malignan*” OR “leukemia*” OR “leukaemia*” OR “lymphoma*” OR “dementia praecox” OR “schizophreni*” OR “spina bifida*” OR “cleft spine*” OR “open spine*” OR “rachischis*” OR “schistorrhach*” OR “spinal dysraphi*” OR “status dysraphicus” OR “thalassemia*” OR “chromosomal triplication*” OR “trisom*”) |
| #6 | #4 OR #5 |
| #7 | (MH “Parents”) OR (MH “Caregivers”) OR (MH “Mothers”) OR (MH “Fathers”) OR (MH “Family”) |
| #8 | TI (“parent*” OR “caregiver*” OR “carer*” OR “mother*” OR “father*” OR “famil*” OR “maternal*” OR “paternal*”) OR AB (“parent*” OR “caregiver*” OR “carer*” OR “mother*” OR “father*” OR “famil*” OR “maternal*” OR “paternal*”) |
| #9 | #7 OR #8 |
| #10 | (MH “Problem Solving”) |
| #11 | TI (“problem-solving” OR “problem solving therap*” OR “problem-solving therap*” OR “problem solving intervention*” OR “problem-solving intervention*” OR “problem solving train*” OR “problem-solving train*” OR “problem solving program*” OR “problem-solving program*” OR “problem solving treatment” OR “problem-solving treatment” OR “problem solving workshop” OR “problem-solving workshop” OR “problem solving education” OR “problem-solving education” OR “problem solving approach” OR “problem-solving approach” OR “problem solving skills train*” OR “problem-solving skills train*” OR “PST” OR “PSST”) OR AB (“problem-solving” OR “problem solving therap*” OR “problem-solving therap*” OR “problem solving intervention*” OR “problem-solving intervention*” OR “problem solving train*” OR “problem-solving train*” OR “problem solving program*” OR “problem-solving program*” OR “problem solving treatment” OR “problem-solving treatment” OR “problem solving workshop” OR “problem-solving workshop” OR “problem solving education” OR “problem-solving education” OR “problem solving approach” OR “problem-solving approach” OR “problem solving skills train*” OR “problem-solving skills train*” OR “PST” OR “PSST”) |
| #12 | #10 OR #11 |
| #13 | (MH “Clinical Trial”) OR (MH “Controlled Clinical Trial”) OR (MH “Randomized Controlled Trial”) OR (MH “Controlled Clinical Trials as Topic”) OR (MH “Random Allocation”) OR (MH “Double-Blind Method”) OR (MH “Single-Blind Method”) |
| #14 | TI (“randomized controlled trial*” OR “RCT” OR “random*” OR “trial” OR “clinical trial*” OR “single blind*” OR “double blind*” OR “triple blind*” OR “waitlist*” OR “wait list*” OR “waiting list*”) OR AB (“randomized controlled trial*” OR “RCT” OR |

© 2024 American Medical Association. All rights reserved.

| | |
|---|---|
| | "random*" OR "trial" OR "clinical trial*" OR "single blind*" OR "double blind*" OR "triple blind*" OR "waitlist*" OR "wait list*" OR "waiting list*") |
| #15 | #13 OR #14 |
| #16 | #3 AND #6 AND #9 AND #12 AND #15 |

**PsycINFO**

Search date: 30/04/2023

Number of results: 170

| No. | Search strategies |
|---|---|
| #1 | (MA "Child") OR (MA "Pediatrics") OR (MA "Adolescent") OR (MA "Infant") OR (MA "Minors") |
| #2 | TI ("child*" OR "kid*" OR "boy*" OR "girl*" OR "preschool*" OR "schoolchild" OR "pediatric*" OR "paediatric*" OR "adolescen*" OR "teen*" OR "preteen*" OR "youth*" OR "juvenil*" OR "young" OR "puber" OR "pubescen*" OR "underage*" OR "under-age*" OR "infan*" OR "neonat*" OR "neo-nat*" OR "newborn*" OR "new-born*" OR "baby" OR "babies" OR "toddler*" OR "minor*" OR "offspring*" OR "young people") OR "AB (child*" OR "kid*" OR "boy*" OR "girl*" OR "preschool*" OR "schoolchild" OR "pediatric*" OR "paediatric*" OR "adolescen*" OR "teen*" OR "preteen*" OR "youth*" OR "juvenil*" OR "young" OR "puber" OR "pubescen*" OR "underage*" OR "under-age*" OR "infan*" OR "neonat*" OR "neo-nat*" OR "newborn*" OR "new-born*" OR "baby" OR "babies" OR "toddler*" OR "minor*" OR "offspring*" OR "young people") |
| #3 | #1 OR #2 |
| #4 | (MA "Chronic Disease") OR (MA "Anemia") OR (MA "Arthritis") OR (MA "Asthma") OR (MA "Attention Deficit Disorder with Hyperactivity") OR (MA "Autism Spectrum Disorder") OR (MA "Asperger Syndrome") OR (MA "Autistic Disorder") OR (MA "Vision Disorders") OR (MA "Brain Injuries") OR (MA "Bronchitis") OR (MA "Cerebral Palsy") OR (MA "Congenital Abnormalities") OR (MA "Cystic Fibrosis") OR (MA "Developmental Disabilities") OR (MA "Diabetes Mellitus") OR (MA "Down Syndrome") OR (MA "Epilepsy") OR (MA "Genetic Diseases, Inborn") OR (MA "Heart Defects, Congenital") OR (MA "Heart Diseases") OR (MA "Hematologic Diseases") OR (MA "Anemia, Sickle Cell") OR (MA "Hemophilia A") OR (MA "HIV") OR (MA "Intellectual Disability") OR (MA "Kidney Diseases") OR (MA "Mental Disorders") OR (MA "Musculoskeletal Diseases") OR (MA "Neoplasms") OR (MA "Schizophrenia") OR (MA "Spinal Dysraphism") OR (MA "Thalassemia") OR (MA "Trisomy") |
| #5 | TI ("chronic disease*" OR "chronic ill*" OR "chronically ill*" OR "chronic disorder*" OR "chronic health" OR "chronic condition*" OR "anemia*" OR "arthriti*" OR "osteoarthriti*" OR "polyarthriti*" OR "asthma*" OR "ADHD" OR "attention deficit disorder*" OR "autism" OR "autistic" OR "asperger disease*" OR "asperger disorders" OR "asperger syndrome" OR "kanner* syndrome" OR "ASD" OR "blindness" OR "amaurosis" OR "vision loss*" OR "loss of vision" OR "vision disorder*" OR "visual* impair*" OR "brain injur*" OR "brain laceration*" OR "TBI" OR |

© 2024 American Medical Association. All rights reserved.

| | |
|---|---|
| | “TBIS” OR “bronchiti*” OR “cerebral pals*” OR “little* disease” OR “spastic diplegia*” OR “birth defect*” OR “congenital abnormalit*” OR “congenital anomalies” OR “congenital defect*” OR “deformit*” OR “cystic fibrosis” OR “fibrocystic disease of pancreas” OR “mucoviscidosis” OR “pancreas fibrocystic disease*” OR “development* deviation*” OR “development* disab*” OR “development* disorder*” OR “developmental delay disorder*” OR “diabetes” OR “down* syndrome” OR “mongolism” OR “trisomy 21” OR “trisomy g” OR “epilep*” OR “seizure disorder*” OR “genetic disease*” OR “genetic disorder*” OR “hereditary disease*” OR “single gene defects” OR “cardiac disease*” OR “cardiac disorder*” OR “heart disease*” OR “heart disorder*” OR “heart defect*” OR “heart abnormalit*” OR “heart malform*” OR “blood disease*” OR “blood disorder*” OR “haematologic* disease*” OR “hematologic* disease*” OR “haematologic* disorder*” OR “hematologic* disorder*” OR “sickle cell anemia*” OR “sickle cell disease*” OR “sickle cell disorder*” OR “hbs disease” OR “hemoglobin s disease” OR “haemophilia*” OR “hemophilia*” OR “factor viii deficien*” OR “factor 8 deficien*” OR “aids virus*” OR “acquired immune deficiency syndrome virus*” OR “AIDS virus*” OR “hiv” OR “htlv-iii” OR “HIV*” OR “human t cell leukemia virus type iii” OR “human t cell lymphotropic virus type iii” OR “human t lymphotropic virus type iii” OR “lav-htlv-iii” OR “lymphadenopathy associated virus*” OR “intellectual development disorder*” OR “intellectual disabilit*” OR “mental deficienc*” OR “mental retardation” OR “kidney disease*” OR “kidney disorder*” OR “renal disease*” OR “renal disorder*” OR “mental disorder*” OR “mental* ill*” OR “psychiatric disease*” OR “psychiatric disorder*” OR “psychiatric illness*” OR “musculoskeletal disease*” OR “musculoskeletal disorder*” OR “MSK*” OR “MSD*” OR “orthopedic disorder*” OR “orthopedic disease*” OR “neoplasm*” OR “cancer*” OR “tumor*” OR “tumour*” OR “oncolog*” OR “carcinoma*” OR “malignan*” OR “leukemia*” OR “leukaemia*” OR “lymphoma*” OR “dementia praecox” OR “schizophreni*” OR “spina bifida*” OR “cleft spine*” OR “open spine*” OR “rachischis*” OR “schistorrhach*” OR “spinal dysraphi*” OR “status dysraphicus” OR “thalassemia*” OR “chromosomal triplication*” OR “trisom*”) OR AB (“chronic disease*” OR “chronic ill*” OR “chronically ill*” OR “chronic disorder*” OR “chronic health” OR “chronic condition*” OR “anemia*” OR “arthriti*” OR “osteoarthriti*” OR “polyarthriti*” OR “asthma*” OR “ADHD” OR “attention deficit disorder*” OR “autism” OR “autistic” OR “asperger disease*” OR “asperger disorders” OR “asperger syndrome” OR “kanner* syndrome” OR “ASD” OR “blindness” OR “amaurosis” OR “vision loss*” OR “loss of vision” OR “vision disorder*” OR “visual* impair*” OR “brain injur*” OR “brain laceration*” OR “TBI” OR “TBIS” OR “bronchiti*” OR “cerebral pals*” OR “little* disease” OR “spastic diplegia*” OR “birth defect*” OR “congenital abnormalit*” OR “congenital anomalies” OR “congenital defect*” OR “deformit*” OR “cystic fibrosis” OR “fibrocystic disease of pancreas” OR “mucoviscidosis” OR “pancreas fibrocystic disease*” OR “development* deviation*” OR “development* disab*” OR “development* disorder*” OR “developmental delay disorder*” OR “diabetes” OR “down* syndrome” OR “mongolism” OR “trisomy 21” OR “trisomy g” OR “epilep*” OR “seizure disorder*” OR “genetic disease*” OR “genetic disorder*” OR “hereditary disease*” OR “single gene defects” OR “cardiac disease*” OR “cardiac disorder*” OR “heart disease*” OR “heart disorder*” OR |

© 2024 American Medical Association. All rights reserved.

| | |
|---|---|
| | "heart defect*" OR "heart abnormalit*" OR "heart malform*" OR "blood disease*" OR "blood disorder*" OR "haematologic* disease*" OR "hematologic* disease*" OR "haematologic* disorder*" OR "hematologic* disorder*" OR "sickle cell anemia*" OR "sickle cell disease*" OR "sickle cell disorder*" OR "hbs disease" OR "hemoglobin s disease" OR "haemophilia*" OR "hemophilia*" OR "factor viii deficien*" OR "factor 8 deficien*" OR "aids virus*" OR "acquired immune deficiency syndrome virus*" OR "AIDS virus*" OR "hiv" OR "htlv-iii" OR "HIV*" OR "human t cell leukemia virus type iii" OR "human t cell lymphotropic virus type iii" OR "human t lymphotropic virus type iii" OR "lav-htlv-iii" OR "lymphadenopathy associated virus*" OR "intellectual development disorder*" OR "intellectual disabilit*" OR "mental deficienc*" OR "mental retardation" OR "kidney disease*" OR "kidney disorder*" OR "renal disease*" OR "renal disorder*" OR "mental disorder*" OR "mental* ill*" OR "psychiatric disease*" OR "psychiatric disorder*" OR "psychiatric illness∗" OR "musculoskeletal disease*" OR "musculoskeletal disorder*" OR "MSK*" OR "MSD*" OR "orthopedic disorder*" OR "orthopedic disease*" OR "neoplasm*" OR "cancer*" OR "tumor*" OR "tumour*" OR "oncolog*" OR "carcinoma*" OR "malignan*" OR "leukemia*" OR "leukaemia*" OR "lymphoma*" OR "dementia praecox" OR "schizophreni*" OR "spina bifida*" OR "cleft spine*" OR "open spine*" OR "rachischis*" OR "schistorrhach*" OR "spinal dysraphi*" OR "status dysraphicus" OR "thalassemia*" OR "chromosomal triplication*" OR "trisom*") |
| #6 | #4 OR #5 |
| #7 | (MA "Parents") OR (MA "Caregivers") OR (MA "Mothers") OR (MA "Fathers") OR (MA "Family") |
| #8 | TI ("parent*" OR "caregiver*" OR "carer*" OR "mother*" OR "father*" OR "famil*" OR "maternal*" OR "paternal*") OR AB ("parent*" OR "caregiver*" OR "carer*" OR "mother*" OR "father*" OR "famil*" OR "maternal*" OR "paternal*") |
| #9 | #7 OR #8 |
| #10 | (MA "Problem Solving") |
| #11 | TI ("problem-solving" OR "problem solving therap*" OR "problem-solving therap*" OR "problem solving intervention*" OR "problem-solving intervention*" OR "problem solving train*" OR "problem-solving train*" OR "problem solving program*" OR "problem-solving program*" OR "problem solving treatment" OR "problem-solving treatment" OR "problem solving workshop" OR "problem-solving workshop" OR "problem solving education" OR "problem-solving education" OR "problem solving approach" OR "problem-solving approach" OR "problem solving skills train*" OR "problem-solving skills train*" OR "PST" OR "PSST") OR AB ("problem-solving" OR "problem solving therap*" OR "problem-solving therap*" OR "problem solving intervention*" OR "problem-solving intervention*" OR "problem solving train*" OR "problem-solving train*" OR "problem solving program*" OR "problem-solving program*" OR "problem solving treatment" OR "problem-solving treatment" OR "problem solving workshop" OR "problem-solving workshop" OR "problem solving education" OR "problem-solving education" OR "problem solving approach" OR "problem-solving approach" OR "problem solving skills train*" OR "problem-solving skills train*" OR "PST" OR "PSST") |
| #12 | #10 OR #11 |

© 2024 American Medical Association. All rights reserved.

| | |
|---|---|
| #13 | (MA "Clinical Trial") OR (MA "Controlled Clinical Trial") OR (MA "Randomized Controlled Trial") OR (MA "Controlled Clinical Trials as Topic") OR (MA "Random Allocation") OR (MA "Double-Blind Method") OR (MA "Single-Blind Method") |
| #14 | TI ("randomized controlled trial*" OR "RCT" OR "random*" OR "trial" OR "clinical trial*" OR "single blind*" OR "double blind*" OR "triple blind*" OR "waitlist*" OR "wait list*" OR "waiting list*") OR AB ("randomized controlled trial*" OR "RCT" OR "random*" OR "trial" OR "clinical trial*" OR "single blind*" OR "double blind*" OR "triple blind*" OR "waitlist*" OR "wait list*" OR "waiting list*") |
| #15 | #13 OR #14 |
| #16 | #3 AND #6 AND #9 AND #12 AND #15 |

**Web of Science**
Search date: 30/04/2023
Number of results: 474

| No. | Search strategies |
|---|---|
| #1 | TS=("child*" OR "kid*" OR "boy*" OR "girl*" OR "preschool*" OR "schoolchild" OR "pediatric*" OR "paediatric*" OR "adolescen*" OR "teen*" OR "preteen*" OR "youth*" OR "juvenil*" OR "young" OR "puber" OR "pubescen*" OR "underage*" OR "under-age*" OR "infan*" OR "neonat*" OR "neo-nat*" OR "newborn*" OR "new-born*" OR "baby" OR "babies" OR "toddler*" OR "minor*" OR "offspring*" OR "young people") |
| #2 | TS=("chronic disease*" OR "chronic ill*" OR "chronically ill*" OR "chronic disorder*" OR "chronic health" OR "chronic condition*" OR "anemia*" OR "arthriti*" OR "osteoarthriti*" OR "polyarthriti*" OR "asthma*" OR "ADHD" OR "attention deficit disorder*" OR "autism" OR "autistic" OR "asperger disease*" OR "asperger disorders" OR "asperger syndrome" OR "kanner* syndrome" OR "ASD" OR "blindness" OR "amaurosis" OR "vision loss*" OR "loss of vision" OR "vision disorder*" OR "visual* impair*" OR "brain injur*" OR "brain laceration*" OR "TBI" OR "TBIS" OR "bronchiti*" OR "cerebral pals*" OR "little* disease" OR "spastic diplegia*" OR "birth defect*" OR "congenital abnormalit*" OR "congenital anomalies" OR "congenital defect*" OR "deformit*" OR "cystic fibrosis" OR "fibrocystic disease of pancreas" OR "mucoviscidosis" OR "pancreas fibrocystic disease*" OR "development* deviation*" OR "development* disab*" OR "development* disorder*" OR "developmental delay disorder*" OR "diabetes" OR "down* syndrome" OR "mongolism" OR "trisomy 21" OR "trisomy g" OR "epilep*" OR "seizure disorder*" OR "genetic disease*" OR "genetic disorder*" OR "hereditary disease*" OR "single gene defects" OR "cardiac disease*" OR "cardiac disorder*" OR "heart disease*" OR "heart disorder*" OR "heart defect*" OR "heart abnormalit*" OR "heart malform*" OR "blood disease*" OR "blood disorder*" OR "haematologic* disease*" OR "hematologic* disease*" OR "haematologic* disorder*" OR "hematologic* disorder*" OR "sickle cell anemia*" OR "sickle cell disease*" OR "sickle cell disorder*" OR "hbs disease" OR "hemoglobin s disease" OR "haemophilia*" OR "hemophilia*" OR "factor viii deficien*" OR "factor 8 |

© 2024 American Medical Association. All rights reserved.

| | |
|---|---|
| | deficien*" OR "aids virus*" OR "acquired immune deficiency syndrome virus*" OR "AIDS virus*" OR "hiv" OR "htlv-iii" OR "HIV*" OR "human t cell leukemia virus type iii" OR "human t cell lymphotropic virus type iii" OR "human t lymphotropic virus type iii" OR "lav-htlv-iii" OR "lymphadenopathy associated virus*" OR "intellectual development disorder*" OR "intellectual disabilit*" OR "mental deficienc*" OR "mental retardation" OR "kidney disease*" OR "kidney disorder*" OR "renal disease*" OR "renal disorder*" OR "mental disorder*" OR "mental* ill*" OR "psychiatric disease*" OR "psychiatric disorder*" OR "psychiatric illness∗" OR "musculoskeletal disease*" OR "musculoskeletal disorder*" OR "MSK*" OR "MSD*" OR "orthopedic disorder*" OR "orthopedic disease*" OR "neoplasm*" OR "cancer*" OR "tumor*" OR "tumour*" OR "oncolog*" OR "carcinoma*" OR "malignan*" OR "leukemia*" OR "leukaemia*" OR "lymphoma*" OR "dementia praecox" OR "schizophreni*" OR "spina bifida*" OR "cleft spine*" OR "open spine*" OR "rachischis*" OR "schistorrhach*" OR "spinal dysraphi*" OR "status dysraphicus" OR "thalassemia*" OR "chromosomal triplication*" OR "trisom*") |
| #3 | TS=("parent*" OR "caregiver*" OR "carer*" OR "mother*" OR "father*" OR "famil*" OR "maternal*" OR "paternal*") |
| #4 | TS=("problem solving" OR "problem-solving" OR "problem solving therap*" OR "problem-solving therap*" OR "problem solving intervention*" OR "problem-solving intervention*" OR "problem solving train*" OR "problem-solving train*" OR "problem solving program*" OR "problem-solving program*" OR "problem solving treatment" OR "problem-solving treatment" OR "problem solving workshop" OR "problem-solving workshop" OR "problem solving education" OR "problem-solving education" OR "problem solving approach" OR "problem-solving approach" OR "problem solving skills train*" OR "problem-solving skills train*" OR "PST" OR "PSST") |
| #5 | TS=("randomized controlled trial*" OR "RCT" OR "random*" OR "trial" OR "clinical trial*" OR "single blind*" OR "double blind*" OR "triple blind*" OR "waitlist*" OR "wait list*" OR "waiting list*") |
| #6 | #1 AND #2 AND #3 AND #4 AND #5 |

**Cochrane Library**
Search date: 30/04/2023
Number of results: 450

| No. | Search strategies |
|---|---|
| #1 | (MeSH descriptor: [Child] explode all trees) OR (MeSH descriptor: [Pediatrics] explode all trees) OR (MeSH descriptor: [Adolescent] explode all trees) OR (MeSH descriptor: [Infant] explode all trees) OR (MeSH descriptor: [Minors] explode all trees) |
| #2 | ("child*" OR "kid*" OR "preschool*" OR "pediatric*" OR "paediatric*" OR "adolescen*" OR "teen*" OR "youth*" OR "juvenil*" OR "young" OR "puber" OR "pubescen*" OR "underage*" OR "under-age*" OR "infan*" OR "neonat*" OR "neo- |

© 2024 American Medical Association. All rights reserved.

| | |
|---|---|
| | nat*" OR "newborn*" OR "new-born*" OR "baby" OR "babies" OR "toddler*" OR "minor*" OR "offspring*" OR "young people").ti,ab,kw |
| #3 | #1 OR #2 |
| #4 | (MeSH descriptor: [Chronic Disease] explode all trees) OR (MeSH descriptor: [Anemia] explode all trees) OR (MeSH descriptor: [Arthritis] explode all trees) OR (MeSH descriptor: [Asthma] explode all trees) OR (MeSH descriptor: [Attention Deficit Disorder with Hyperactivity] explode all trees) OR (MeSH descriptor: [Autism Spectrum Disorder] explode all trees) OR (MeSH descriptor: [Asperger Syndrome] explode all trees) OR (MeSH descriptor: [Autistic Disorder] explode all trees) OR (MeSH descriptor: [Vision Disorders] explode all trees) OR (MeSH descriptor: [Brain Injuries] explode all trees) OR (MeSH descriptor: [Bronchitis] explode all trees) OR (MeSH descriptor: [Cerebral Palsy] explode all trees) OR (MeSH descriptor: [Congenital Abnormalities] explode all trees) OR (MeSH descriptor: [Cystic Fibrosis] explode all trees) OR (MeSH descriptor: [Developmental Disabilities] explode all trees) OR (MeSH descriptor: [Diabetes Mellitus] explode all trees) OR (MeSH descriptor: [Down Syndrome] explode all trees) OR (MeSH descriptor: [Epilepsy] explode all trees) OR (MeSH descriptor: [Genetic Diseases, Inborn] explode all trees) OR (MeSH descriptor: [Heart Defects, Congenital] explode all trees) OR (MeSH descriptor: [Heart Diseases] explode all trees) OR (MeSH descriptor: [Hematologic Diseases] explode all trees) OR (MeSH descriptor: [Anemia, Sickle Cell] explode all trees) OR (MeSH descriptor: [Hemophilia A] explode all trees) OR (MeSH descriptor: [HIV] explode all trees) OR (MeSH descriptor: [Intellectual Disability] explode all trees) OR (MeSH descriptor: [Kidney Diseases] explode all trees) OR (MeSH descriptor: [Mental Disorders] explode all trees) OR (MeSH descriptor: [Musculoskeletal Diseases] explode all trees) OR (MeSH descriptor: [Neoplasms] explode all trees) OR (MeSH descriptor: [Schizophrenia] explode all trees) OR (MeSH descriptor: [Spinal Dysraphism] explode all trees) OR (MeSH descriptor: [Thalassemia] explode all trees) OR (MeSH descriptor: [Trisomy] explode all trees) |
| #5 | ("chronic disease*" OR "chronic ill*" OR "chronically ill*" OR "chronic disorder*" OR "chronic health" OR "chronic condition*" OR "anemia*" OR "arthriti*" OR "osteoarthriti*" OR "polyarthriti*" OR "asthma*" OR "ADHD" OR "attention deficit disorder*" OR "autism" OR "autistic" OR "asperger disease*" OR "asperger disorders" OR "asperger syndrome" OR "kanner* syndrome" OR "ASD" OR "blindness" OR "amaurosis" OR "vision loss*" OR "loss of vision" OR "vision disorder*" OR "visual* impair*" OR "brain injur*" OR "brain laceration*" OR "TBI" OR "TBIS" OR "bronchiti*" OR "cerebral pals*" OR "little* disease" OR "spastic diplegia*" OR "birth defect*" OR "congenital abnormalit*" OR "congenital anomalies" OR "congenital defect*" OR "deformit*" OR "cystic fibrosis" OR "fibrocystic disease of pancreas" OR "mucoviscidosis" OR "pancreas fibrocystic disease*" OR "development* deviation*" OR "development* disab*" OR "development* disorder*" OR "developmental delay disorder*" OR "diabetes" OR "down* syndrome" OR "mongolism" OR "trisomy 21" OR "trisomy g" OR "epilep*" OR "seizure disorder*" OR "genetic disease*" OR "genetic disorder*" OR "hereditary disease*" OR "single gene defects" OR "cardiac disease*" OR "cardiac |

© 2024 American Medical Association. All rights reserved.

| | |
|---|---|
| | disorder*” OR “heart disease*” OR “heart disorder*” OR “heart defect*” OR “heart abnormalit*” OR “heart malform*” OR “blood disease*” OR “blood disorder*” OR “haematologic* disease*” OR “hematologic* disease*” OR “haematologic* disorder*” OR “hematologic* disorder*” OR “sickle cell anemia*” OR “sickle cell disease*” OR “sickle cell disorder*” OR “hbs disease” OR “hemoglobin s disease” OR “haemophilia*” OR “hemophilia*” OR “factor viii deficien*” OR “factor 8 deficien*” OR “aids virus*” OR “acquired immune deficiency syndrome virus*” OR “AIDS virus*” OR “hiv” OR “htlv-iii” OR “HIV*” OR “human t cell leukemia virus type iii” OR “human t cell lymphotropic virus type iii” OR “human t lymphotropic virus type iii” OR “lav-htlv-iii” OR “lymphadenopathy associated virus*” OR “intellectual development disorder*” OR “intellectual disabilit*” OR “mental deficienc*” OR “mental retardation” OR “kidney disease*” OR “kidney disorder*” OR “renal disease*” OR “renal disorder*” OR “mental disorder*” OR “mental* ill*” OR “psychiatric disease*” OR “psychiatric disorder*” OR “psychiatric illness∗” OR “musculoskeletal disease*” OR “musculoskeletal disorder*” OR “MSK*” OR “MSD*” OR “orthopedic disorder*” OR “orthopedic disease*” OR “neoplasm*” OR “cancer*” OR “tumor*” OR “tumour*” OR “oncolog*” OR “carcinoma*” OR “malignan*” OR “leukemia*” OR “leukaemia*” OR “lymphoma*” OR “dementia praecox” OR “schizophreni*” OR “spina bifida*” OR “cleft spine*” OR “open spine*” OR “rachischis*” OR “schistorrhach*” OR “spinal dysraphi*” OR “status dysraphicus” OR “thalassemia*” OR “chromosomal triplication*” OR “trisom*”).ti,ab,kw |
| #6 | #4 OR #5 |
| #7 | (MeSH descriptor: [Parents] explode all trees) OR (MeSH descriptor: [Caregivers] explode all trees) OR (MeSH descriptor: [Mothers] explode all trees) OR (MeSH descriptor: [Fathers] explode all trees) OR (MeSH descriptor: [Family] explode all trees) |
| #8 | (“parent*” OR “caregiver*” OR “carer*” OR “mother*” OR “father*” OR “famil*” OR “maternal*” OR “paternal*”).ti,ab,kw |
| #9 | #7 OR #8 |
| #10 | (MeSH descriptor: [Problem Solving] explode all trees) |
| #11 | (“problem-solving” OR “problem solving therap*” OR “problem-solving therap*” OR “problem solving intervention*” OR “problem-solving intervention*” OR “problem solving train*” OR “problem-solving train*” OR “problem solving program*” OR “problem-solving program*” OR “problem solving treatment” OR “problem-solving treatment” OR “problem solving workshop” OR “problem-solving workshop” OR “problem solving education” OR “problem-solving education” OR “problem solving approach” OR “problem-solving approach” OR “problem solving skills train*” OR “problem-solving skills train*” OR “PST” OR “PSST”).ti,ab,kw |
| #12 | #10 OR #11 |
| #13 | (MeSH descriptor: [Clinical Trial] explode all trees) OR (MeSH descriptor: [Controlled Clinical Trial] explode all trees) OR (MeSH descriptor: [Randomized Controlled Trial] explode all trees) OR (MeSH descriptor: [Controlled Clinical Trials as Topic] explode all trees) OR (MeSH descriptor: [Random Allocation] explode all trees) |

© 2024 American Medical Association. All rights reserved.

| | |
|---|---|
| #14 | (“randomized controlled trial*” OR “RCT” OR “random*” OR “trial” OR “clinical trial*” OR “single blind*” OR “double blind*” OR “triple blind*” OR “waitlist*” OR “wait list*” OR “waiting list*”).ti,ab,kw |
| #15 | #13 OR #14 |
| #16 | #3 AND #6 AND #9 AND #12 AND #15 |

**Chinese Databases**

Search date: 30/04/2023

Number of results: CNKI (14), VIP (118), and Wanfang (41)

| **Database** | **Search strategies** |
|---|---|
| China National Knowledge Infrastructure (CNKI) | SU=(儿童 + 孩子 + 小孩 + 患儿 + 小儿 + 未成年人 + 婴儿 + 幼儿 + 青少年) AND SU=(慢性病 + 慢病 + 癌症 + 肿瘤 + 癌 + 瘤 + 白血病 + 血液病 + 贫血 + 关节炎 + 哮喘 + 糖尿病 + 自闭症 + 注意力缺陷 + 多动症 + 脑损伤 + 肾病 + 脑瘫 + 癫痫 + 心脏病 + 镰状细胞病 + 发育障碍 + 发育迟缓 + 唐氏综合征 + 艾滋病 + HIV + AIDS) AND SU=(父母 + 父亲 + 母亲 + 爸爸 + 妈妈 + 家属 + 亲属 + 照护者 + 照顾者) AND SU=(问题解决疗法 + 问题解决干预 + 问题解决培训 + 问题解决技能培训 + 问题解决能力 + 问题解决应对 + 问题解决) |
| China Science and Technology Journal Database (VIP) | “U=(儿童 + 孩子 + 小孩 + 患儿 + 小儿 + 未成年人 + 婴儿 + 幼儿 + 青少年) AND U=(慢性病 + 慢病 + 癌症 + 肿瘤 + 癌 + 瘤 + 白血病 + 血液病 + 贫血 + 关节炎 + 哮喘 + 糖尿病 + 自闭症 + 注意力缺陷 + 多动症 + 脑损伤 + 肾病 + 脑瘫 + 癫痫 + 心脏病 + 镰状细胞病 + 发育障碍 + 发育迟缓 + 唐氏综合征 + 艾滋病 + HIV + AIDS) AND U=(父母 + 父亲 + 母亲 + 爸爸 + 妈妈 + 家属 + 亲属 + 照护者 + 照顾者) AND U=(问题解决疗法 + 问题解决干预 + 问题解决培训 + 问题解决技能培训 + 问题解决能力 + 问题解决应对 + 问题解决)”, 模糊匹配 |
| Wanfang | 主题=(儿童 OR 孩子 OR 小孩 OR 患儿 OR 小儿 OR 未成年人 OR 婴儿 OR 幼儿 OR 青少年) AND 主题=(慢性病 OR 慢病 OR 癌症 OR 肿瘤 OR 癌 OR 瘤 OR 白血病 OR 血液病 OR 贫血 OR 关节炎 OR 哮喘 OR 糖尿病 OR 自闭症 OR 注意力缺陷 OR 多动症 OR 脑损伤 OR 肾病 OR 脑瘫 OR 癫痫 OR 心脏病 OR 镰状细胞病 OR 发育障碍 OR 发育迟缓 OR 唐氏综合征 OR 艾滋病 OR HIV OR AIDS) AND 主题=(父母 OR 父亲 OR 母亲 OR 爸爸 OR 妈妈 OR 家属 OR 亲属 OR 照护者 OR 照顾者) AND 主题=(问题解决疗法 OR 问题解决干预 OR 问题解决培训 OR 问题解决技能培训 OR 问题解决能力 OR 问题解决应对 OR 问题解决) |

© 2024 American Medical Association. All rights reserved.

**eTable 2. Intervention Characteristics of Included Studies**

| Study ID | Intervention Name | Intervention Description | Intervention approach, mode of delivery, duration of delivery, and providers |
|---|---|---|---|
| Askins et al, 2009 | “Bright IDEAS” PSST | Bright represents the sense of optimism (positive orientation) necessary for successful problem-solving. The letters in “IDEAS” signify the five major steps of problem-solving. To promote engagement, the specific problems discussed during the PSST intervention were identified by the individual mother as particularly relevant to her and her family's situation. | Online (personal digital assistant), individual intervention, 8 × 1-h sessions = 8 h over 8 weeks delivered by therapists who had graduate training in psychology. |
| Asnani et al, 2021 | “Bright IDEAS” PSST | The stages of the training are: identification of the problem/s; generating possible solutions; evaluating the options; implementing the preferred solution; and evaluating to see if the solutions were successful. The mothers were taught a process of problem-solving with reference to problems they identified themselves, both general everyday problems as well as specific problems which arose while parenting a child with sickle cell disease. | F2F (in a private room), multi-family group intervention, 6 × 45-min sessions = 4.5 h over 6 months delivered by sickle cell unit clinic nurses. |
| Daniel et al, 2015 | Families Taking Control | The manualized intervention was developed for the current study based on PST. The workshop was divided into four sessions: introduction; applying problem-solving to school challenges; review session and solution generation; goal setting. Following the intervention, the family had approximately three booster phone call sessions to provide support in implementing the problem-solving model by refining goals and trouble-shooting barriers. | F2F (at the hospital) + online (telephone), multi-family group intervention, 1-day workshop (7 h) + 3 × 30-min booster phone calls = 9.5 h over 6 months delivered by doctoral and master’s students and peer patient navigators. |
| DaWalt et al, 2018 | Transitioning Together | Parent intervention group sessions involved education on a variety of topics relevant to autism spectrum disorder as well as guided practice in problem-solving. The adolescent social group involved learning activities and games on topics such as sharing interests, goal setting, problem-solving, and social planning. For both parents and teens, positivity and problem-solving were explicitly modeled and emphasized across all sessions. | F2F (at the hospital or the clinic), multi-family group intervention, 2 × 1-h joining sessions + 8 × 1.5-h group sessions = 14 h over 8 weeks delivered by PhD-level psychologists and graduate students. |

© 2024 American Medical Association. All rights reserved.

**eTable 2. Intervention Characteristics of Included Studies (Continued)**

| Study ID | Intervention Name | Intervention Description | Intervention approach, mode of delivery, duration of delivery, and providers |
|---|---|---|---|
| Feinberg et al, 2014 | Problem-solving Education | A manualized cognitive behavioral intervention adapted from PST. During each workbook-based problem-solving education session, mothers work one-on-one with a trained interventionist to identify a single, measurable problem and then proceed through a series of steps that involve goal setting, brainstorming, and evaluating solutions, choosing a solution, and action planning. | F2F (at home or other location of the mother's choosing), individual intervention, 6 sessions (30-45 mins) = 3-4.5 h over 8 weeks delivered by existing multidisciplinary staff who work with families of young children with ASD. |
| Gerkensmeyer et al, 2013 | Building Our Solutions and Connections | The "Building Our Solutions and Connections" intervention is a cognitive-behavioral PST. During each session, participants learned to enact 7 prescribed steps that guided their problem-solving: (1) evaluating outcomes from the previous week (except for the first week), (2) selecting and defining a problem, (3) establishing realistic and achievable goals for problem resolution, (4) generating multiple solution alternatives (brainstorming), (5) implementing decision-making guidelines (pros and cons), (6) evaluating and choosing solutions, and (7) implementing the caregiver-selected solutions. | F2F (at the hospital) + online (telephone), individual intervention, 1-h beginning session + 8 × 30-min telephone sessions = 5 h over 8 weeks delivered by doctoral and masters clinicians. |
| Greenley et al, 2015 | PSST | The PSST intervention was modeled after the PST outlined by D'Zurilla et al. Families were introduced to the PSST framework and educated about the 5 core problem solving steps (i.e., developing a positive problem outlook, formulating a clear and specific problem definition, brainstorming possible solutions, choosing the best solution, and formulating a solution implementation plan). | Online (telephone), parent-child intervention, 2-4 sessions (session 1: 75 mins, other sessions: 45 mins) = 2-3.5 h over 8 weeks delivered by graduate students enrolled in psychology. |

© 2024 American Medical Association. All rights reserved.

**eTable 2. Intervention Characteristics of Included Studies (Continued)**

| Study ID | Intervention Name | Intervention Description | Intervention approach, mode of delivery, duration of delivery, and providers |
|---|---|---|---|
| McCann et al, 2013 | Problem-Solving Bibliotherapy Intervention | The Problem-Solving Bibliotherapy Intervention group completed a self-help manual, which was based on PST. The intervention involved helping carers to (a) develop a positive attitude to caregiving; (b) identify caregiving-related problems by ascertaining the facts, identifying obstacles that inhibited goal achievement, and setting realistic goals; (c) consider a range of alternatives to circumvent the obstacles and achieve the stated goal; (d) predict positive and negative implications of each alternative in order to choose the one most likely to achieve the problem-solving goal; and (e) try out the solution and monitor if it worked. | F2F (self-help manual) + online (telephone), individual intervention, 5 × 2-h modules = 10 h over 5 weeks with no interventionists reported. |
| Modi et al, 2016 | STAR Intervention | A family-tailored problem-solving intervention, including 1) The interventionist helped identify an adherence barrier (Problem Definition); 2) The caregiver and child were taught to generate creative solutions (Generating Alternative Solutions); 3) The potential solutions were written down and systematically evaluated (Family Decision-Making); 4) The family selected one solution (Implementation of New Solution); 5) A detailed solution was written out with specifics regarding when, where, and how the new solution will be attempted. A behavioral contract was signed by all participants of the problem-solving session; and 6) Phone follow-ups were conducted one week after the problem-solving session to assist the family in either fine-tuning the solution or renegotiating a new solution (Evaluation and Re-Negotiation). | F2F (at the clinic or home) + online (telephone), parent-child intervention, 4 × 1-h F2F sessions + 2 × 15-min telephone sessions = 4.5 h over 8 weeks delivered by psychology doctoral student and post-doctoral fellow. |
| Modi et al, 2021 | STAR Intervention | A problem-solving approach to address the family's individualized adherence barriers, including 1) Identification of the adherence barrier experienced by the family, 2) Generation of 8–10 creative solutions by all family members involved in the session, 3) Evaluation of the solutions by family members, with each member rating the solution as + or −, 4) Choice of one or two combined solutions to implement, and 5) Provision of detailed information on how the solution will be implemented (who, what, when, where, and how). | F2F (at the clinic or home) + online (telephone), parent-child intervention, 6 × 1-h F2F sessions + 2 × 15-min telephone sessions = 6.5 h over 4 months delivered by master's and doctoral level psychologists/trainees. |

© 2024 American Medical Association. All rights reserved.

**eTable 2. Intervention Characteristics of Included Studies (Continued)**

| Study ID | Intervention Name | Intervention Description | Intervention approach, mode of delivery, duration of delivery, and providers |
|---|---|---|---|
| Nansel et al, 2009 | WE*CAN Intervention | Based on PST, the WE*CAN structure was developed: W - Work together to set goals; E - Explore possible barriers and solutions; C - Choose the best solutions; A - Act on your plan; N - Note the results. The goal of WE*CAN is to improve family management of diabetes, including domains of blood sugar monitoring, insulin administration, diet, physical activity, and management of blood sugar excursions. | F2F (at routine clinic visits) + online (telephone), parent-child intervention, 3 sessions + 9 phone calls over a maximum of 12 months delivered by health advisors (college graduates). |
| Narad et al, 2019 | TOPS-Family | A web-based, family-centered problem-solving intervention designed to support adolescent and family outcomes following pediatric TBI. The intervention addressed common challenges following TBI by providing psychoeducation as well as teaching adolescents and their parents a 5-step problem solving process (Aim, Brainstorm, Choose, Do, and Evaluate) that was generalizable to concerns beyond those addressed during the study. This problem-solving framework was grounded in PST model. | Online (web-based didactic modules + videoconference), parent-child intervention, 10 core sessions + max of 4 supplemental sessions over 6 months, delivered by advanced clinical psychology graduate students or licensed clinical psychologists. |
| Palermo et al, 2016 | "Bright IDEAS" PSST | The intervention was adapted from treatment materials for caregivers of children with cancer ("Bright IDEAS"). Drawing from D'Zurilla and colleagues' conceptualization of PST, "Bright IDEAS" emphasizes a positive problem-solving orientation characterized by optimism and problem-solving self-efficacy (Bright), as well as the major components of rational problem solving. These include problem definition and formulation (Identify the Problem), generation of alternative solutions (Determine the options), decision-making (Evaluate options), solution implementation (Act), and verification (See if it worked). | F2F (in pain clinic), individual intervention, 4-6 × 1-h sessions = 4-6 h over 6-8 weeks, delivered by psychology postdoctoral fellows and licensed clinical psychologists. |

© 2024 American Medical Association. All rights reserved.

**eTable 2. Intervention Characteristics of Included Studies (Continued)**

| Study ID | Intervention Name | Intervention Description | Intervention approach, mode of delivery, duration of delivery, and providers |
|---|---|---|---|
| Petranovich et al, 2015 | Counselor-Assisted Problem Solving | A family problem-solving intervention based on PST that provided training in communication skills, self-regulation and anger management. The core sessions primarily focused on problem-solving and application to a goal or problem identified by the family. After generating a solution to the goal or problem that the family initially identified as problematic, the family's homework was to implement the agreed-upon plan and evaluate its success. Subsequent core sessions focused on developing strategies to facilitate effective family problem-solving, including basic communication skills and developing strategies to address common cognitive and behavioral consequences of TBI. | Online (web-based didactic modules + videoconference), parent-child intervention, 7 core sessions (45–60 minutes) + max of 4 supplemental sessions over 6 months, delivered by clinical psychologists. |
| Phipps et al, 2020 | "Bright IDEAS" PSST | The term "Bright" signifies optimism, and instills the belief that problems can be solved, which is considered an essential component for successful implementation of the intervention. The acronym 'IDEAS' is used as a mnemonic for the 5 essential steps of our problem-solving approach, with each letter signifying a step: I (Identify the problem), D (Determine the options), E (Evaluate/choose the best option), A (Act), and S (See if it worked). | Online (web-based videos, interactive activities, and homework), individual intervention, 7 videos + 4 photos over 8 weeks, delivered by research assistants with graduate education in clinical psychology. |
| Sahler et al, 2002 | "Bright IDEAS" PSST | 'Bright'' signifies the sense of optimism (positive orientation) about solving problems that is essential for successful implementation. The letters I (Identify the problem), D (Determine the options), E (Evaluate options and choose the best), A (Act), and S (See if it worked) signify the five essential steps of PST as articulated by D'Zurilla and Nezu. | F2F (at the hospital, the clinic, or home), individual intervention, 8 × 1-h sessions = 8 h over 8 weeks, delivered by master-level mental health professional or psychology graduate student. |

© 2024 American Medical Association. All rights reserved.

**eTable 2. Intervention Characteristics of Included Studies (Continued)**

| Study ID | Intervention Name | Intervention Description | Intervention approach, mode of delivery, duration of delivery, and providers |
|---|---|---|---|
| Sahler et al, 2005 | “Bright IDEAS” PSST | Bright signifies the sense of optimism (positive orientation) about solving problems that is essential for successful implementation. The letters I (identify the problem), D (determine the options), E (evaluate options and choose the best), A (act), and S (see if it worked) signify the five essential steps of PST. | F2F (at the hospital, the clinic, or home), individual intervention, 8 × 1-h sessions = 8 h over 8 weeks, delivered by master-level mental health professional or psychology graduate student. |
| Sahler et al, 2013 | “Bright IDEAS” PSST | “Bright” signifies the optimism about solving problems essential for successful implementation. The letters I (identify the problem), D (determine the options), E (evaluate options/choose the best), A (act), and S (see if it worked) signify the five essential steps of PST. | F2F (at the hospital, the clinic, or home), individual intervention, 8×1 h sessions over 8 weeks= 8 h, delivered by research assistants who had graduate education in clinical psychology or behavioral health. |
| Seid et al, 2010 | PSST | PSST is a generic psychoeducational approach in which problems are normalized and participants are taught to approach problems proactively, define the problem, generate alternative solutions, choose the best solution, implement the solution, and evaluate how well that solution worked. The intervention was based on PST and adapted from a comprehensive protocol used in mothers of children with cancer. | F2F (at home), parent-child intervention, 6 weekly sessions (45-60 mins) = 4.5-6 h over 6 weeks, delivered by a bilingual, bicultural master's level health educator. |
| Wade et al, 2006a | FPS | The family problem-solving intervention, adapted from D’Zurilla and Nezu, involved a 5-step process: Aim, Brainstorm, Choose, Do It, and Evaluate—ABCDE for short. Prior to introducing the problem-solving process, the therapist emphasized the importance of having a positive attitude toward problem solving, which was referred to as a positive problem orientation. | F2F (at the clinic or the family’s home), parent-child intervention, 7 core sessions + max of 4 individualized sessions (each session 75-100 mins) over 6 months, delivered by a 5th year clinical psychology graduate student. |

© 2024 American Medical Association. All rights reserved.

**eTable 2. Intervention Characteristics of Included Studies (Continued)**

| Study ID | Intervention Name | Intervention Description | Intervention approach, mode of delivery, duration of delivery, and providers |
|---|---|---|---|
| Wade et al, 2006b | Online-FPS | The Family Problem-Solving Therapy had 14 separate sessions. Eight "core" sessions provided training in problem solving, communication, and antecedent behavior management skills for all enrolled families, whereas the remaining six sessions addressed content related to stressors or issues that affected some but not all families. The first two Web-based sessions addressed the importance of having a positive problem orientation and the steps of problem solving (Aim, Brainstorm, Choose, Do It, and Evaluate; ABCDE). | Online (web-based didactic modules + teleconference), parent-child intervention, 8 core sessions + 6 supplemental sessions over 6 months, delivered by clinical psychology graduate student. |
| Wade et al, 2012 | TOPS | The TOPS intervention focused on developing a positive approach to problems and the steps of problem-solving. The problem-solving heuristic consisted of five steps: Aim (goal identification), Brainstorm (solution generation), Choose (evaluation of the various solutions), Do It (detailed implementation plan), and Evaluate (did it work). | Online (web-based didactic modules + teleconference), parent-child intervention, 10 core sessions + max of 4 supplemental sessions (each session 45-60 minutes) over 6 months, delivered by a staff psychologist and doctoral students in clinical psychology. |
| Wade et al, 2019 | FPS | Treatment content was equivalent across treatment arms, though groups differed in terms of delivery mode (online vs face to face) and degree of therapist involvement (therapist-guided vs self-guided). Participants and their families in all 3 treatment arms could complete up to 10 sequential sessions providing training in staying positive/cognitive reframing, problem-solving, and communication. | (a) Online (therapist-guided, web-based didactic modules + videoconference), parent-child intervention, 10 core sessions+ max of 4 supplemental sessions (each session 60 minutes) over 6 months, delivered by licensed psychologists and postdoctoral fellows in psychology.<br>(b) Online (self-guided, web-based didactic modules), parent-child intervention, 10 core sessions+ all 11 supplemental sessions (each session 60 minutes) over 6 months, with no interventionist. |

**Abbreviations:** ASD, autism spectrum disorder; FPS, family problem-solving therapy; F2F, face-to-face; PSST, problem-solving skills training; PST, problem-solving therapy; STAR, supporting treatment adherence regimens; TBI, traumatic brain injury; TOPS, teen online problem-solving.

© 2024 American Medical Association. All rights reserved.

**eTable 3. Author Judgments of Risk of Bias Across All Included Studies**

| Study ID | Domain 1. Randomization process | | | | Domain 2. Deviations from intended interventions | | | | | | | |
|---|---|---|---|---|---|---|---|---|---|---|---|---|
| | 1.1 | 1.2 | 1.3 | Judgement | 2.1 | 2.2 | 2.3 | 2.4 | 2.5 | 2.6 | 2.7 | Judgement |
| Askins et al, 2009 | Y | NI | N | Some concerns | PY | PY | N | NA | NA | Y | NA | Low |
| Asnani et al, 2021 | Y | Y | N | Low | PY | PY | N | NA | NA | Y | NA | Low |
| Daniel et al, 2015 | NI | PY | N | Low | Y | Y | PN | NA | NA | Y | NA | Low |
| DaWalt et al, 2018 | NI | NI | PY | High | PY | PY | PN | NA | NA | PN | PN | Some concerns |
| Feinberg et al, 2014 | Y | Y | PN | Low | PY | PY | PN | NA | NA | PY | NA | Low |
| Gerkensmeyer et al, 2013 | NI | NI | N | Some concerns | PY | PY | PN | NA | NA | NI | PN | Some concerns |
| Greenley et al, 2015 | Y | Y | PN | Low | PY | PY | N | NA | NA | PN | PN | Some concerns |
| McCann et al, 2013 | Y | Y | N | Low | PY | PY | N | NA | NA | Y | NA | Low |
| Modi et al, 2016 | Y | NI | NI | Some concerns | NI | NI | N | NA | NA | PY | NA | Low |
| Modi et al, 2021 | Y | Y | N | Low | PY | N | N | NA | NA | Y | NA | Low |
| Nansel et al, 2009 | NI | PN | N | High | PY | PY | PN | NA | NA | NI | PN | Some concerns |
| Narad et al, 2019 | Y | Y | N | Low | PY | PY | N | NA | NA | Y | NA | Low |
| Palermo et al, 2016 | Y | Y | N | Low | Y | Y | N | NA | NA | Y | NA | Low |
| Petranovich et al, 2015 | Y | Y | N | Low | PY | PY | N | NA | NA | Y | NA | Low |
| Phipps et al, 2020 | PY | NI | N | Some concerns | PY | PY | PN | NA | NA | Y | NA | Low |
| Sahler et al, 2002 | PY | PY | N | Low | PY | PY | NI | NA | NA | Y | NA | Some concerns |
| Sahler et al, 2005 | PY | NI | N | Some concerns | PY | PY | PN | NA | NA | PY | NA | Low |
| Sahler et al, 2013 | PY | NI | N | Some concerns | PY | PY | PN | NA | NA | Y | NA | Low |
| Seid et al, 2010 | PY | PY | N | Low | PY | PY | N | NA | NA | Y | NA | Low |
| Wade et al, 2006a | PY | NI | N | Some concerns | PY | PY | PN | NA | NA | NI | PN | Some concerns |
| Wade et al, 2006b | Y | NI | N | Some concerns | Y | Y | PN | NA | NA | PY | NA | Low |
| Wade et al, 2012 | PY | NI | N | Some concerns | Y | Y | PN | NA | NA | NI | PN | Some concerns |
| Wade et al, 2019 | Y | Y | PN | Low | PN | PY | PN | NA | NA | NI | PN | Some concerns |

© 2024 American Medical Association. All rights reserved.

## eTable 3. Author Judgments of Risk of Bias Across All Included Studies (Continued)

| Study ID | Domain 3. Missing outcome data | | | | | Domain 4. Measurement of the outcome | | | | | |
|---|---|---|---|---|---|---|---|---|---|---|---|
| | 3.1 | 3.2 | 3.3 | 3.4 | Judgement | 4.1 | 4.2 | 4.3 | 4.4 | 4.5 | Judgement |
| Askins et al, 2009 | N | PN | PY | PN | Some concerns | N | N | PN | NA | NA | Low |
| Asnani et al, 2021 | N | PN | PN | NA | Low | N | N | N | NA | NA | Low |
| Daniel et al, 2015 | N | N | NI | PN | Some concerns | N | PN | PY | PY | PN | Some concerns |
| DaWalt et al, 2018 | N | PN | PN | NA | Low | N | N | NI | PY | PN | Some concerns |
| Feinberg et al, 2014 | N | PN | PN | NA | Low | N | N | N | NA | NA | Low |
| Gerkensmeyer et al, 2013 | N | PN | PN | NA | Low | N | N | PY | PY | PY | High |
| Greenley et al, 2015 | N | PN | N | NA | Low | N | N | PN | NA | NA | Low |
| McCann et al, 2013 | N | N | PN | NA | Low | N | N | N | NA | NA | Low |
| Modi et al, 2016 | N | N | NI | PN | Some concerns | N | N | NI | NI | PN | Some concerns |
| Modi et al, 2021 | N | Y | NA | NA | Low | N | N | N | NA | NA | Low |
| Nansel et al, 2009 | PN | PN | NI | NI | High | N | N | PN | NA | NA | Low |
| Narad et al, 2019 | N | PN | PN | NA | Low | N | N | PY | PN | NA | Low |
| Palermo et al, 2016 | Y | NA | NA | NA | Low | N | N | N | NA | NA | Low |
| Petranovich et al, 2015 | PN | PY | NA | NA | Low | N | N | N | NA | NA | Low |
| Phipps et al, 2020 | N | PY | NA | NA | Low | N | N | NI | PN | NA | Low |
| Sahler et al, 2002 | NI | PN | NI | NI | Some concerns | N | N | NI | PY | PN | Some concerns |
| Sahler et al, 2005 | N | PN | NI | PN | Some concerns | N | N | NI | PY | PN | Some concerns |
| Sahler et al, 2013 | PN | PN | NI | PN | Some concerns | N | N | N | NA | NA | Low |
| Seid et al, 2010 | N | PY | NA | NA | Low | N | N | N | NA | NA | Low |
| Wade et al, 2006a | PN | PN | PY | PN | Some concerns | N | N | NI | NI | PN | Some concerns |
| Wade et al, 2006b | PN | PY | NA | NA | Low | N | N | PY | PN | NA | Low |
| Wade et al, 2012 | N | N | PY | PY | High | N | N | Y | PN | NA | Low |
| Wade et al, 2019 | N | PN | PN | NA | Low | N | N | PN | NA | NA | Low |

© 2024 American Medical Association. All rights reserved.

## eTable 3. Author Judgments of Risk of Bias Across All Included Studies (Continued)

| Study ID | Domain 5. Selection of the reported result | | | | Overall |
|---|---|---|---|---|---|
| | 1.1 | 1.2 | 1.3 | Judgement | |
| Askins et al, 2009 | Y | PN | PY | High | High[c] |
| Asnani et al, 2021 | Y | N | N | Low | Low[a] |
| Daniel et al, 2015 | NI | PN | PN | Some concerns | Some concerns[b] |
| DaWalt et al, 2018 | PY | N | N | Low | High |
| Feinberg et al, 2014 | Y | N | N | Low | Low |
| Gerkensmeyer et al, 2013 | PY | PN | PN | Low | High |
| Greenley et al, 2015 | Y | N | N | Low | Some concerns |
| McCann et al, 2013 | Y | N | N | Low | Low |
| Modi et al, 2016 | Y | N | N | Low | Some concerns |
| Modi et al, 2021 | Y | N | N | Low | Low |
| Nansel et al, 2009 | PY | PN | PN | Low | High |
| Narad et al, 2019 | PY | PY | PY | High | High |
| Palermo et al, 2016 | Y | N | N | Low | Low |
| Petranovich et al, 2015 | Y | N | N | Low | Low |
| Phipps et al, 2020 | Y | N | N | Low | Some concerns |
| Sahler et al, 2002 | Y | N | PY | High | High |
| Sahler et al, 2005 | Y | N | PY | High | High |
| Sahler et al, 2013 | Y | N | N | Low | Some concerns |
| Seid et al, 2010 | Y | N | N | Low | Low |
| Wade et al, 2006a | PY | N | N | Low | Some concerns |
| Wade et al, 2006b | PY | PN | PN | Low | Some concerns |
| Wade et al, 2012 | PY | PN | PY | High | High |
| Wade et al, 2019 | PY | PN | PN | Low | Some concerns |

**Abbreviations:** Y, Yes; PY, Probably yes; PN, Probably no; N, No; NI, No information; NA, Not applicable.

[a] Low risk of bias: The study is judged to be at low risk of bias for all domains for this result.

[b] Some concerns: The study is judged to be at some concerns in at least one domain for this result.

[c] High risk of bias: The study is judged to be at high risk of bias in at least one domain for this result or the study is judged to have some concerns for multiple domains in a way that substantially lowers confidence in the result.

© 2024 American Medical Association. All rights reserved.

**eTable 4.Grading of Recommendations Assessment, Development, and Evaluation (GRADE) Evidence Profile**

| Outcomes | Risk of bias[a] | Inconsistency[b] | Indirectness[c] | Imprecision[d] | Publication bias[e] | Certainty of the evidence (GRADE)[f] |
|---|---|---|---|---|---|---|
| **Parental psychosocial outcome** | | | | | | |
| Depression | Not serious | Serious | Not serious | Not serious | Not serious | ⊕⊕⊕◯ Moderate |
| Distress | Not serious | Serious | Not serious | Not serious | Not serious | ⊕⊕⊕◯ Moderate |
| Problem-solving skills | Not serious | Serious | Not serious | Not serious | Not serious | ⊕⊕⊕◯ Moderate |
| Post-traumatic stress | Serious | Not serious | Not serious | Not serious | Not serious | ⊕⊕⊕◯ Moderate |
| Parenting stress | Not serious | Serious | Not serious | Serious | Not serious | ⊕⊕◯◯ Low |
| Anxiety | Not serious | Serious | Not serious | Very serious | Not serious | ⊕◯◯◯ Very low |
| Parental quality of Life | Not serious | Not serious | Not serious | Very serious | Not serious | ⊕⊕◯◯ Low |
| **Pediatric psychosocial outcome** | | | | | | |
| Pediatric quality of life | Not serious | Serious | Not serious | Serious | Not serious | ⊕⊕◯◯ Low |
| Mental problems | Not serious | Not serious | Not serious | Serious | Not serious | ⊕⊕⊕◯ Moderate |
| Social functioning | Not serious | Serious | Not serious | Very serious | Not serious | ⊕◯◯◯ Very low |
| **Family psychosocial outcome** | | | | | | |
| Parent-child conflict | Serious | Not serious | Not serious | Serious | Not serious | ⊕⊕◯◯ Low |

[a] Risk-of-bias: certainty was downgraded if more than 50% of the weights of individual RCTs in each outcome assessed came from high-risk studies.

[b] Inconsistency: based on the variability and heterogeneity across individual trials. Certainty was downgraded if $I^2 > 50\%$ and/or if the p-value of the heterogeneity test was < .05.

[c] Indirectness: assessed qualitatively by the extent to which the population, interventions, and outcome measures directly reflect the aims of the systematic review.

[d] Imprecision: based on inspection of the pooled estimate and the 95% confidence interval (95%CIs). We decreased the grade rating by one (-1) when the analysis included fewer than 500 participants or if there were wide confidence intervals, and by two (-2) when the number of participants included in the analysis was very low or if confidence intervals were very wide.

[e] Publication bias: assessed by a funnel plot and an extension to Egger's regression test. Certainty downgraded when p < 0.05.

[f] High certainty: we are very confident that the true effect lies close to that of the estimate of the effect; Moderate certainty: we are moderately confident in the effect estimate: the true effect is likely to be close to the estimate of the effect, but there is a possibility that it is substantially different; Low certainty: our confidence in the effect estimate is limited: the true effect may be substantially different from the estimate of the effect; Very low certainty: we have very little confidence in the effect estimate: the true effect is likely to be substantially different from the estimate of effect.

© 2024 American Medical Association. All rights reserved.

# eFigure 1. Forest Plot Meta-Analyses for Different Psychosocial Outcomes

## eFigure 1.1. Forest Plot of PSST Association with Parental Depression

| Study | Treatment N | Treatment Mean | Treatment SD | Control N | Control Mean | Control SD | Hedges's g with 95% CI | Weight (%) |
|---|---|---|---|---|---|---|---|---|
| Askins 2009 (a) | 66 | 3.34 | 1.41 | 93 | 3.94 | 0.68 | -0.57 [ -0.89, -0.25] | 6.88 |
| Askins 2009 (b) | 65 | 3.24 | 1.40 | 104 | 3.94 | 0.71 | -0.68 [ -0.99, -0.36] | 6.91 |
| Asnani 2021 | 32 | 14.00 | 10.30 | 32 | 15.70 | 12.30 | -0.15 [ -0.63, 0.34] | 5.76 |
| DaWalt 2018 | 19 | -5.44 | 3.01 | 26 | 0.11 | 2.38 | -2.05 [ -2.77, -1.33] | 4.27 |
| Feinberg 2014 | 53 | 4.60 | 3.50 | 58 | 6.90 | 5.00 | -0.53 [ -0.90, -0.15] | 6.51 |
| Palermo 2016 | 31 | 7.87 | 5.82 | 30 | 9.33 | 8.51 | -0.20 [ -0.70, 0.30] | 5.67 |
| Petranovich 2015 | 61 | 11.10 | 9.30 | 64 | 15.40 | 11.70 | -0.40 [ -0.76, -0.05] | 6.67 |
| Phipps 2020 (a) | 244 | 5.15 | 7.37 | 310 | 7.51 | 5.55 | -0.37 [ -0.54, -0.20] | 7.73 |
| Phipps 2020 (b) | 244 | 5.15 | 7.37 | 311 | 8.14 | 5.67 | -0.46 [ -0.63, -0.29] | 7.73 |
| Sahler 2005 | 191 | -0.73 | 1.66 | 195 | -0.17 | 1.66 | -0.34 [ -0.54, -0.14] | 7.58 |
| Sahler 2013 | 96 | 3.25 | 1.27 | 108 | 3.27 | 1.35 | -0.02 [ -0.29, 0.26] | 7.18 |
| Wade 2006a | 16 | 52.62 | 10.24 | 16 | 56.19 | 12.59 | -0.30 [ -0.98, 0.38] | 4.50 |
| Wade 2006b | 20 | 9.25 | 7.09 | 20 | 18.15 | 13.49 | -0.81 [ -1.44, -0.18] | 4.78 |
| Wade 2019 (a) | 22 | 15.10 | 1.81 | 34 | 14.20 | 1.73 | 0.50 [ -0.03, 1.04] | 5.40 |
| Wade 2019 (b) | 43 | 13.50 | 1.42 | 56 | 12.80 | 1.49 | 0.48 [ 0.08, 0.88] | 6.35 |
| Wade 2019 (c) | 51 | 12.80 | 1.53 | 60 | 15.50 | 1.56 | -1.73 [ -2.17, -1.30] | 6.10 |
| **Overall** | | | | | | | -0.45 [ -0.66, -0.23] | |

Heterogeneity: $\tau^2 = 0.15$, $I^2 = 85.29\%$, $H^2 = 6.80$

Test of $\theta_i = \theta_j$: $Q(15) = 101.99$, $p = 0.00$

Test of $\theta = 0$: $z = -4.05$, $p = 0.00$

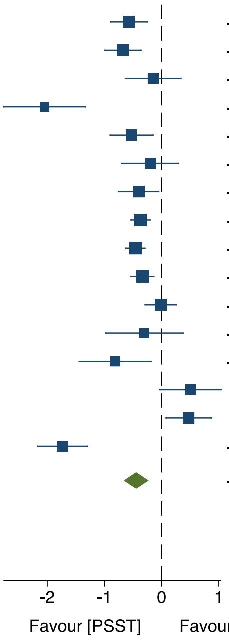


Random-effects DerSimonian-Laird model

## eFigure 1.2. Forest Plot of PSST Association with Parental Distress

| Study | Treatment N | Treatment Mean | Treatment SD | Control N | Control Mean | Control SD | Hedges's g with 95% CI | Weight (%) |
|---|---|---|---|---|---|---|---|---|
| Askins 2009 (a) | 66 | 33.70 | 35.98 | 93 | 55.70 | 22.76 | -0.76 [ -1.08, -0.43] | 6.88 |
| Askins 2009 (b) | 65 | 34.60 | 35.08 | 104 | 55.70 | 24.07 | -0.73 [ -1.05, -0.41] | 6.93 |
| McCann 2013 | 56 | 19.90 | 1.20 | 58 | 21.80 | 1.20 | -1.57 [ -1.99, -1.15] | 6.20 |
| Palermo 2016 | 31 | 29.35 | 33.60 | 30 | 26.87 | 36.87 | 0.07 [ -0.43, 0.57] | 5.62 |
| Petranovich 2015 | 61 | 47.80 | 12.50 | 64 | 53.90 | 12.00 | -0.50 [ -0.85, -0.14] | 6.67 |
| Phipps 2020 (a) | 244 | 86.17 | 42.38 | 310 | 99.17 | 42.18 | -0.31 [ -0.48, -0.14] | 7.85 |
| Phipps 2020 (b) | 244 | 84.44 | 41.79 | 311 | 104.24 | 42.86 | -0.47 [ -0.64, -0.30] | 7.84 |
| Sahler 2002 | 33 | 80.76 | 38.81 | 40 | 98.10 | 48.50 | -0.39 [ -0.85, 0.07] | 5.88 |
| Sahler 2005 | 191 | -19.76 | 38.75 | 195 | -6.68 | 38.75 | -0.34 [ -0.54, -0.14] | 7.68 |
| Sahler 2013 | 96 | 33.60 | 35.86 | 108 | 38.30 | 36.37 | -0.13 [ -0.40, 0.14] | 7.23 |
| Wade 2006a | 16 | 53.13 | 12.03 | 16 | 55.13 | 14.09 | -0.15 [ -0.83, 0.53] | 4.41 |
| Wade 2006b | 20 | 52.33 | 10.69 | 20 | 58.37 | 11.49 | -0.53 [ -1.15, 0.09] | 4.77 |
| Wade 2012 | 16 | 48.85 | 7.92 | 19 | 54.03 | 9.42 | -0.58 [ -1.24, 0.09] | 4.48 |
| Wade 2019 (a) | 22 | 56.10 | 2.46 | 34 | 56.90 | 1.93 | -0.37 [ -0.90, 0.17] | 5.35 |
| Wade 2019 (b) | 43 | 54.70 | 1.88 | 56 | 56.90 | 1.69 | -1.23 [ -1.66, -0.80] | 6.11 |
| Wade 2019 (c) | 51 | 53.30 | 2.02 | 60 | 56.60 | 1.77 | -1.74 [ -2.17, -1.30] | 6.07 |
| **Overall** | | | | | | | -0.61 [ -0.81, -0.40] | |

Heterogeneity: $\tau^2 = 0.14$, $I^2 = 83.88\%$, $H^2 = 6.20$

Test of $\theta_i = \theta_j$: $Q(15) = 93.05$, $p = 0.00$

Test of $\theta = 0$: $z = -5.72$, $p = 0.00$

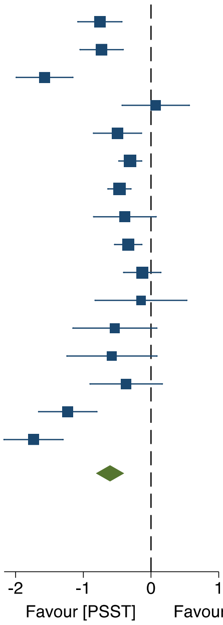


Random-effects DerSimonian-Laird model

© 2024 American Medical Association. All rights reserved.

## eFigure 1.3. Forest Plot of PSST Association with Parental Problem-solving Skills

| Study | Treatment N | Treatment Mean | Treatment SD | Control N | Control Mean | Control SD | Hedges's g with 95% CI | Weight (%) |
|---|---|---|---|---|---|---|---|---|
| Askins 2009 (a) | 66 | 14.60 | 2.06 | 93 | 13.30 | 1.45 | 0.75 [ 0.42, 1.07] | 8.31 |
| Askins 2009 (b) | 65 | 14.50 | 2.01 | 104 | 13.30 | 1.53 | 0.69 [ 0.37, 1.01] | 8.45 |
| Asnani 2021 | 32 | 93.60 | 10.60 | 32 | 91.60 | 9.80 | 0.19 [ -0.29, 0.68] | 5.66 |
| Daniel 2015 | 28 | 111.81 | 11.48 | 41 | 106.71 | 14.17 | 0.38 [ -0.10, 0.86] | 5.74 |
| DaWalt 2018 | 16 | 0.44 | 0.22 | 25 | 0.02 | 0.18 | 2.10 [ 1.33, 2.86] | 3.05 |
| Feinberg 2014 | 53 | 17.89 | 3.51 | 57 | 17.65 | 4.17 | 0.06 [ -0.31, 0.43] | 7.43 |
| Modi 2016 | 9 | 114.40 | 12.80 | 12 | 107.10 | 10.90 | 0.60 [ -0.25, 1.45] | 2.58 |
| Palermo 2016 | 31 | 14.73 | 2.00 | 30 | 13.75 | 2.39 | 0.44 [ -0.06, 0.94] | 5.44 |
| Phipps 2020 (a) | 244 | 14.43 | 2.80 | 310 | 13.77 | 2.63 | 0.24 [ 0.08, 0.41] | 11.49 |
| Phipps 2020 (b) | 244 | 14.68 | 2.82 | 311 | 13.59 | 2.64 | 0.40 [ 0.23, 0.57] | 11.48 |
| Sahler 2002 | 33 | 74.58 | 14.48 | 40 | 70.68 | 13.49 | 0.28 [ -0.18, 0.73] | 6.03 |
| Sahler 2005 | 191 | 0.46 | 1.84 | 195 | 0.01 | 1.84 | 0.24 [ 0.04, 0.44] | 10.86 |
| Sahler 2013 | 96 | 14.40 | 2.16 | 108 | 13.70 | 2.18 | 0.32 [ 0.05, 0.60] | 9.29 |
| Wade 2006b | 20 | 73.45 | 9.61 | 20 | 69.16 | 10.02 | 0.43 [ -0.19, 1.04] | 4.19 |
| **Overall** | | | | | | | 0.43 [ 0.27, 0.58] | |

Heterogeneity: $\tau^2 = 0.04$, $I^2 = 64.28\%$, $H^2 = 2.80$

Test of $\theta_i = \theta_j$: $Q(13) = 36.39$, $p = 0.00$

Test of $\theta = 0$: $z = 5.50$, $p = 0.00$

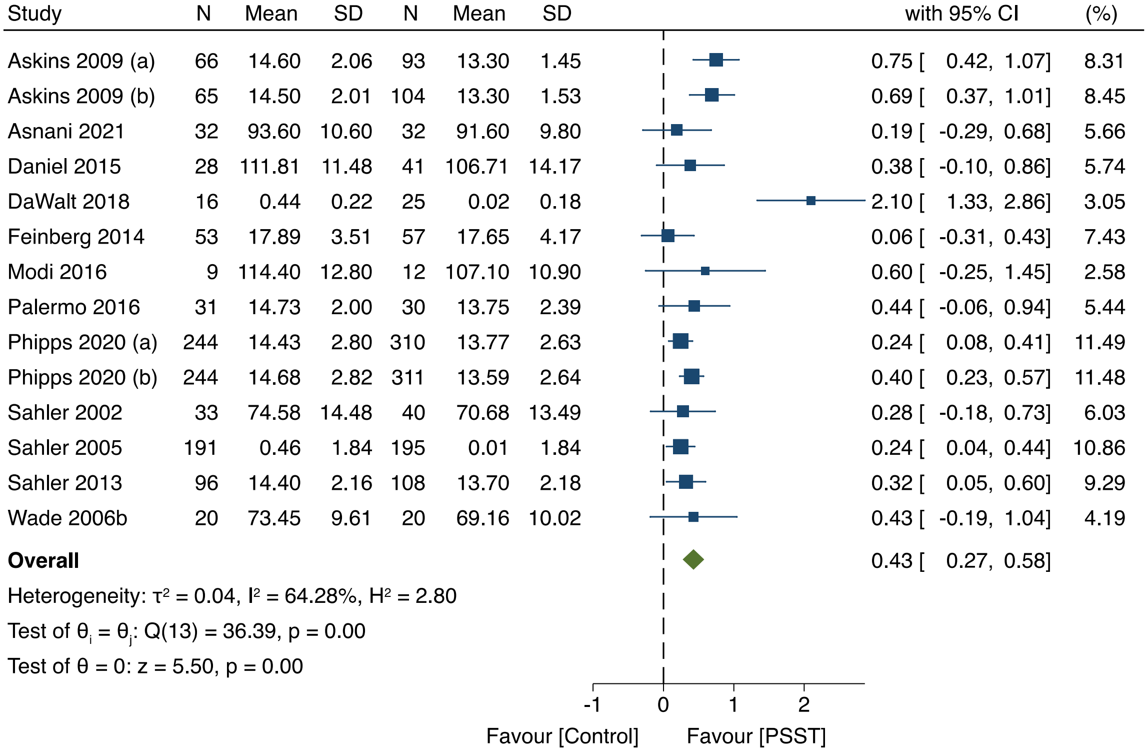


Random-effects DerSimonian-Laird model

## eFigure 1.4. Forest Plot of PSST Association with Parental Post-traumatic Stress

| Study | Treatment N | Treatment Mean | Treatment SD | Control N | Control Mean | Control SD | Hedges's g with 95% CI | Weight (%) |
|---|---|---|---|---|---|---|---|---|
| Askins 2009 (a) | 66 | 26.00 | 15.98 | 93 | 35.50 | 10.32 | -0.73 [ -1.05, -0.41] | 7.23 |
| Askins 2009 (b) | 65 | 29.60 | 15.68 | 104 | 35.50 | 10.91 | -0.45 [ -0.77, -0.14] | 7.78 |
| Palermo 2016 | 31 | 32.65 | 8.67 | 30 | 34.79 | 8.44 | -0.25 [ -0.74, 0.25] | 3.07 |
| Phipps 2020 (a) | 244 | 23.10 | 21.98 | 310 | 30.18 | 17.59 | -0.36 [ -0.53, -0.19] | 26.63 |
| Phipps 2020 (b) | 244 | 20.85 | 22.06 | 311 | 30.45 | 17.35 | -0.49 [ -0.66, -0.32] | 26.31 |
| Sahler 2005 | 191 | -9.24 | 15.64 | 195 | -3.96 | 15.64 | -0.34 [ -0.54, -0.14] | 18.87 |
| Sahler 2013 | 96 | 28.20 | 15.87 | 108 | 29.70 | 16.00 | -0.09 [ -0.37, 0.18] | 10.11 |
| **Overall** | | | | | | | -0.39 [ -0.48, -0.31] | |

Heterogeneity: $I^2 = 44.93\%$, $H^2 = 1.82$

Test of $\theta_i = \theta_j$: $Q(6) = 10.89$, $p = 0.09$

Test of $\theta = 0$: $z = -8.85$, $p = 0.00$

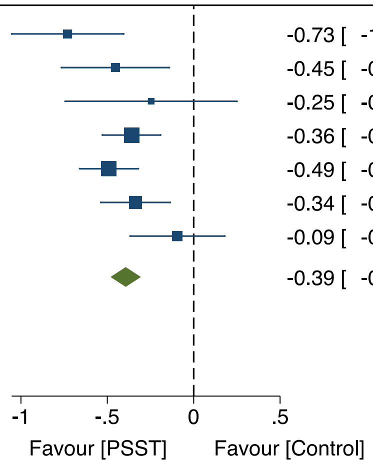


Fixed-effects inverse-variance model

© 2024 American Medical Association. All rights reserved.

## eFigure 1.5. Forest Plot of PSST Association with Parenting Stress

| Study | Treatment N | Treatment Mean | Treatment SD | Control N | Control Mean | Control SD | Hedges's g with 95% CI | Weight (%) |
|---|---|---|---|---|---|---|---|---|
| Asnani 2021 | 32 | -6.30 | 18.66 | 32 | 3.30 | 18.17 | -0.51 [ -1.01, -0.02] | 19.73 |
| DaWalt 2018 | 16 | -2.49 | 2.25 | 25 | -1.22 | 1.77 | -0.63 [ -1.26, -0.00] | 16.91 |
| Feinberg 2014 | 53 | 85.50 | 19.60 | 58 | 94.20 | 25.60 | -0.38 [ -0.75, -0.00] | 22.18 |
| McCann 2013 | 56 | 83.90 | 4.80 | 58 | 90.30 | 4.70 | -1.34 [ -1.74, -0.93] | 21.56 |
| Palermo 2016 | 31 | 78.29 | 23.70 | 30 | 82.43 | 19.71 | -0.19 [ -0.68, 0.31] | 19.63 |
| **Overall** | | | | | | | -0.62 [ -1.05, -0.19] | |

Heterogeneity: $\tau^2 = 0.18$, $I^2 = 76.24\%$, $H^2 = 4.21$

Test of $\theta_i = \theta_j$: Q(4) = 16.84, p = 0.00

Test of $\theta = 0$: z = -2.82, p = 0.00

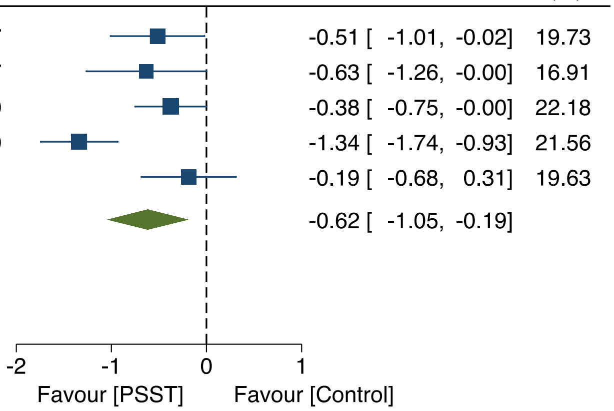


Random-effects DerSimonian-Laird model

## eFigure 1.6. Forest Plot of PSST Association with Parental Anxiety

| Study | Treatment N | Treatment Mean | Treatment SD | Control N | Control Mean | Control SD | Hedges's g with 95% CI | Weight (%) |
|---|---|---|---|---|---|---|---|---|
| Palermo 2016 | 31 | 7.48 | 4.44 | 30 | 6.40 | 4.64 | 0.23 [ -0.26, 0.73] | 37.45 |
| Wade 2006a | 16 | 50.38 | 11.44 | 16 | 53.14 | 14.09 | -0.21 [ -0.89, 0.47] | 30.36 |
| Wade 2006b | 20 | 9.25 | 4.99 | 20 | 14.05 | 7.50 | -0.74 [ -1.37, -0.11] | 32.19 |
| **Overall** | | | | | | | -0.21 [ -0.80, 0.37] | |

Heterogeneity: $\tau^2 = 0.17$, $I^2 = 64.89\%$, $H^2 = 2.85$

Test of $\theta_i = \theta_j$: Q(2) = 5.70, p = 0.06

Test of $\theta = 0$: z = -0.72, p = 0.47

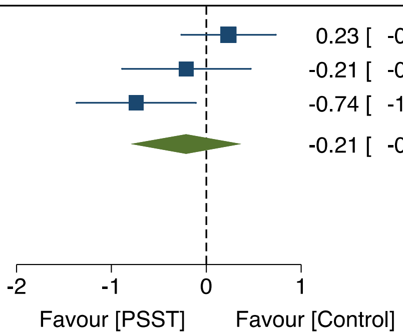


Random-effects DerSimonian-Laird model

## eFigure 1.7. Forest Plot of PSST Association with Parental Quality of Life

| Study | Treatment N | Treatment Mean | Treatment SD | Control N | Control Mean | Control SD | Hedges's g with 95% CI | Weight (%) |
|---|---|---|---|---|---|---|---|---|
| McCann 2013 | 56 | 42.80 | 1.50 | 58 | 42.10 | 1.50 | 0.46 [ 0.09, 0.83] | 64.75 |
| Palermo 2016 | 31 | 46.41 | 10.34 | 30 | 41.57 | 12.67 | 0.41 [ -0.09, 0.91] | 35.25 |
| **Overall** | | | | | | | 0.45 [ 0.15, 0.74] | |

Heterogeneity: $I^2 = 0.00\%$, $H^2 = 0.02$

Test of $\theta_i = \theta_j$: Q(1) = 0.02, p = 0.88

Test of $\theta = 0$: z = 2.94, p = 0.00

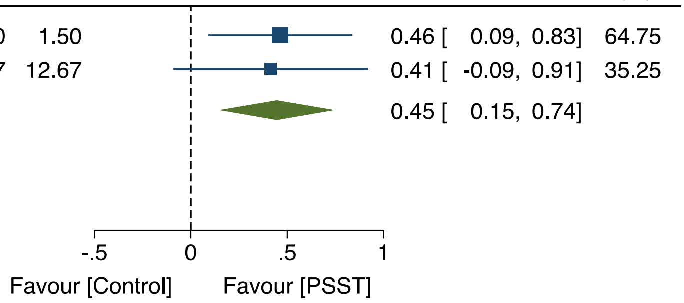


Fixed-effects inverse-variance model

© 2024 American Medical Association. All rights reserved.

## eFigure 1.8. Forest Plot of PSST Association with Pediatric Quality of Life

| Study | Treatment N | Treatment Mean | Treatment SD | Control N | Control Mean | Control SD | Hedges's g with 95% CI | Weight (%) |
|---|---|---|---|---|---|---|---|---|
| Daniel 2015 | 28 | 2.94 | 20.07 | 41 | 1.14 | 22.11 | 0.08 [ -0.39, 0.56] | 12.50 |
| Greenley 2015 | 42 | 87.25 | 10.47 | 24 | 83.94 | 14.85 | 0.27 [ -0.23, 0.77] | 12.43 |
| Modi 2021 | 27 | 84.08 | 12.50 | 29 | 79.84 | 15.20 | 0.30 [ -0.22, 0.82] | 12.37 |
| Nansel 2009 | 58 | 78.60 | 13.10 | 58 | 75.50 | 11.10 | 0.25 [ -0.11, 0.62] | 12.80 |
| Seid 2010 | 60 | 81.40 | 16.30 | 73 | 80.00 | 12.40 | 0.10 [ -0.24, 0.44] | 12.85 |
| Wade 2019 (a) | 22 | 62.00 | 3.35 | 34 | 62.20 | 3.05 | -0.06 [ -0.59, 0.47] | 12.34 |
| Wade 2019 (b) | 43 | 71.70 | 2.64 | 56 | 64.20 | 2.63 | 2.82 [ 2.27, 3.38] | 12.25 |
| Wade 2019 (c) | 51 | 70.90 | 2.82 | 60 | 64.30 | 2.76 | 2.35 [ 1.87, 2.83] | 12.48 |
| **Overall** | | | | | | | 0.76 [ 0.04, 1.47] | |

Heterogeneity: $\tau^2 = 1.00$, $I^2 = 94.90\%$, $H^2 = 19.61$

Test of $\theta_i = \theta_j$: $Q(7) = 137.25$, $p = 0.00$

Test of $\theta = 0$: $z = 2.08$, $p = 0.04$

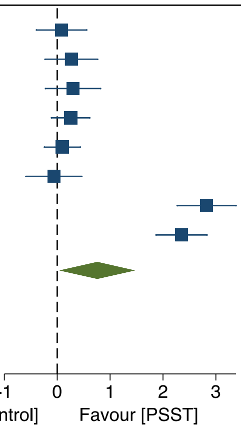


Random-effects DerSimonian-Laird model

## eFigure 1.9. Forest Plot of PSST Association with Pediatric Mental Problems

| Study | Treatment N | Treatment Mean | Treatment SD | Control N | Control Mean | Control SD | Hedges's g with 95% CI | Weight (%) |
|---|---|---|---|---|---|---|---|---|
| Palermo 2016 | 31 | 11.73 | 5.20 | 30 | 12.10 | 5.73 | -0.07 [ -0.56, 0.43] | 11.66 |
| Petranovich 2015 | 57 | 49.37 | 12.13 | 61 | 52.56 | 11.60 | -0.27 [ -0.63, 0.09] | 22.06 |
| Wade 2006a | 16 | 52.50 | 3.99 | 16 | 55.25 | 12.22 | -0.29 [ -0.97, 0.38] | 6.21 |
| Wade 2006b | 20 | 47.39 | 10.30 | 20 | 56.72 | 12.42 | -0.80 [ -1.43, -0.17] | 7.16 |
| Wade 2012 | 16 | 50.72 | 8.09 | 19 | 54.55 | 7.49 | -0.48 [ -1.14, 0.18] | 6.59 |
| Wade 2019 (a) | 22 | 6.10 | 0.70 | 34 | 6.60 | 0.60 | -0.77 [ -1.32, -0.22] | 9.55 |
| Wade 2019 (b) | 43 | 5.20 | 0.60 | 56 | 5.50 | 0.60 | -0.50 [ -0.90, -0.10] | 17.87 |
| Wade 2019 (c) | 51 | 6.00 | 0.60 | 60 | 6.50 | 0.50 | -0.91 [ -1.30, -0.52] | 18.89 |
| **Overall** | | | | | | | -0.51 [ -0.68, -0.34] | |

Heterogeneity: $I^2 = 35.54\%$, $H^2 = 1.55$

Test of $\theta_i = \theta_j$: $Q(7) = 10.86$, $p = 0.14$

Test of $\theta = 0$: $z = -5.88$, $p = 0.00$

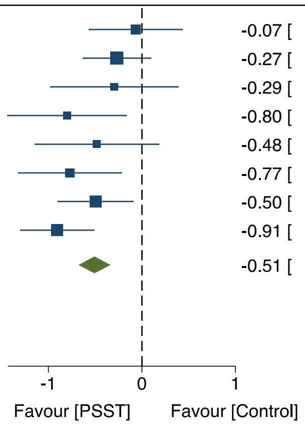


Fixed-effects inverse-variance model

## eFigure 1.10. Forest Plot of PSST Association with Pediatric Social Functioning

| Study | Treatment N | Treatment Mean | Treatment SD | Control N | Control Mean | Control SD | Hedges's g with 95% CI | Weight (%) |
|---|---|---|---|---|---|---|---|---|
| DaWalt 2018 | 16 | 0.33 | 0.22 | 25 | -0.21 | 0.17 | 2.78 [ 1.92, 3.64] | 31.91 |
| Palermo 2016 | 31 | 14.81 | 6.34 | 30 | 15.07 | 6.62 | -0.04 [ -0.54, 0.46] | 34.44 |
| Wade 2006b | 20 | 53.15 | 9.89 | 20 | 45.50 | 11.50 | 0.70 [ 0.07, 1.33] | 33.65 |
| **Overall** | | | | | | | 1.11 [ -0.36, 2.57] | |

Heterogeneity: $\tau^2 = 1.55$, $I^2 = 93.53\%$, $H^2 = 15.46$

Test of $\theta_i = \theta_j$: $Q(2) = 30.91$, $p = 0.00$

Test of $\theta = 0$: $z = 1.48$, $p = 0.14$

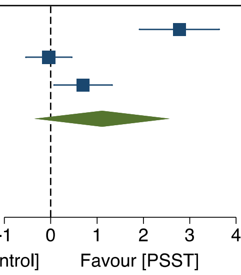


Random-effects DerSimonian-Laird model

© 2024 American Medical Association. All rights reserved.

## eFigure 1.11. Forest Plot of PSST Association with Parent-child conflict

| Study | Treatment N | Treatment Mean | Treatment SD | Control N | Control Mean | Control SD | Hedges's g with 95% CI | Weight (%) |
|---|---|---|---|---|---|---|---|---|
| DaWalt 2018 | 16 | -1.50 | 2.00 | 25 | -0.48 | 1.59 | -0.57 [ -1.20, 0.06] | 12.58 |
| Nansel 2009 | 58 | 25.00 | 8.30 | 58 | 25.60 | 8.80 | -0.07 [ -0.43, 0.29] | 37.87 |
| Petranovich 2015 | 61 | 1.95 | 0.70 | 64 | 2.37 | 0.88 | -0.52 [ -0.88, -0.17] | 39.40 |
| Wade 2006a | 16 | -3.38 | 5.47 | 16 | -0.37 | 1.59 | -0.73 [ -1.43, -0.03] | 10.15 |
| **Overall** | | | | | | | -0.38 [ -0.60, -0.16] | |

Heterogeneity: $I^2 = 36.98\%$, $H^2 = 1.59$

Test of $\theta_i = \theta_j$: Q(3) = 4.76, p = 0.19

Test of $\theta = 0$: z = -3.33, p = 0.00

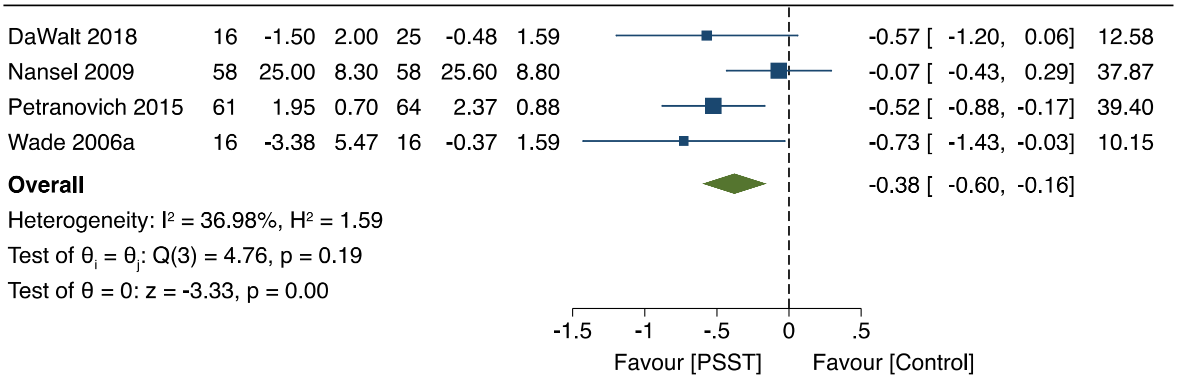


Fixed-effects inverse-variance model

© 2024 American Medical Association. All rights reserved.

**eFigure 2. Subgroup Analyses of Each Outcome According to Children- and Intervention-Level Factors**

**eFigure 2.1 Subgroup Analysis of PSST on Parental Depression Changes (Overall)**

| Subgroups | PSST | Control | Std. Mean Difference (95% *CI*) | $I^2$ (%) |
|---|---|---|---|---|
| **Overall** | 1254 | 1517 | -0.45 (-0.66, -0.23) | 85.29 |
| **Child age** | | | | |
| ≤ 10 years | 991 | 1211 | -0.39 (-0.52, -0.26) | 92.01 |
| > 10 years | 263 | 306 | -0.55 (-1.17, 0.07) | 50.10 |
| **Pediatric illness duration** | | | | |
| Newly diagnosed | 1052 | 1275 | -0.40 (-0.52, -0.28) | 46.35 |
| Not newly diagnosed | 202 | 242 | -0.52 (-1.18, 0.14) | 92.13 |
| **Intervention approach** | | | | |
| Face to face | 769 | 914 | -0.38 (-0.62, -0.14) | 79.69 |
| Online | 485 | 603 | -0.55 (-1.02, -0.09) | 91.20 |
| **Mode of delivery** | | | | |
| Individual intervention | 990 | 1209 | -0.39 (-0.52, -0.27) | 48.69 |
| Parent-child intervention | 213 | 250 | -0.38 (-1.09, 0.34) | 92.54 |
| Group intervention | 51 | 58 | -1.08 (-2.94, 0.78) | 94.58 |
| **Duration of intervention** | | | | |
| 5–8 weeks | 1009 | 1235 | -0.48 (-0.67, -0.29) | 76.31 |
| > 8 weeks | 245 | 282 | -0.34 (-0.95, 0.26) | 91.14 |
| **Number of sessions** | | | | |
| < 8 sessions | 116 | 120 | -0.33 (-0.59, -0.08) | 0.00 |
| 8–12 sessions | 1002 | 1227 | -0.48 (-0.67, -0.28) | 75.62 |
| > 12 sessions | 136 | 170 | -0.39 (-1.54, 0.76) | 95.52 |

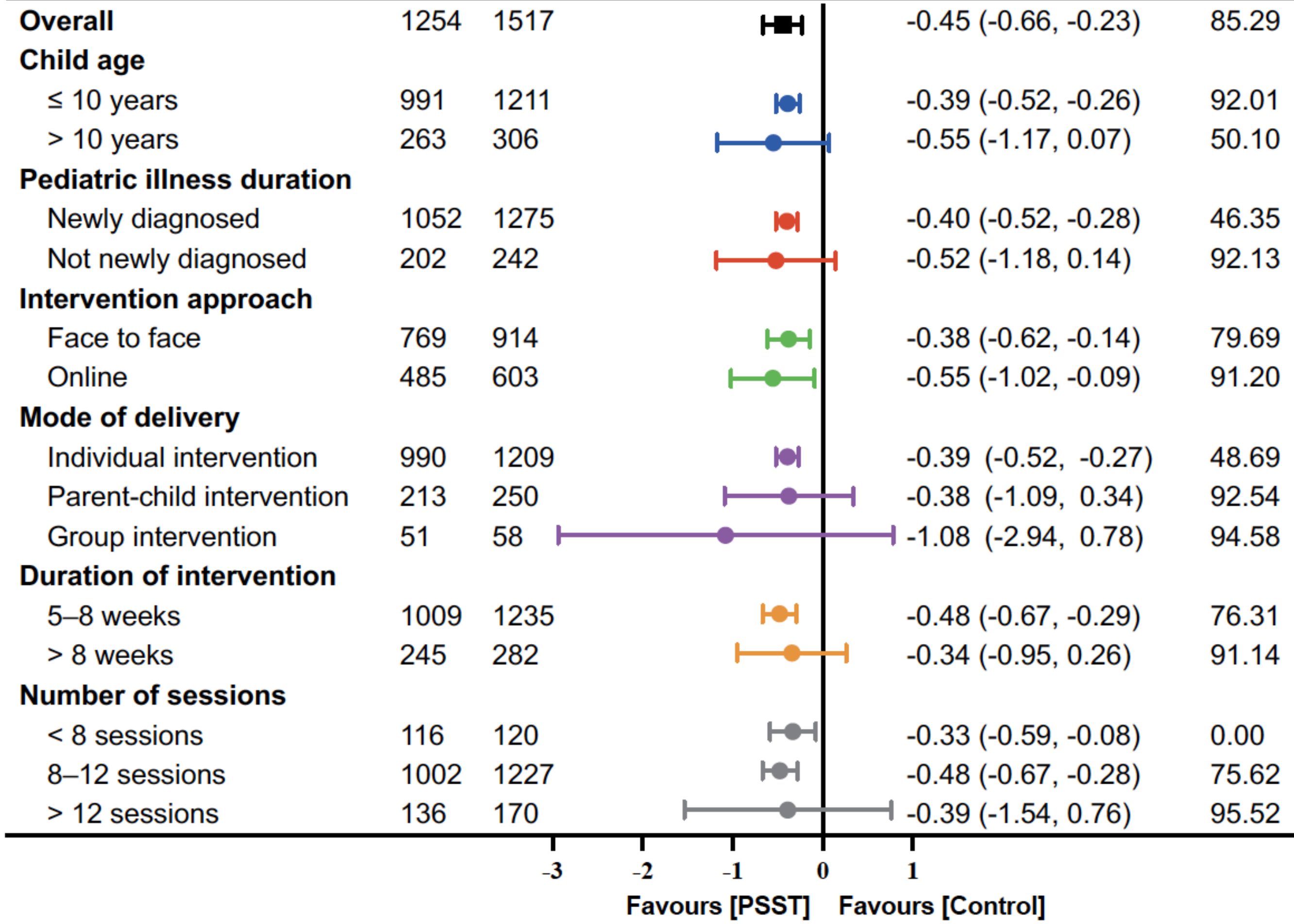


© 2024 American Medical Association. All rights reserved.

## eFigure 2.2 Subgroup Analysis of Childhood Medical Conditions on Parental Depression Changes

| Study | Treatment N | Treatment Mean | Treatment SD | Control N | Control Mean | Control SD | Hedges's g with 95% CI | Weight (%) |
|---|---|---|---|---|---|---|---|---|
| **Autism spectrum disorder (ASD)** | | | | | | | | |
| DaWalt 2018 | 19 | -5.44 | 3.01 | 26 | .11 | 2.38 | -2.05 [ -2.77, -1.33] | 4.27 |
| Feinberg 2014 | 53 | 4.6 | 3.5 | 58 | 6.9 | 5 | -0.53 [ -0.90, -0.15] | 6.51 |
| Heterogeneity: $\tau^2$ = 1.07, $I^2$ = 92.61%, $H^2$ = 13.54 | | | | | | | -1.25 [ -2.75, 0.24] | |
| Test of $\theta_i = \theta_j$: Q(1) = 13.54, p = 0.00 | | | | | | | | |
| **Cancer** | | | | | | | | |
| Askins 2009 (a) | 66 | 3.34 | 1.41 | 93 | 3.94 | .68 | -0.57 [ -0.89, -0.25] | 6.88 |
| Askins 2009 (b) | 65 | 3.24 | 1.4 | 104 | 3.94 | .71 | -0.68 [ -0.99, -0.36] | 6.91 |
| Phipps 2020 (a) | 244 | 5.15 | 7.37 | 310 | 7.51 | 5.55 | -0.37 [ -0.54, -0.20] | 7.73 |
| Phipps 2020 (b) | 244 | 5.15 | 7.37 | 311 | 8.14 | 5.67 | -0.46 [ -0.63, -0.29] | 7.73 |
| Sahler 2005 | 191 | -.73 | 1.66 | 195 | -.17 | 1.66 | -0.34 [ -0.54, -0.14] | 7.58 |
| Sahler 2013 | 96 | 3.25 | 1.27 | 108 | 3.27 | 1.35 | -0.02 [ -0.29, 0.26] | 7.18 |
| Heterogeneity: $\tau^2$ = 0.02, $I^2$ = 60.24%, $H^2$ = 2.51 | | | | | | | -0.39 [ -0.54, -0.25] | |
| Test of $\theta_i = \theta_j$: Q(5) = 12.57, p = 0.03 | | | | | | | | |
| **Chronic pain** | | | | | | | | |
| Palermo 2016 | 31 | 7.87 | 5.82 | 30 | 9.33 | 8.51 | -0.20 [ -0.70, 0.30] | 5.67 |
| Heterogeneity: $\tau^2$ = 0.00, $I^2$ = .%, $H^2$ = . | | | | | | | -0.20 [ -0.70, 0.30] | |
| Test of $\theta_i = \theta_j$: Q(0) = 0.00, p = . | | | | | | | | |
| **Sickle cell disease (SCD)** | | | | | | | | |
| Asnani 2021 | 32 | 14 | 10.3 | 32 | 15.7 | 12.3 | -0.15 [ -0.63, 0.34] | 5.76 |
| Heterogeneity: $\tau^2$ = 0.00, $I^2$ = .%, $H^2$ = . | | | | | | | -0.15 [ -0.63, 0.34] | |
| Test of $\theta_i = \theta_j$: Q(0) = -0.00, p = . | | | | | | | | |
| **Traumatic brain injury (TBI)** | | | | | | | | |
| Petranovich 2015 | 61 | 11.1 | 9.3 | 64 | 15.4 | 11.7 | -0.40 [ -0.76, -0.05] | 6.67 |
| Wade 2006a | 16 | 52.62 | 10.24 | 16 | 56.19 | 12.59 | -0.30 [ -0.98, 0.38] | 4.50 |
| Wade 2006b | 20 | 9.25 | 7.09 | 20 | 18.15 | 13.49 | -0.81 [ -1.44, -0.18] | 4.78 |
| Wade 2019 (a) | 22 | 15.1 | 1.81 | 34 | 14.2 | 1.73 | 0.50 [ -0.03, 1.04] | 5.40 |
| Wade 2019 (b) | 43 | 13.5 | 1.42 | 56 | 12.8 | 1.49 | 0.48 [ 0.08, 0.88] | 6.35 |
| Wade 2019 (c) | 51 | 12.8 | 1.53 | 60 | 15.5 | 1.56 | -1.73 [ -2.17, -1.30] | 6.10 |
| Heterogeneity: $\tau^2$ = 0.73, $I^2$ = 92.54%, $H^2$ = 13.40 | | | | | | | -0.38 [ -1.09, 0.34] | |
| Test of $\theta_i = \theta_j$: Q(5) = 67.00, p = 0.00 | | | | | | | | |
| **Overall** | | | | | | | -0.45 [ -0.66, -0.23] | |
| Heterogeneity: $\tau^2$ = 0.15, $I^2$ = 85.29%, $H^2$ = 6.80 | | | | | | | | |
| Test of $\theta_i = \theta_j$: Q(15) = 101.99, p = 0.00 | | | | | | | | |
| Test of group differences: $Q_b(4)$ = 2.71, p = 0.61 | | | | | | | | |

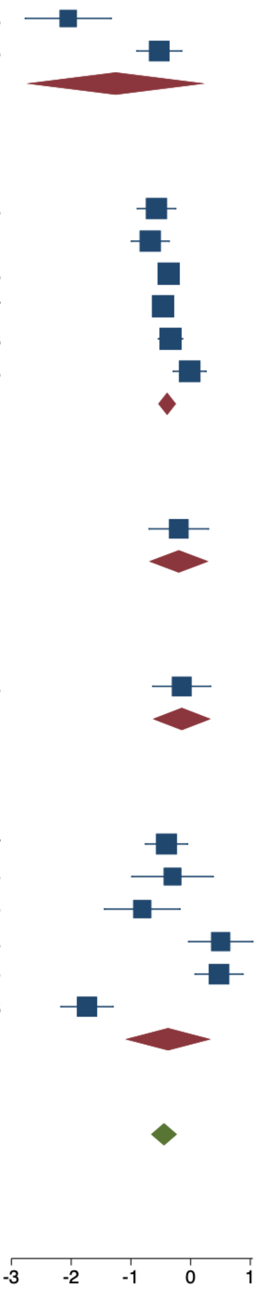


Random-effects DerSimonian-Laird model

© 2024 American Medical Association. All rights reserved.

**eFigure 2.3 Subgroup Analysis of PSST on Parental Distress Changes (Overall)**

| Subgroups | PSST | Control | Std. Mean Difference (95% *CI*) | $I^2$ (%) |
|---|---|---|---|---|
| **Overall** | 1255 | 1518 | -0.61 (-0.81, -0.40) | 83.88 |
| **Child age** | | | | |
| ≤ 10 years | 939 | 1161 | -0.42 (-0.57, -0.28) | 58.90 |
| > 10 years | 316 | 357 | -0.75 (-1.19, -0.32) | 85.81 |
| **Pediatric illness duration** | | | | |
| Newly diagnosed | 1000 | 1225 | -0.43 (-0.56, -0.30) | 52.84 |
| Not newly diagnosed | 255 | 293 | -0.78 (-1.28, -0.29) | 86.48 |
| **Intervention approach** | | | | |
| Face to face | 698 | 838 | -0.35 (-0.51, -0.20) | 44.54 |
| Online | 501 | 622 | -0.80 (-1.19, -0.41) | 87.29 |
| Face to face + online | 56 | 58 | -1.57 (-1.99, -1.15) | NA |
| **Mode of delivery** | | | | |
| Individual intervention | 1026 | 1249 | -0.50 (-0.72, -0.28) | 83.18 |
| Parent-child intervention | 229 | 269 | -0.75 (-1.19, -0.31) | 81.01 |
| **Duration of intervention** | | | | |
| 5–8 weeks | 1026 | 1249 | -0.50 (-0.72, -0.28) | 83.18 |
| > 8 weeks | 229 | 269 | -0.75 (-1.19, -0.31) | 81.01 |
| **Number of sessions** | | | | |
| < 8 sessions | 87 | 88 | -0.76 (-2.37, 0.85) | 95.94 |
| 8–12 sessions | 1016 | 1241 | -0.42 (-0.55, -0.29) | 48.05 |
| > 12 sessions | 152 | 189 | -0.92 (-1.45, -0.38) | 81.06 |

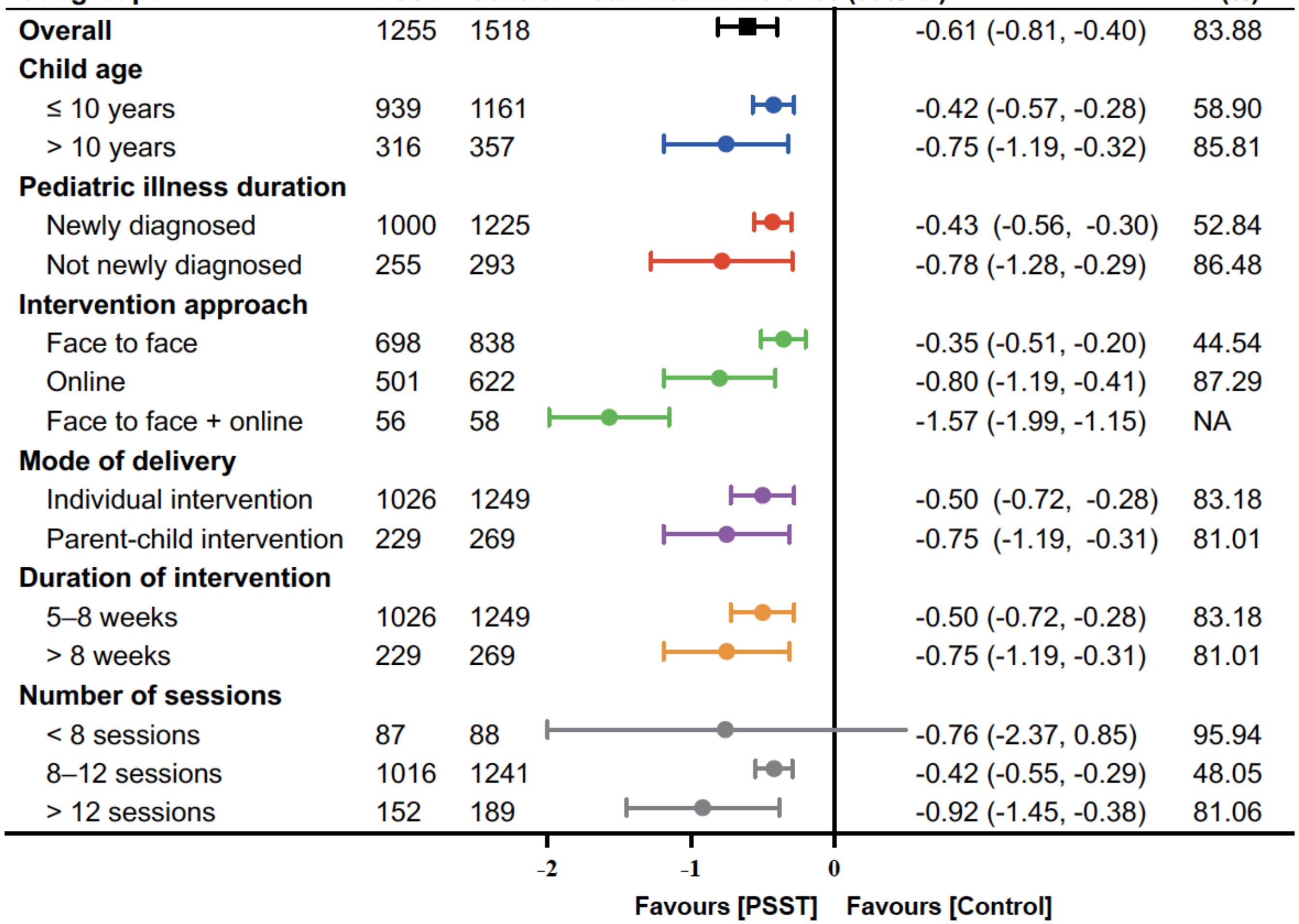


© 2024 American Medical Association. All rights reserved.

## eFigure 2.4 Subgroup Analysis of Childhood Medical Conditions on Parental Distress Changes

| Study | Treatment N | Treatment Mean | Treatment SD | Control N | Control Mean | Control SD | Hedges's g with 95% CI | Weight (%) |
|---|---|---|---|---|---|---|---|---|
| **Cancer** | | | | | | | | |
| Askins 2009 (a) | 66 | 33.7 | 35.98 | 93 | 55.7 | 22.76 | -0.76 [ -1.08, -0.43] | 6.88 |
| Askins 2009 (b) | 65 | 34.6 | 35.08 | 104 | 55.7 | 24.07 | -0.73 [ -1.05, -0.41] | 6.93 |
| Phipps 2020 (a) | 244 | 86.17 | 42.38 | 310 | 99.17 | 42.18 | -0.31 [ -0.48, -0.14] | 7.85 |
| Phipps 2020 (b) | 244 | 84.44 | 41.79 | 311 | 104.24 | 42.86 | -0.47 [ -0.64, -0.30] | 7.84 |
| Sahler 2002 | 33 | 80.76 | 38.81 | 40 | 98.1 | 48.5 | -0.39 [ -0.85, 0.07] | 5.88 |
| Sahler 2005 | 191 | -19.76 | 38.75 | 195 | -6.68 | 38.75 | -0.34 [ -0.54, -0.14] | 7.68 |
| Sahler 2013 | 96 | 33.6 | 35.86 | 108 | 38.3 | 36.37 | -0.13 [ -0.40, 0.14] | 7.23 |
| Heterogeneity: $\tau^2 = 0.02$, $I^2 = 58.90\%$, $H^2 = 2.43$ | | | | | | | -0.42 [ -0.57, -0.28] | |
| Test of $\theta_i = \theta_j$: Q(6) = 14.60, p = 0.02 | | | | | | | | |
| **Chronic pain** | | | | | | | | |
| Palermo 2016 | 31 | 29.35 | 33.6 | 30 | 26.87 | 36.87 | 0.07 [ -0.43, 0.57] | 5.62 |
| Heterogeneity: $\tau^2 = 0.00$, $I^2 = .\%$, $H^2 = .$ | | | | | | | 0.07 [ -0.43, 0.57] | |
| Test of $\theta_i = \theta_j$: Q(0) = 0.00, p = . | | | | | | | | |
| **First-episode psychosis (FEP)** | | | | | | | | |
| McCann 2013 | 56 | 19.9 | 1.2 | 58 | 21.8 | 1.2 | -1.57 [ -1.99, -1.15] | 6.20 |
| Heterogeneity: $\tau^2 = 0.00$, $I^2 = .\%$, $H^2 = .$ | | | | | | | -1.57 [ -1.99, -1.15] | |
| Test of $\theta_i = \theta_j$: Q(0) = 0.00, p = . | | | | | | | | |
| **Traumatic brain injury (TBI)** | | | | | | | | |
| Petranovich 2015 | 61 | 47.8 | 12.5 | 64 | 53.9 | 12 | -0.50 [ -0.85, -0.14] | 6.67 |
| Wade 2006a | 16 | 53.13 | 12.03 | 16 | 55.13 | 14.09 | -0.15 [ -0.83, 0.53] | 4.41 |
| Wade 2006b | 20 | 52.33 | 10.69 | 20 | 58.37 | 11.49 | -0.53 [ -1.15, 0.09] | 4.77 |
| Wade 2012 | 16 | 48.85 | 7.92 | 19 | 54.03 | 9.42 | -0.58 [ -1.24, 0.09] | 4.48 |
| Wade 2019 (a) | 22 | 56.1 | 2.46 | 34 | 56.9 | 1.93 | -0.37 [ -0.90, 0.17] | 5.35 |
| Wade 2019 (b) | 43 | 54.7 | 1.88 | 56 | 56.9 | 1.69 | -1.23 [ -1.66, -0.80] | 6.11 |
| Wade 2019 (c) | 51 | 53.3 | 2.02 | 60 | 56.6 | 1.77 | -1.74 [ -2.17, -1.30] | 6.07 |
| Heterogeneity: $\tau^2 = 0.28$, $I^2 = 81.01\%$, $H^2 = 5.27$ | | | | | | | -0.75 [ -1.19, -0.31] | |
| Test of $\theta_i = \theta_j$: Q(6) = 31.60, p = 0.00 | | | | | | | | |
| **Overall** | | | | | | | -0.61 [ -0.81, -0.40] | |
| Heterogeneity: $\tau^2 = 0.14$, $I^2 = 83.88\%$, $H^2 = 6.20$ | | | | | | | | |
| Test of $\theta_i = \theta_j$: Q(15) = 93.05, p = 0.00 | | | | | | | | |
| Test of group differences: $Q_b(3) = 32.51$, p = 0.00 | | | | | | | | |

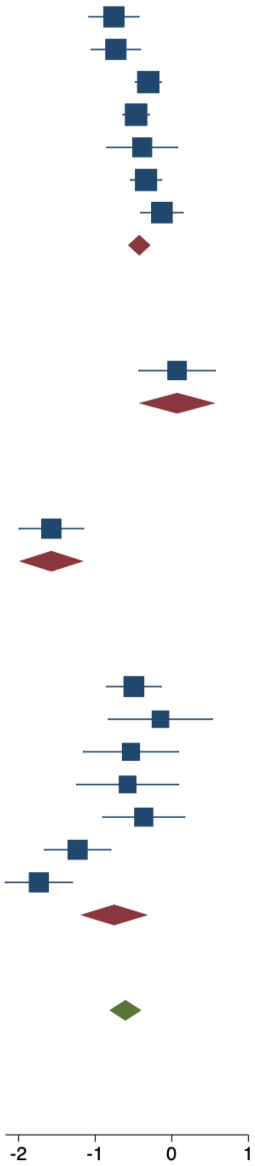


Random-effects DerSimonian-Laird model

© 2024 American Medical Association. All rights reserved.

**eFigure 2.5 Subgroup Analysis of PSST on Parental Problem-solving Skills Changes (Overall)**

| Subgroups | PSST | Control | Std. Mean Difference (95% *CI*) | $I^2$ (%) |
|---|---|---|---|---|
| **Overall** | 1128 | 1378 | 0.43 (0.27, 0.58) | 64.28 |
| **Child age** | | | | |
| ≤ 10 years | 1062 | 1303 | 0.36 (0.24, 0.48) | 38.90 |
| > 10 years | 67 | 75 | 0.95 (-0.01, 1.91) | 86.14 |
| **Pediatric illness duration** | | | | |
| Newly diagnosed | 1033 | 1262 | 0.37 (0.24, 0.50) | 49.85 |
| Not newly diagnosed | 95 | 116 | 0.65 (0.12, 1.19) | 78.47 |
| **Intervention approach** | | | | |
| Face to face | 761 | 902 | 0.43 (0.22, 0.63) | 72.11 |
| Online | 330 | 423 | 0.46 (0.09, 0.83) | 72.92 |
| Face to face + online | 37 | 53 | 0.44 (0.02, 0.85) | 0.00 |
| **Mode of delivery** | | | | |
| Individual intervention | 1023 | 1248 | 0.37 (0.24, 0.50) | 49.21 |
| Parent-child intervention | 29 | 32 | 0.49 (-0.01, 0.98) | 0.00 |
| Group intervention | 76 | 98 | 0.84 (-0.13, 1.82) | 89.02 |
| **Duration of intervention** | | | | |
| 5–8 weeks | 1048 | 1286 | 0.45 (0.27, 0.63) | 72.10 |
| > 8 weeks | 80 | 92 | 0.32 (0.02, 0.62) | 0.00 |
| **Number of sessions** | | | | |
| < 8 sessions | 153 | 172 | 0.26 (0.04, 0.48) | 0.00 |
| 8–12 sessions | 955 | 1186 | 0.50 (0.29, 0.70) | 78.61 |
| > 12 sessions | 20 | 20 | 0.43 (-0.19, 1.04) | NA |

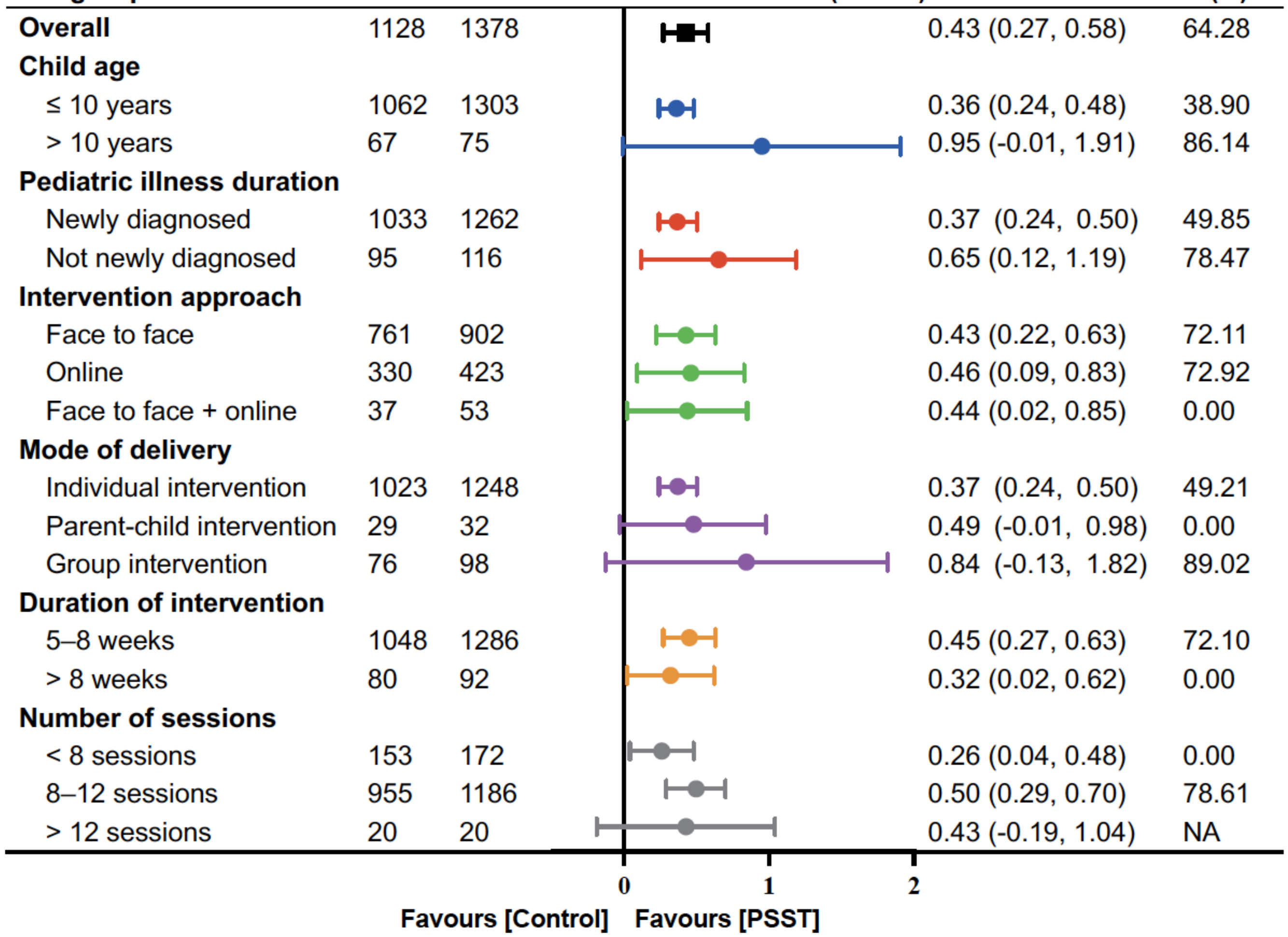


© 2024 American Medical Association. All rights reserved.

## eFigure 2.6 Subgroup Analysis of Childhood Medical Conditions on Parental Problem-solving Skills Changes

| Study | Treatment N | Treatment Mean | Treatment SD | Control N | Control Mean | Control SD | Hedges's g with 95% CI | Weight (%) |
|---|---|---|---|---|---|---|---|---|
| **Autism spectrum disorder (ASD)** | | | | | | | | |
| DaWalt 2018 | 16 | .44 | .22 | 25 | .02 | .18 | 2.10 [ 1.33, 2.86] | 3.05 |
| Feinberg 2014 | 53 | 17.89 | 3.51 | 57 | 17.65 | 4.17 | 0.06 [ -0.31, 0.43] | 7.43 |
| Heterogeneity: $\tau^2 = 1.98$, $I^2 = 95.46\%$, $H^2 = 22.03$ | | | | | | | 1.05 [ -0.94, 3.05] | |
| Test of $\theta_i = \theta_j$: Q(1) = 22.03, p = 0.00 | | | | | | | | |
| **Cancer** | | | | | | | | |
| Askins 2009 (a) | 66 | 14.6 | 2.06 | 93 | 13.3 | 1.45 | 0.75 [ 0.42, 1.07] | 8.31 |
| Askins 2009 (b) | 65 | 14.5 | 2.01 | 104 | 13.3 | 1.53 | 0.69 [ 0.37, 1.01] | 8.45 |
| Phipps 2020 (a) | 244 | 14.43 | 2.8 | 310 | 13.77 | 2.63 | 0.24 [ 0.08, 0.41] | 11.49 |
| Phipps 2020 (b) | 244 | 14.68 | 2.82 | 311 | 13.59 | 2.64 | 0.40 [ 0.23, 0.57] | 11.48 |
| Sahler 2002 | 33 | 74.58 | 14.48 | 40 | 70.68 | 13.49 | 0.28 [ -0.18, 0.73] | 6.03 |
| Sahler 2005 | 191 | .46 | 1.84 | 195 | .01 | 1.84 | 0.24 [ 0.04, 0.44] | 10.86 |
| Sahler 2013 | 96 | 14.4 | 2.16 | 108 | 13.7 | 2.18 | 0.32 [ 0.05, 0.60] | 9.29 |
| Heterogeneity: $\tau^2 = 0.02$, $I^2 = 54.62\%$, $H^2 = 2.20$ | | | | | | | 0.39 [ 0.26, 0.53] | |
| Test of $\theta_i = \theta_j$: Q(6) = 13.22, p = 0.04 | | | | | | | | |
| **Chronic pain** | | | | | | | | |
| Palermo 2016 | 31 | 14.73 | 2 | 30 | 13.75 | 2.39 | 0.44 [ -0.06, 0.94] | 5.44 |
| Heterogeneity: $\tau^2 = 0.00$, $I^2 = .\%$, $H^2 = .$ | | | | | | | 0.44 [ -0.06, 0.94] | |
| Test of $\theta_i = \theta_j$: Q(0) = 0.00, p = . | | | | | | | | |
| **Epilepsy** | | | | | | | | |
| Modi 2016 | 9 | 114.4 | 12.8 | 12 | 107.1 | 10.9 | 0.60 [ -0.25, 1.45] | 2.58 |
| Heterogeneity: $\tau^2 = 0.00$, $I^2 = .\%$, $H^2 = .$ | | | | | | | 0.60 [ -0.25, 1.45] | |
| Test of $\theta_i = \theta_j$: Q(0) = 0.00, p = . | | | | | | | | |
| **Sickle cell disease (SCD)** | | | | | | | | |
| Asnani 2021 | 32 | 93.6 | 10.6 | 32 | 91.6 | 9.8 | 0.19 [ -0.29, 0.68] | 5.66 |
| Daniel 2015 | 28 | 111.81 | 11.48 | 41 | 106.71 | 14.17 | 0.38 [ -0.10, 0.86] | 5.74 |
| Heterogeneity: $\tau^2 = 0.00$, $I^2 = 0.00\%$, $H^2 = 1.00$ | | | | | | | 0.29 [ -0.05, 0.63] | |
| Test of $\theta_i = \theta_j$: Q(1) = 0.30, p = 0.59 | | | | | | | | |
| **Traumatic brain injury (TBI)** | | | | | | | | |
| Wade 2006b | 20 | 73.45 | 9.61 | 20 | 69.16 | 10.02 | 0.43 [ -0.19, 1.04] | 4.19 |
| Heterogeneity: $\tau^2 = 0.00$, $I^2 = .\%$, $H^2 = .$ | | | | | | | 0.43 [ -0.19, 1.04] | |
| Test of $\theta_i = \theta_j$: Q(0) = 0.00, p = . | | | | | | | | |
| **Overall** | | | | | | | 0.43 [ 0.27, 0.58] | |
| Heterogeneity: $\tau^2 = 0.04$, $I^2 = 64.28\%$, $H^2 = 2.80$ | | | | | | | | |
| Test of $\theta_i = \theta_j$: Q(13) = 36.39, p = 0.00 | | | | | | | | |
| Test of group differences: $Q_b(5) = 1.04$, p = 0.96 | | | | | | | | |

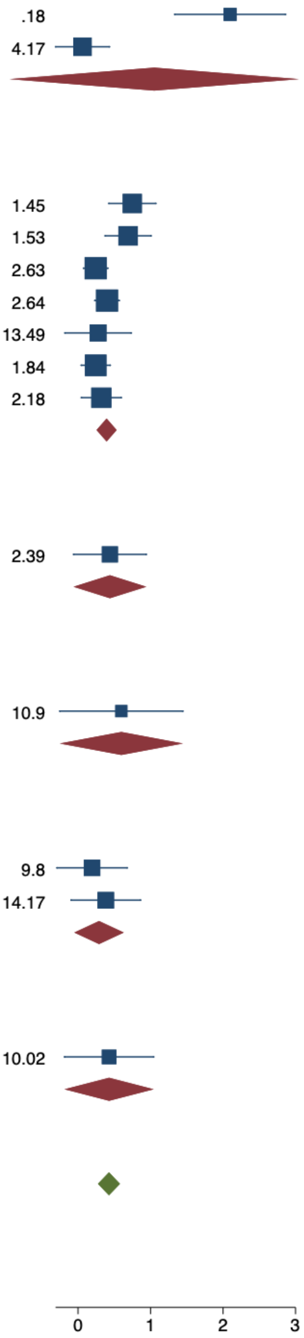


Random-effects DerSimonian-Laird model

© 2024 American Medical Association. All rights reserved.

**eFigure 2.7 Subgroup Analysis of PSST on Parental Post-traumatic Stress Changes (Overall)**

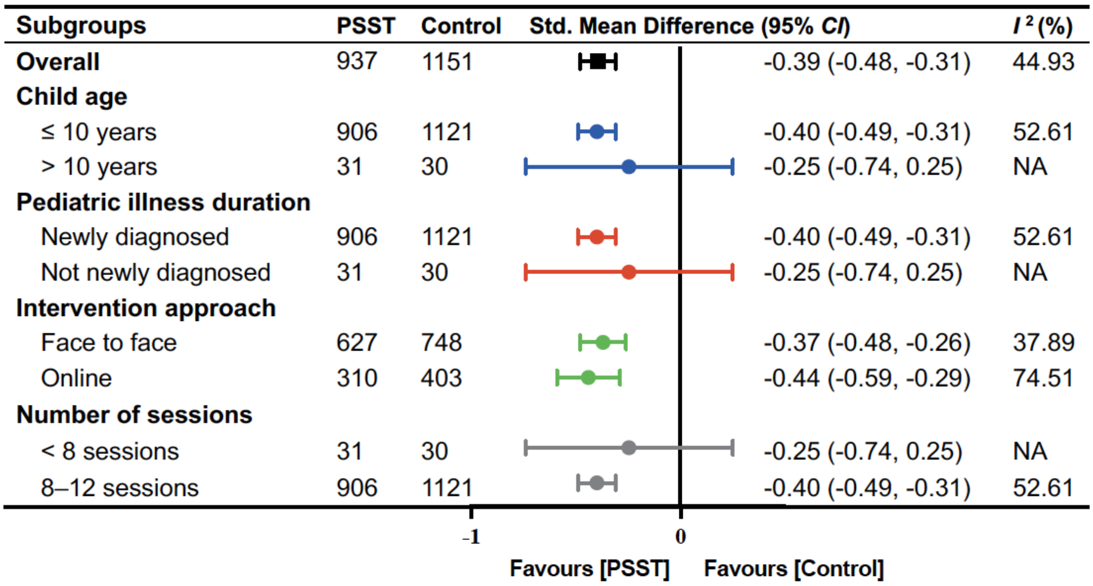


**eFigure 2.8 Subgroup Analysis of Childhood Medical Conditions on Parental Post-traumatic Stress Changes**

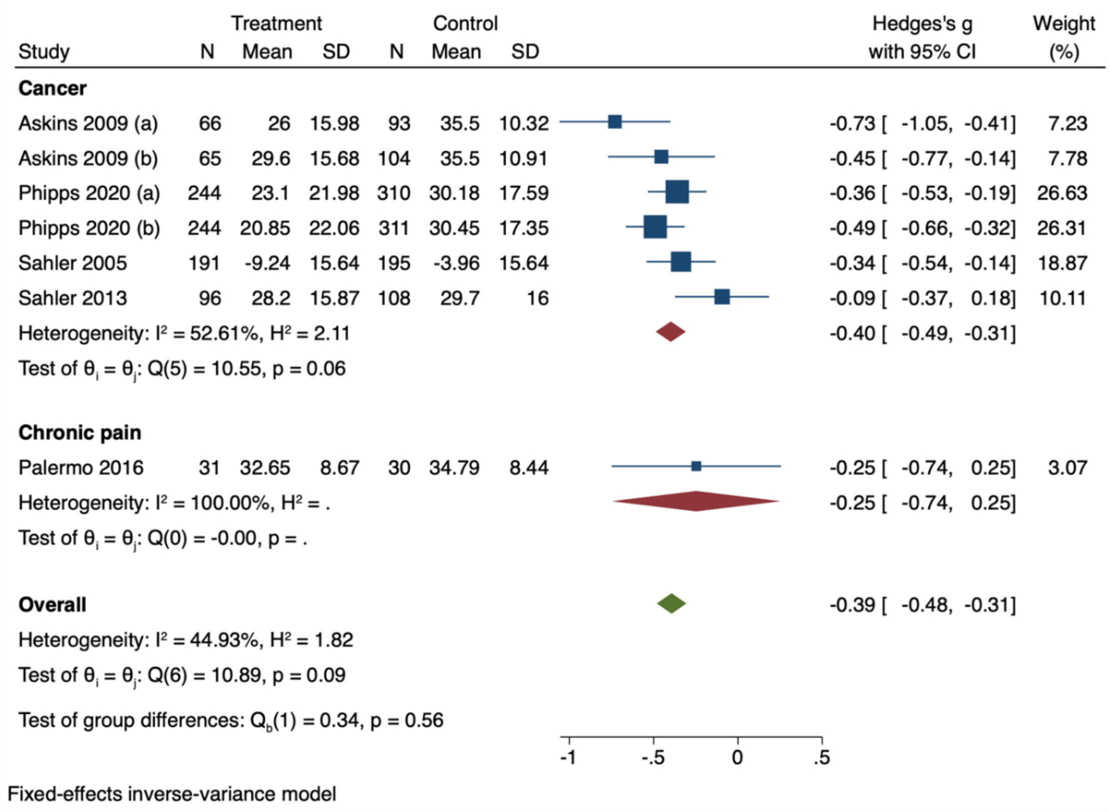


© 2024 American Medical Association. All rights reserved.

**eFigure 2.9 Subgroup Analysis of PSST on Parenting Stress Changes (Overall)**

| Subgroups | PSST | Control | Std. Mean Difference (95% *CI*) | $I^2$ (%) |
|---|---|---|---|---|
| **Overall** | 188 | 203 | -0.62 (-1.05, -0.19) | 76.24 |
| **Child age** | | | | |
| ≤ 10 years | 85 | 90 | -0.43 (-0.72, -0.13) | 0.00 |
| > 10 years | 103 | 113 | -0.73 (-1.47, 0.00) | 84.49 |
| **Pediatric illness duration** | | | | |
| Newly diagnosed | 85 | 90 | -0.38 (-0.75, 0.00) | NA |
| Not newly diagnosed | 103 | 113 | -0.68 (-1.22, -0.14) | 78.71 |
| **Intervention approach** | | | | |
| Face to face | 132 | 145 | -0.40 (-0.64, -0.17) | 0.00 |
| Face to face + online | 56 | 58 | -1.34 (-1.74, -0.93) | NA |
| **Mode of delivery** | | | | |
| Individual intervention | 140 | 146 | -0.64 (-1.34, 0.06) | 87.89 |
| Group intervention | 48 | 57 | -0.56 (-0.95, -0.17) | 0.00 |
| **Duration of intervention** | | | | |
| 5–8 weeks | 156 | 171 | -0.64 (-1.18, -0.10) | 81.86 |
| > 8 weeks | 32 | 32 | -0.51 (-1.01, -0.02) | NA |
| **Number of sessions** | | | | |
| < 8 sessions | 172 | 178 | -0.61 (-1.13, -0.09) | 82.18 |
| 8–12 sessions | 16 | 25 | -0.63 (-1.26, -0.00) | NA |

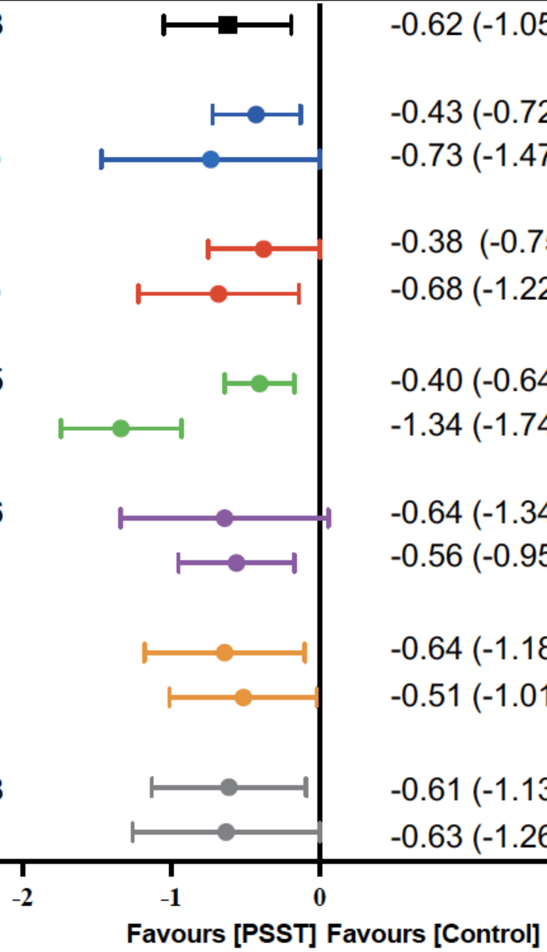


© 2024 American Medical Association. All rights reserved.

## eFigure 2.10 Subgroup Analysis of Childhood Medical Conditions on Parenting Stress Changes

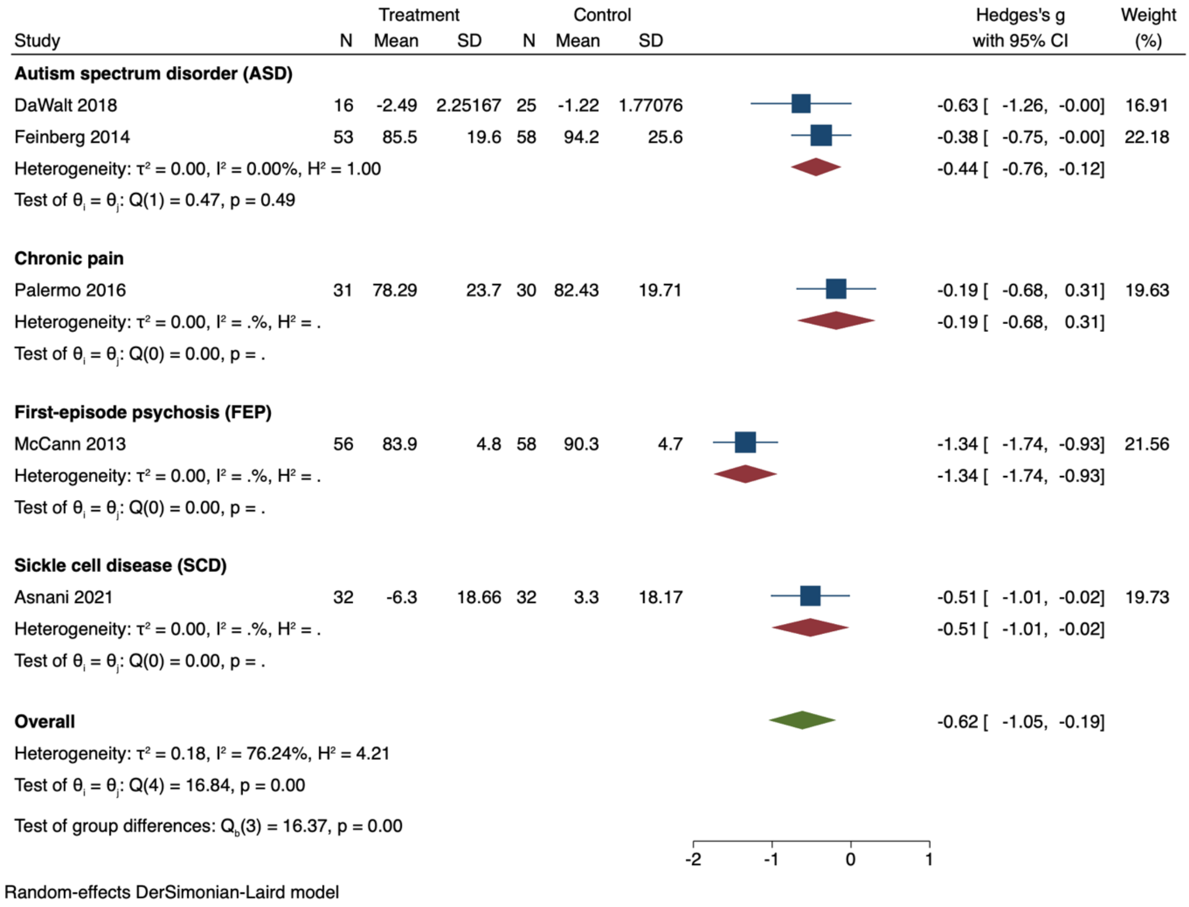


© 2024 American Medical Association. All rights reserved.

**eFigure 2.11 Subgroup Analysis of PSST on Pediatric Quality of Life Changes (Overall)**

| Subgroups | PSST | Control | Std. Mean Difference (95% *CI*) | $I^2$ (%) |
|---|---|---|---|---|
| **Overall** | 331 | 375 | 0.76 (0.04, 1.47) | 94.9 |
| **Child age** | | | | |
| ≤ 10 years | 115 | 143 | 0.14 (-0.11, 0.38) | 0.00 |
| > 10 years | 216 | 232 | 1.12 (0.00, 2.25) | 96.41 |
| **Pediatric illness duration** | | | | |
| Newly diagnosed | 27 | 29 | 0.30 (-0.22, 0.82) | NA |
| Not newly diagnosed | 304 | 346 | 0.82 (0.02, 1.63) | 95.58 |
| **Intervention approach** | | | | |
| Face to face | 82 | 107 | 0.05 (-0.24, 0.34) | 0.00 |
| Online | 136 | 140 | 1.81 (0.27, 3.35) | 96.32 |
| Face to face + online | 113 | 128 | 0.22 (-0.04, 0.47) | 0.00 |
| **Mode of delivery** | | | | |
| Parent-child intervention | 303 | 334 | 0.85 (0.05, 1.66) | 95.45 |
| Group intervention | 28 | 41 | 0.08 (-0.39, 0.56) | NA |
| **Number of sessions** | | | | |
| < 8 sessions | 130 | 138 | 0.13 (-0.11, 0.38) | 0.00 |
| 8–12 sessions | 85 | 87 | 0.27 (-0.03, 0.57) | 0.00 |
| > 12 sessions | 116 | 150 | 1.70 (-0.02,3.43) | 96.94 |

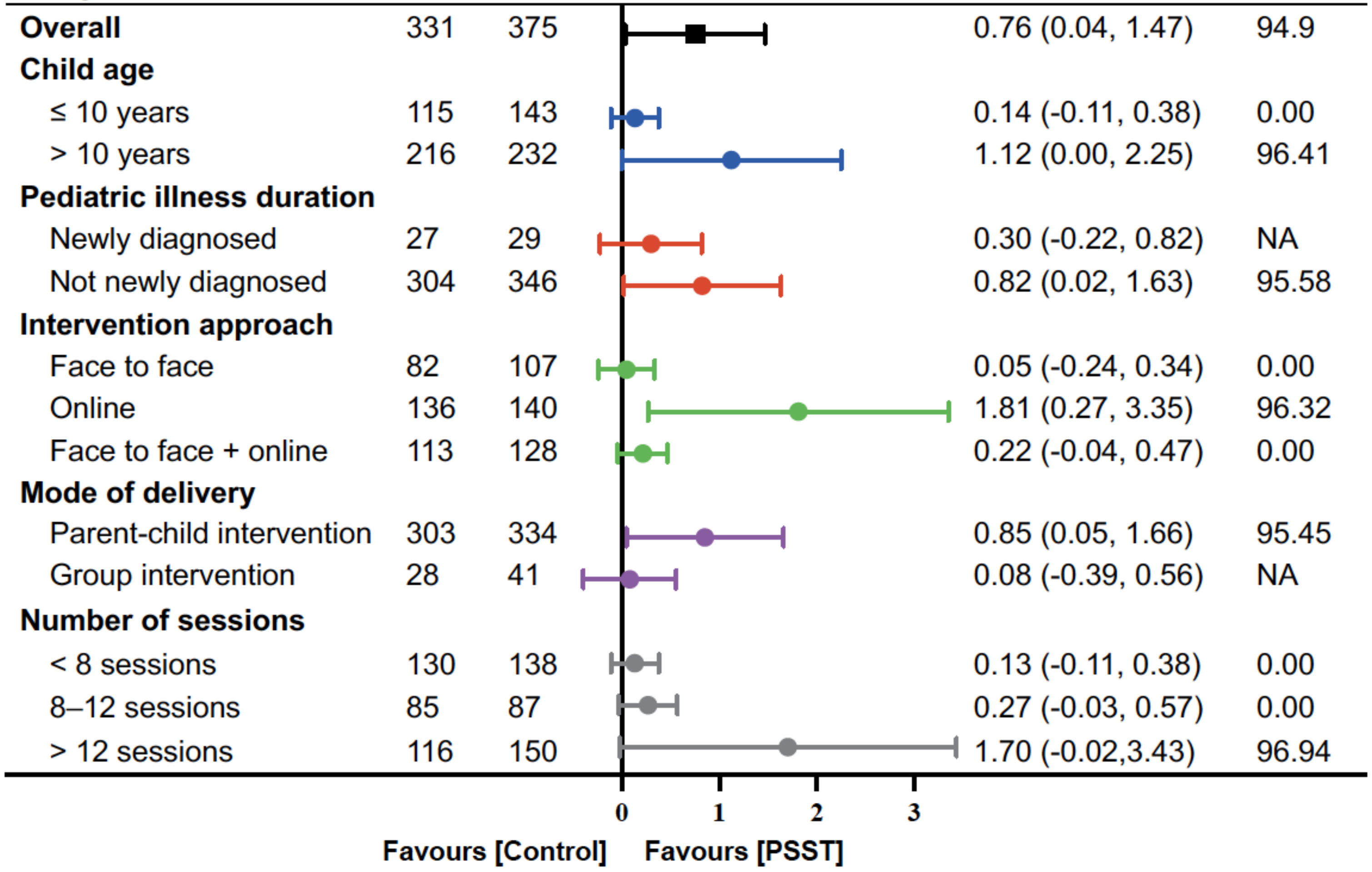


© 2024 American Medical Association. All rights reserved.

## eFigure 2.12 Subgroup Analysis of Childhood Medical Conditions on Pediatric Quality of Life Changes

| Study | Treatment N | Treatment Mean | Treatment SD | Control N | Control Mean | Control SD | Hedges's g with 95% CI | Weight (%) |
|---|---|---|---|---|---|---|---|---|
| **Asthma** | | | | | | | | |
| Seid 2010 | 60 | 81.4 | 16.3 | 73 | 80 | 12.4 | 0.10 [ -0.24, 0.44] | 12.85 |
| Heterogeneity: $\tau^2 = 0.00$, $I^2 = .\%$, $H^2 = .$ | | | | | | | 0.10 [ -0.24, 0.44] | |
| Test of $\theta_i = \theta_j$: Q(0) = 0.00, p = . | | | | | | | | |
| **Diabetes** | | | | | | | | |
| Nansel 2009 | 58 | 78.6 | 13.1 | 58 | 75.5 | 11.1 | 0.25 [ -0.11, 0.62] | 12.80 |
| Heterogeneity: $\tau^2 = 0.00$, $I^2 = .\%$, $H^2 = .$ | | | | | | | 0.25 [ -0.11, 0.62] | |
| Test of $\theta_i = \theta_j$: Q(0) = -0.00, p = . | | | | | | | | |
| **Epilepsy** | | | | | | | | |
| Modi 2021 | 27 | 84.08 | 12.5 | 29 | 79.84 | 15.2 | 0.30 [ -0.22, 0.82] | 12.37 |
| Heterogeneity: $\tau^2 = 0.00$, $I^2 = .\%$, $H^2 = .$ | | | | | | | 0.30 [ -0.22, 0.82] | |
| Test of $\theta_i = \theta_j$: Q(0) = 0.00, p = . | | | | | | | | |
| **Inflammatory bowel diseases (IBD)** | | | | | | | | |
| Greenley 2015 | 42 | 87.25 | 10.47 | 24 | 83.94 | 14.85 | 0.27 [ -0.23, 0.77] | 12.43 |
| Heterogeneity: $\tau^2 = 0.00$, $I^2 = .\%$, $H^2 = .$ | | | | | | | 0.27 [ -0.23, 0.77] | |
| Test of $\theta_i = \theta_j$: Q(0) = 0.00, p = . | | | | | | | | |
| **Sickle cell disease (SCD)** | | | | | | | | |
| Daniel 2015 | 28 | 2.94 | 20.07 | 41 | 1.14 | 22.11 | 0.08 [ -0.39, 0.56] | 12.50 |
| Heterogeneity: $\tau^2 = 0.00$, $I^2 = .\%$, $H^2 = .$ | | | | | | | 0.08 [ -0.39, 0.56] | |
| Test of $\theta_i = \theta_j$: Q(0) = -0.00, p = . | | | | | | | | |
| **Traumatic brain injury (TBI)** | | | | | | | | |
| Wade 2019 (a) | 22 | 62 | 3.35 | 34 | 62.2 | 3.05 | -0.06 [ -0.59, 0.47] | 12.34 |
| Wade 2019 (b) | 43 | 71.7 | 2.64 | 56 | 64.2 | 2.63 | 2.82 [ 2.27, 3.38] | 12.25 |
| Wade 2019 (c) | 51 | 70.9 | 2.82 | 60 | 64.3 | 2.76 | 2.35 [ 1.87, 2.83] | 12.48 |
| Heterogeneity: $\tau^2 = 2.25$, $I^2 = 96.94\%$, $H^2 = 32.65$ | | | | | | | 1.70 [ -0.02, 3.43] | |
| Test of $\theta_i = \theta_j$: Q(2) = 65.29, p = 0.00 | | | | | | | | |
| **Overall** | | | | | | | 0.76 [ 0.04, 1.47] | |
| Heterogeneity: $\tau^2 = 1.00$, $I^2 = 94.90\%$, $H^2 = 19.61$ | | | | | | | | |
| Test of $\theta_i = \theta_j$: Q(7) = 137.25, p = 0.00 | | | | | | | | |
| Test of group differences: $Q_b(5) = 3.80$, p = 0.58 | | | | | | | | |

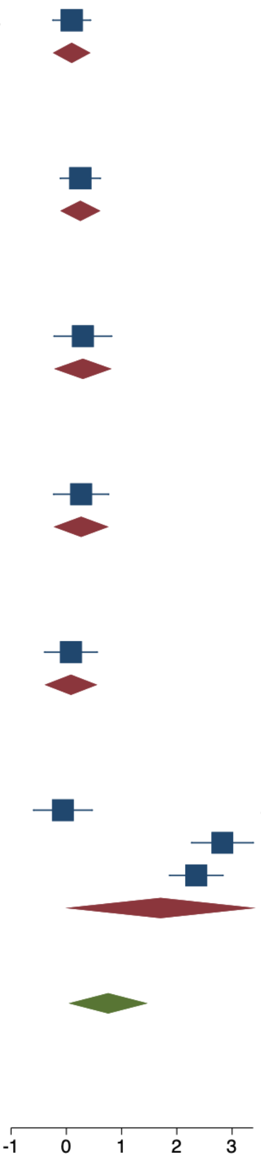


Random-effects DerSimonian-Laird model

© 2024 American Medical Association. All rights reserved.

## eFigure 2.13 Subgroup Analysis of PSST on Pediatric Mental Problems Changes (Overall)

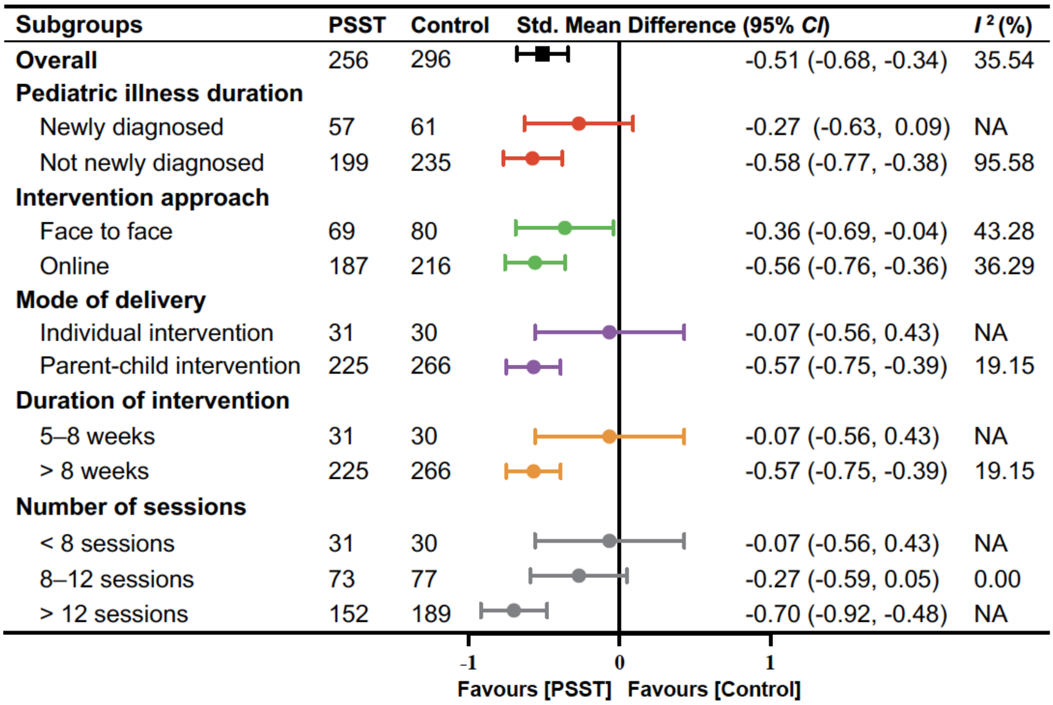


## eFigure 2.14 Subgroup Analysis of Childhood Medical Conditions on Pediatric Mental Problems Changes

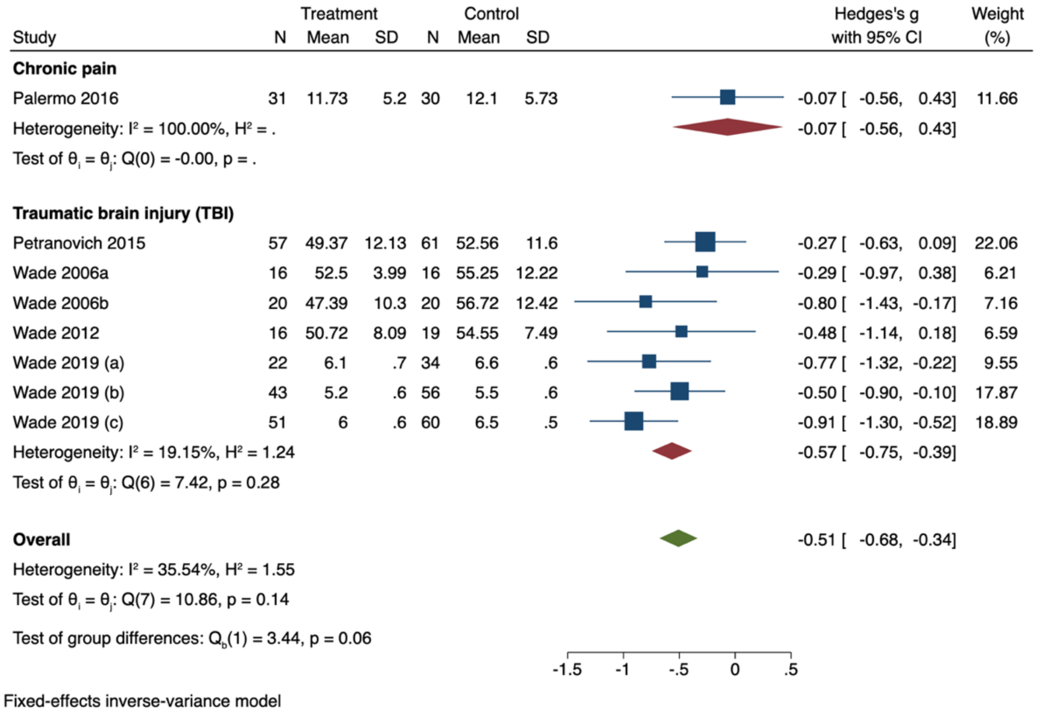


© 2024 American Medical Association. All rights reserved.

## eFigure 2.15 Subgroup Analysis of PSST on Parent-child Conflict Changes (Overall)

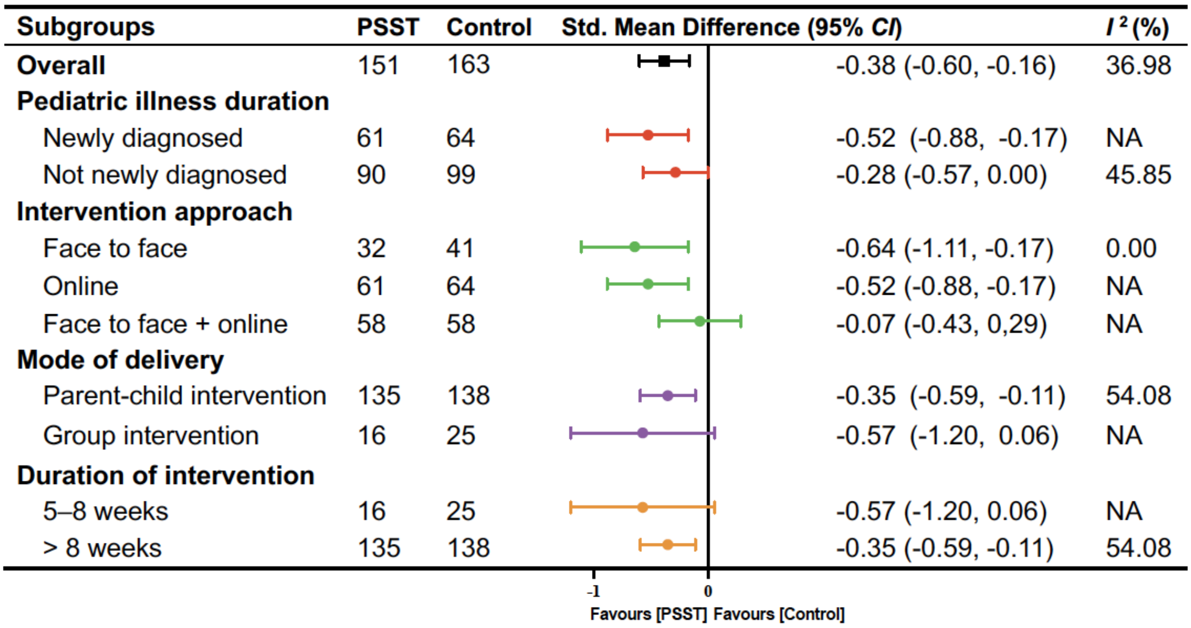

| Subgroups | PSST | Control | Std. Mean Difference (95% *CI*) | *I*² (%) |
|---|---|---|---|---|
| **Overall** | 151 | 163 | -0.38 (-0.60, -0.16) | 36.98 |
| **Pediatric illness duration** | | | | |
| Newly diagnosed | 61 | 64 | -0.52 (-0.88, -0.17) | NA |
| Not newly diagnosed | 90 | 99 | -0.28 (-0.57, 0.00) | 45.85 |
| **Intervention approach** | | | | |
| Face to face | 32 | 41 | -0.64 (-1.11, -0.17) | 0.00 |
| Online | 61 | 64 | -0.52 (-0.88, -0.17) | NA |
| Face to face + online | 58 | 58 | -0.07 (-0.43, 0,29) | NA |
| **Mode of delivery** | | | | |
| Parent-child intervention | 135 | 138 | -0.35 (-0.59, -0.11) | 54.08 |
| Group intervention | 16 | 25 | -0.57 (-1.20, 0.06) | NA |
| **Duration of intervention** | | | | |
| 5–8 weeks | 16 | 25 | -0.57 (-1.20, 0.06) | NA |
| > 8 weeks | 135 | 138 | -0.35 (-0.59, -0.11) | 54.08 |



## eFigure 2.16 Subgroup Analysis of Childhood Medical Conditions on Parent-child Conflict Changes

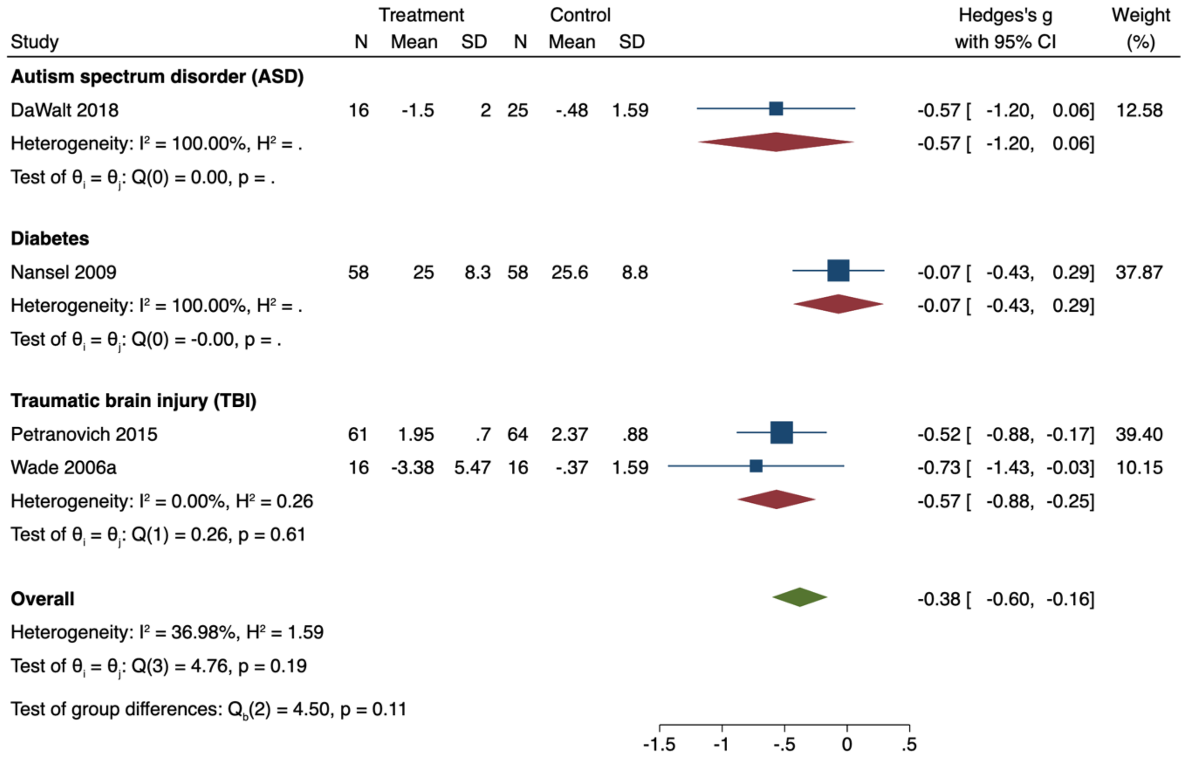

| Study | Treatment N | Treatment Mean | Treatment SD | Control N | Control Mean | Control SD | Hedges's g with 95% CI | Weight (%) |
|---|---|---|---|---|---|---|---|---|
| **Autism spectrum disorder (ASD)** | | | | | | | | |
| DaWalt 2018 | 16 | -1.5 | 2 | 25 | -.48 | 1.59 | -0.57 [ -1.20, 0.06] | 12.58 |
| Heterogeneity: $I^2$ = 100.00%, $H^2$ = . | | | | | | | -0.57 [ -1.20, 0.06] | |
| Test of $\theta_i = \theta_j$: Q(0) = 0.00, p = . | | | | | | | | |
| **Diabetes** | | | | | | | | |
| Nansel 2009 | 58 | 25 | 8.3 | 58 | 25.6 | 8.8 | -0.07 [ -0.43, 0.29] | 37.87 |
| Heterogeneity: $I^2$ = 100.00%, $H^2$ = . | | | | | | | -0.07 [ -0.43, 0.29] | |
| Test of $\theta_i = \theta_j$: Q(0) = -0.00, p = . | | | | | | | | |
| **Traumatic brain injury (TBI)** | | | | | | | | |
| Petranovich 2015 | 61 | 1.95 | .7 | 64 | 2.37 | .88 | -0.52 [ -0.88, -0.17] | 39.40 |
| Wade 2006a | 16 | -3.38 | 5.47 | 16 | -.37 | 1.59 | -0.73 [ -1.43, -0.03] | 10.15 |
| Heterogeneity: $I^2$ = 0.00%, $H^2$ = 0.26 | | | | | | | -0.57 [ -0.88, -0.25] | |
| Test of $\theta_i = \theta_j$: Q(1) = 0.26, p = 0.61 | | | | | | | | |
| **Overall** | | | | | | | -0.38 [ -0.60, -0.16] | |
| Heterogeneity: $I^2$ = 36.98%, $H^2$ = 1.59 | | | | | | | | |
| Test of $\theta_i = \theta_j$: Q(3) = 4.76, p = 0.19 | | | | | | | | |
| Test of group differences: $Q_b(2)$ = 4.50, p = 0.11 | | | | | | | | |



Fixed-effects inverse-variance model

© 2024 American Medical Association. All rights reserved.

**eFigure 3. Funnel Plot Analyses**

**eFigure 3.1 Funnel Plot for Parental Depression**

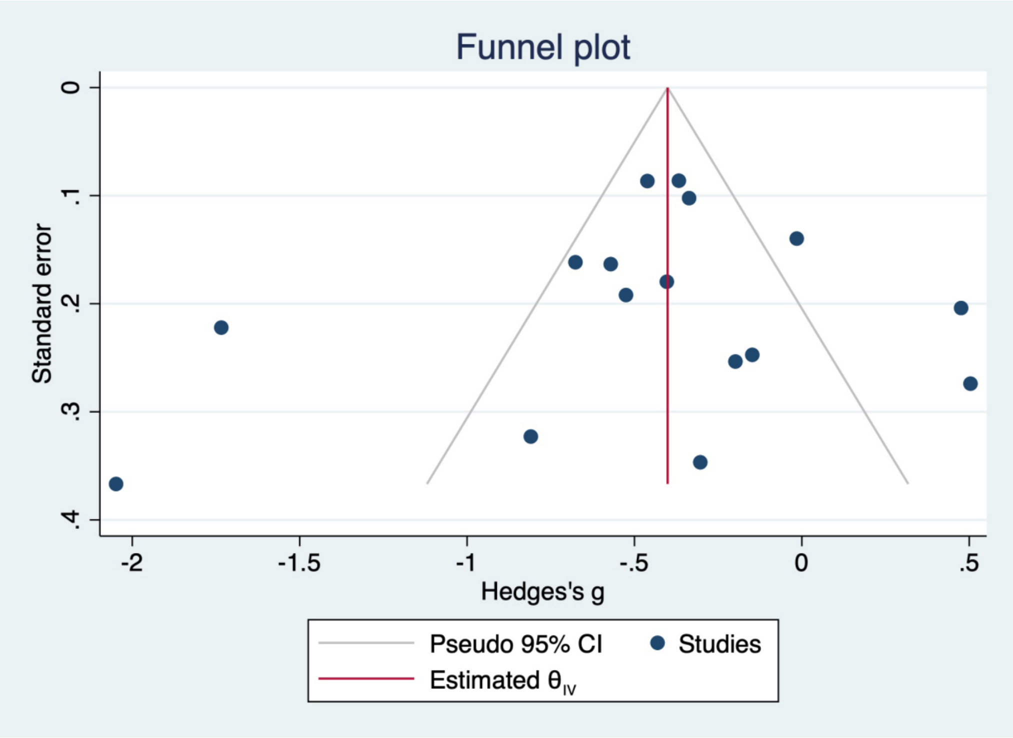


**eFigure 3.2 Funnel Plot for Parental Distress**

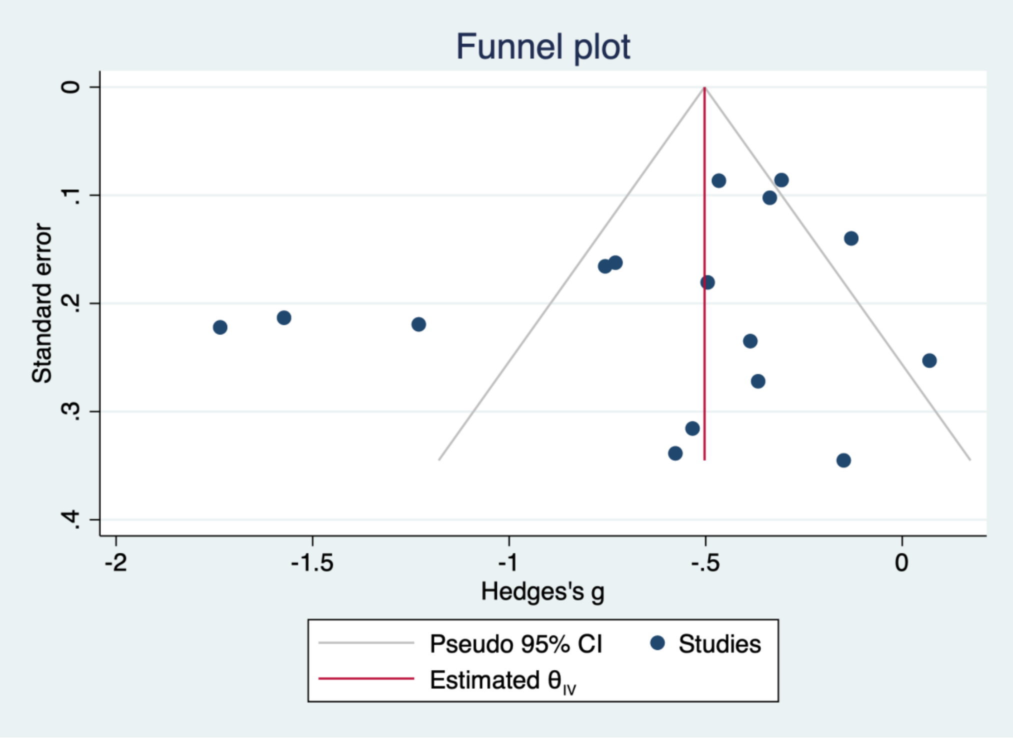


© 2024 American Medical Association. All rights reserved.

**eFigure 3.3 Funnel Plot for Parental Problem-solving Skills**

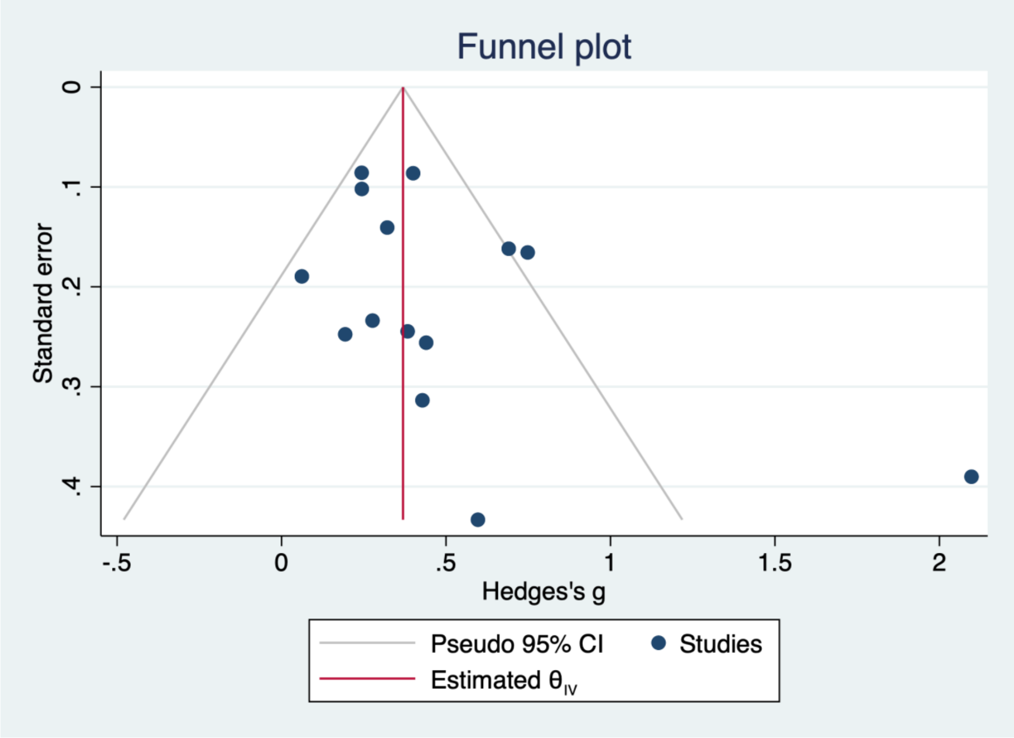


© 2024 American Medical Association. All rights reserved.

## eFigure 4. Leave-One-Out Sensitivity Analyses

### eFigure 4.1 Leave-one-out Sensitivity Analysis for Parental Depression

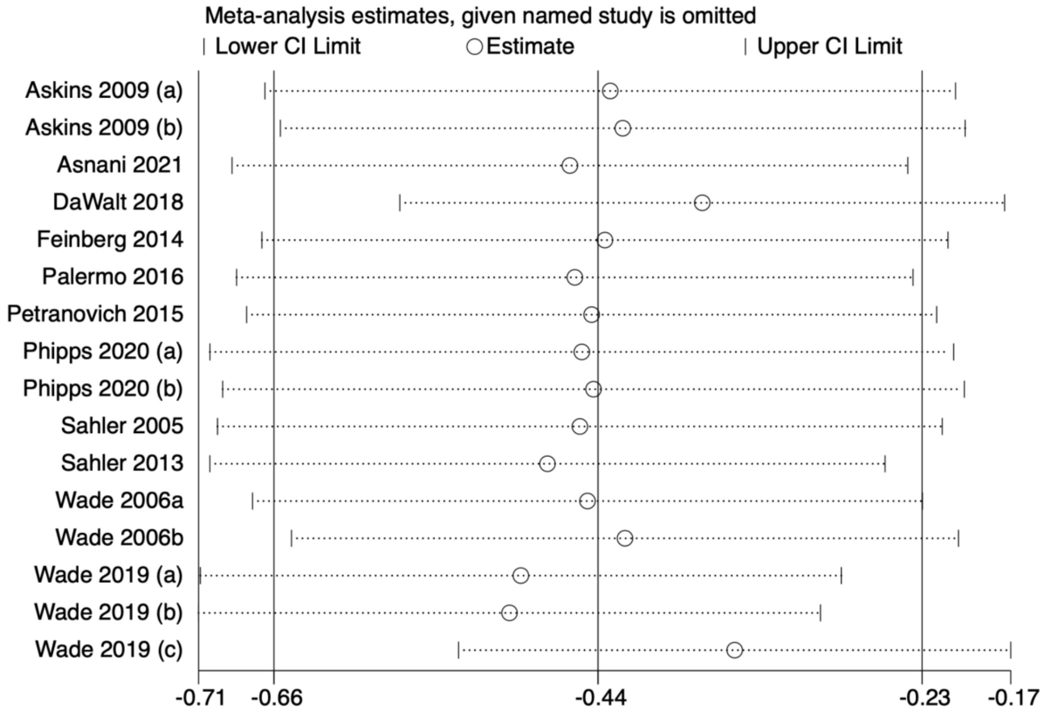


### eFigure 4.2 Leave-one-out Sensitivity Analysis for Parental Distress

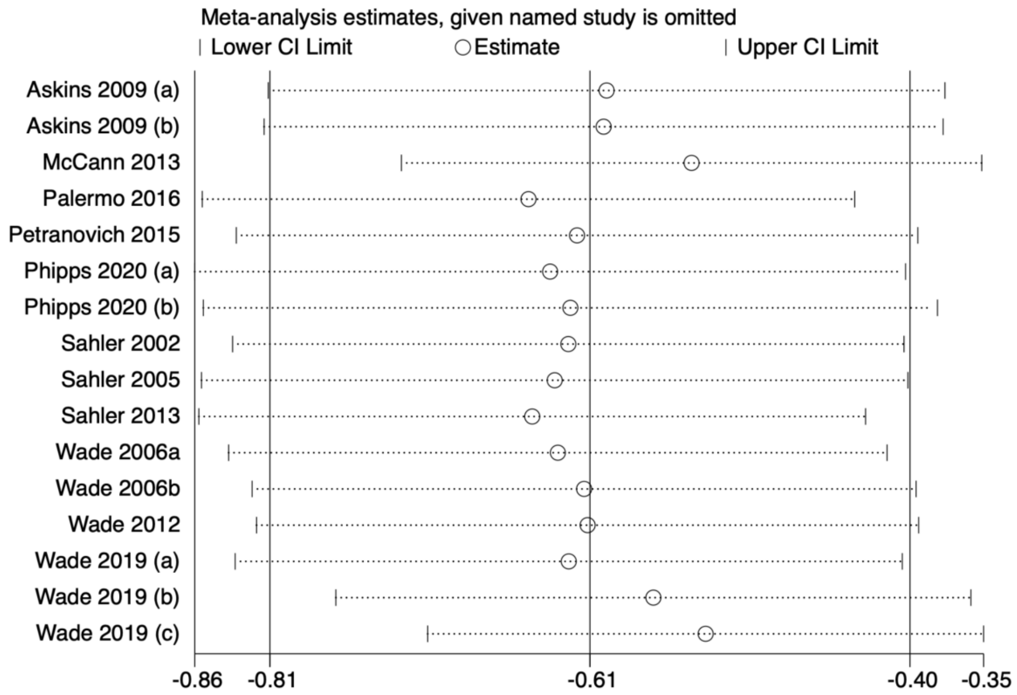


© 2024 American Medical Association. All rights reserved.

**eFigure 4.3 Leave-one-out Sensitivity Analysis for Parental Problem-solving Skills**

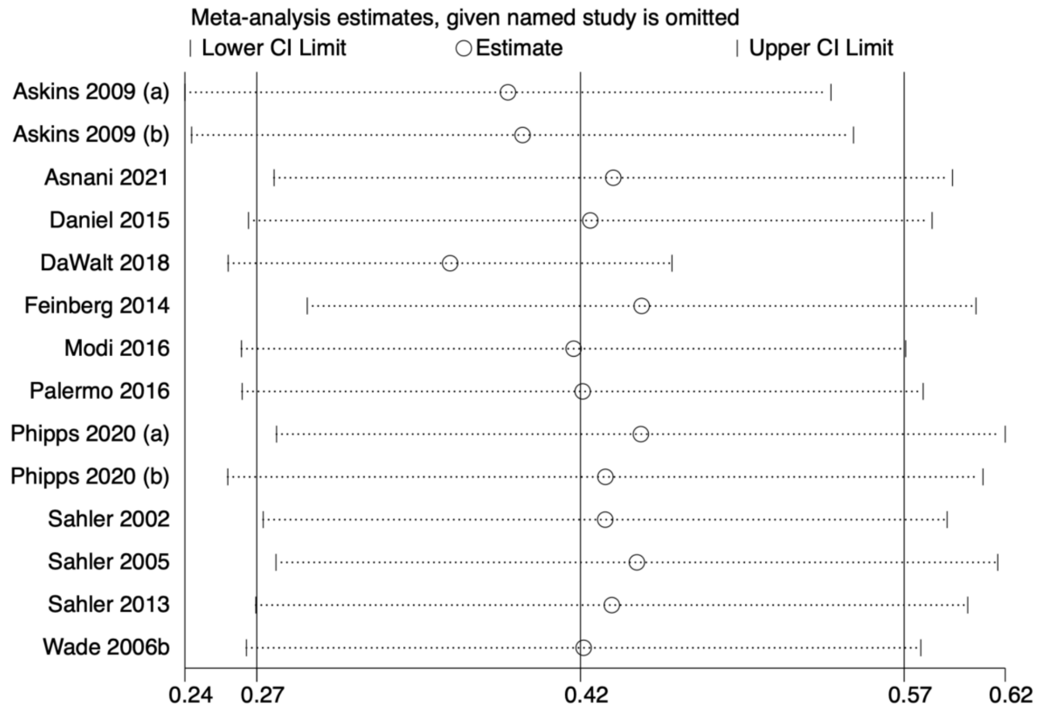


**eFigure 4.4 Leave-one-out Sensitivity Analysis for Parental Parental Post-traumatic Stress**

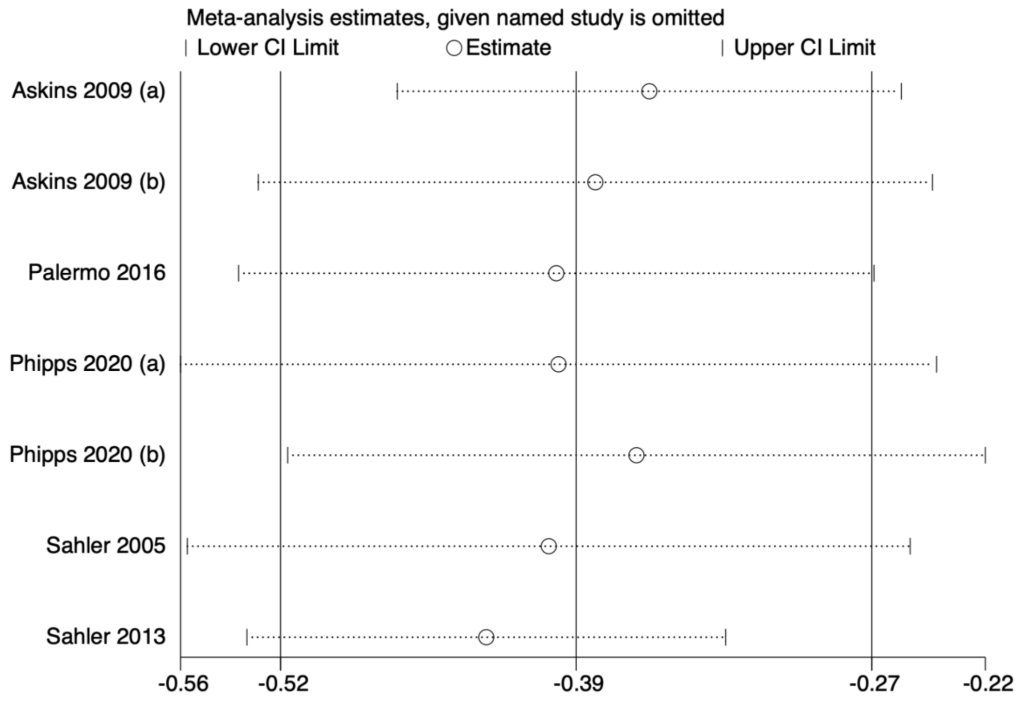


© 2024 American Medical Association. All rights reserved.

## eFigure 4.5 Leave-one-out Sensitivity Analysis for Parenting Stress

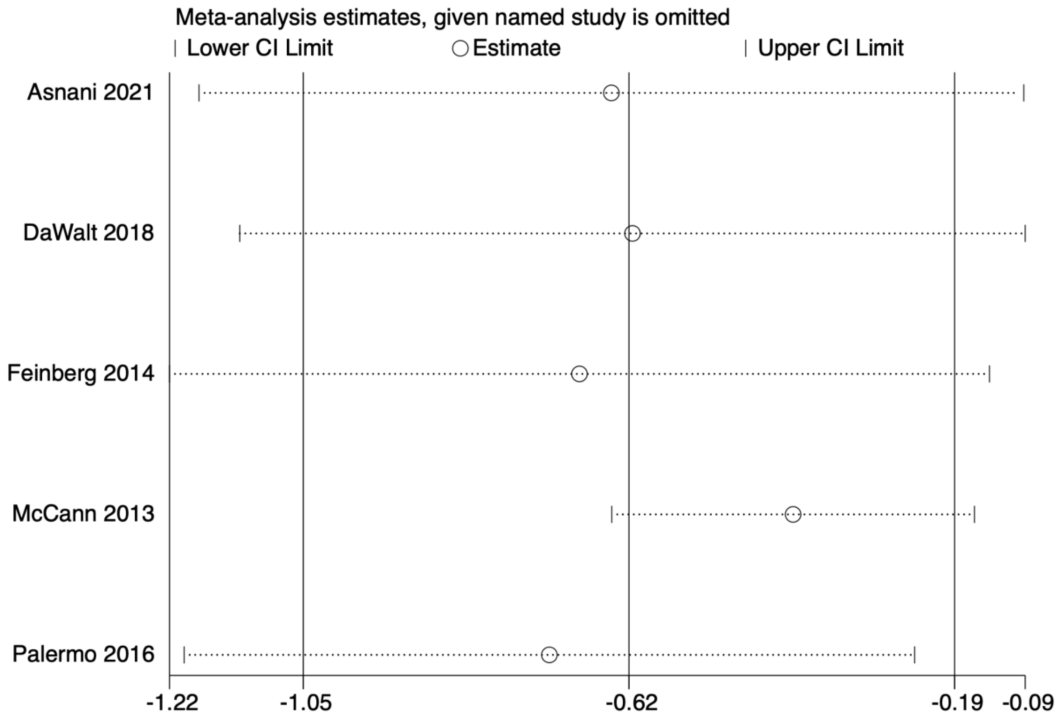


## eFigure 4.6 Leave-one-out Sensitivity Analysis for Parental Anxiety

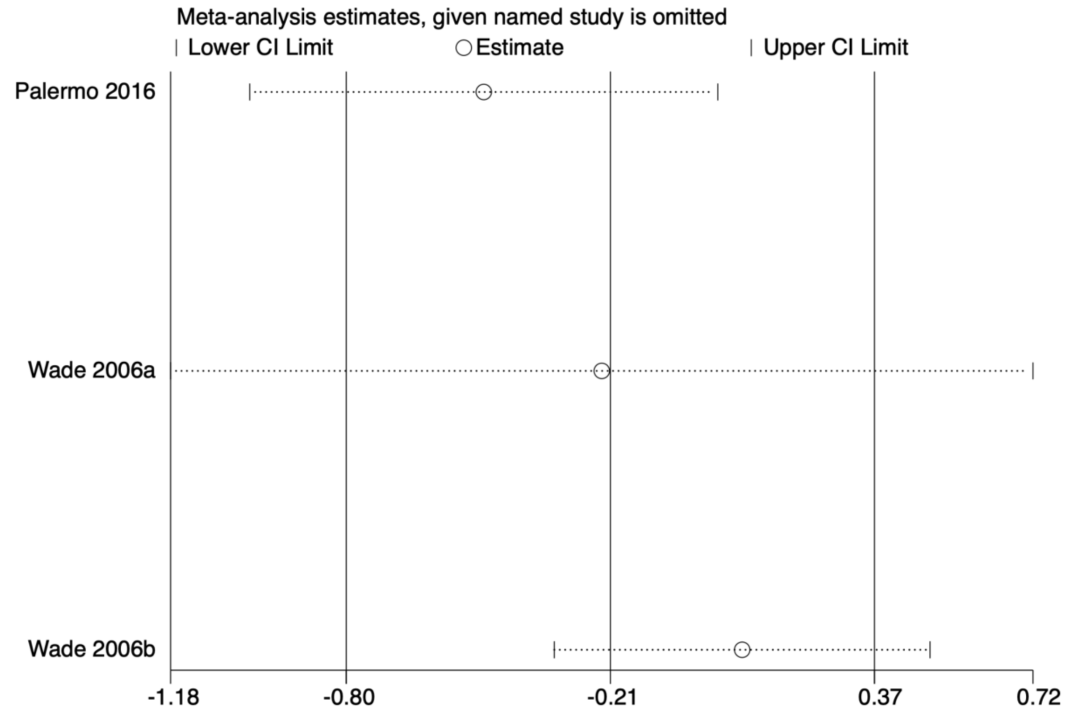


© 2024 American Medical Association. All rights reserved.

**eFigure 4.7 Leave-one-out Sensitivity Analysis for Pediatric Quality of Life**

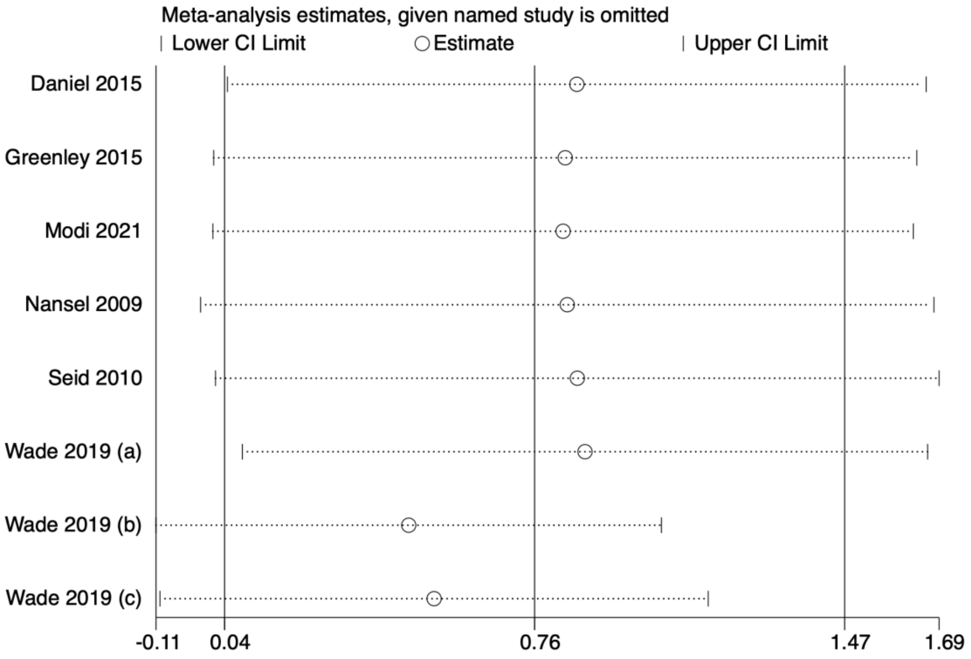


**eFigure 4.8 Leave-one-out Sensitivity Analysis for Pediatric Mental Problems**

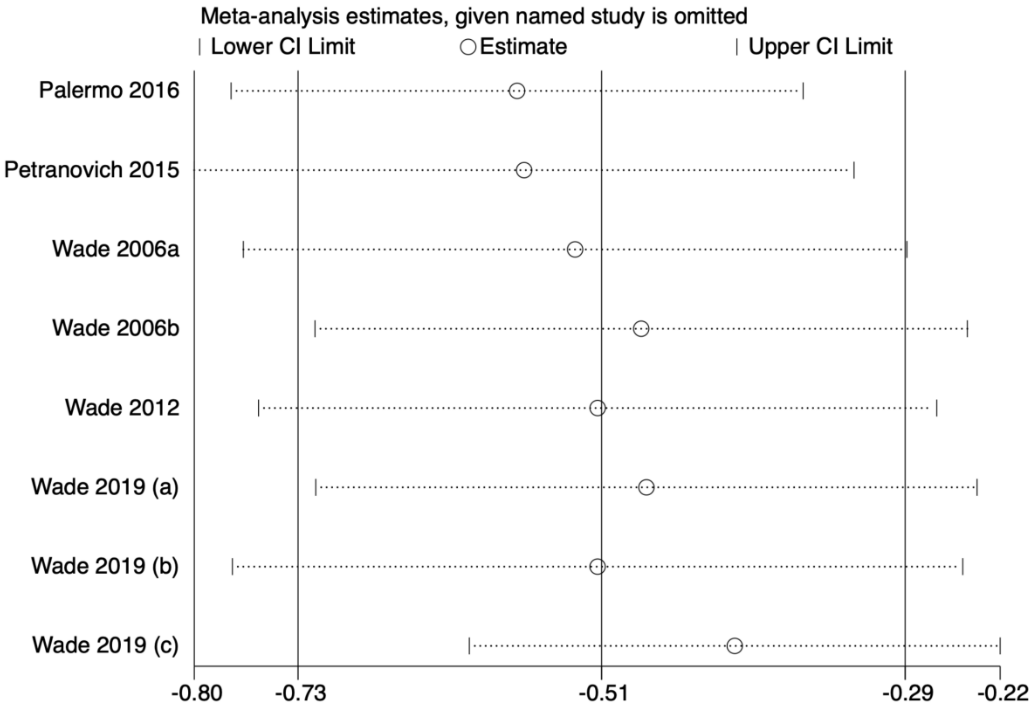


© 2024 American Medical Association. All rights reserved.

**eFigure 4.9 Leave-one-out Sensitivity Analysis for Pediatric Social Functioning**

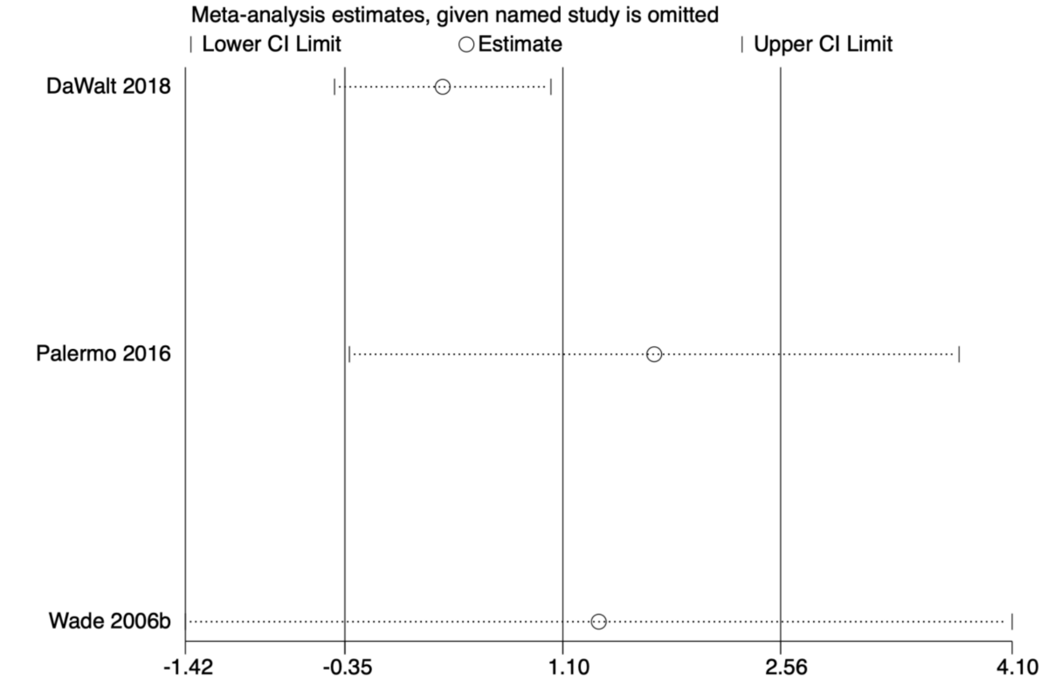


**eFigure 4.10 Leave-one-out Sensitivity Analysis for Parent-child Conflict**

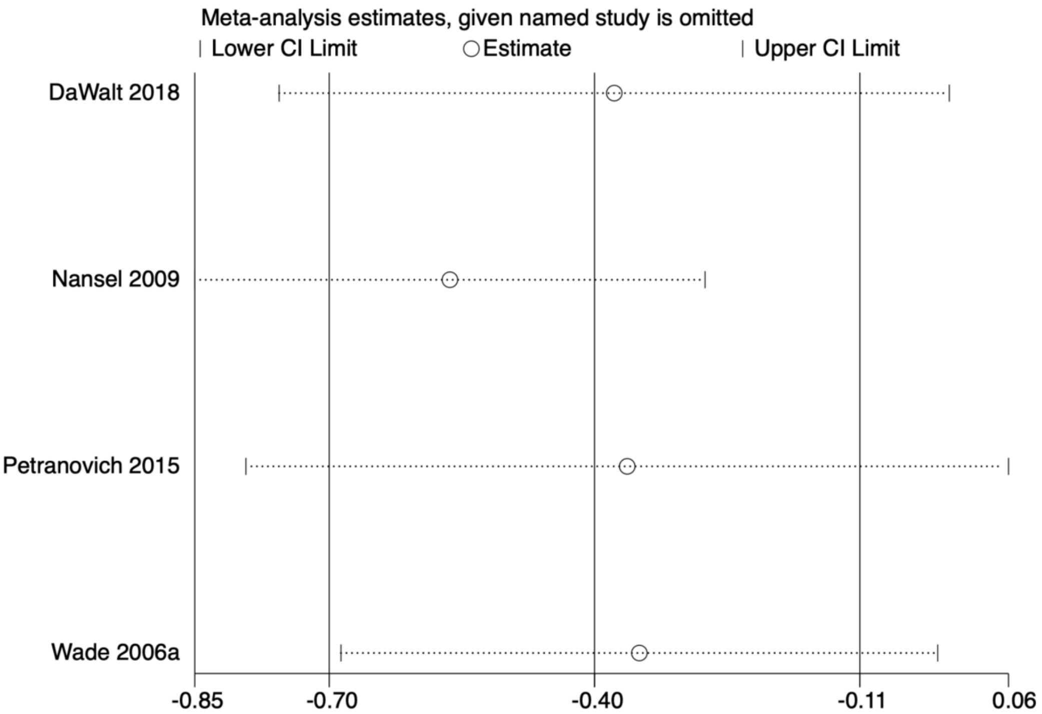


© 2024 American Medical Association. All rights reserved.
