# Supplementary material for: Problem-Solving Skills Training for Parents of Children With Chronic Health Conditions: A Systematic Review and Meta-Analysis
Source: JAMA Pediatr. 2024 Jan 2;178(3):226–36. doi: 10.1001/jamapediatrics.2023.5753 (PMC10762633; doi:10.1001/jamapediatrics.2023.5753)
Supplement: Supplement 2. — Data Sharing Statement [file jamapediatr-e235753-s002.pdf]

Data Sharing Statement

Zhou. Problem-Solving Skills Training for Parents of Children With Chronic Health Conditions. *JAMA Pediatr*. Published January 02, 2024. doi:10.1001/jamapediatrics.2023.5753

Data
Data available: No

Additional Information
Explanation for why data not available: All data for this systematic review and meta-analysis were obtained from published trials.
